# The Biology of Veganism: Plasma Metabolomics Analysis Reveals Distinct Profiles of Vegans and Non-Vegetarians in the Adventist Health Study-2 Cohort

**DOI:** 10.3390/nu14030709

**Published:** 2022-02-08

**Authors:** Fayth L. Miles, Michael J. Orlich, Andrew Mashchak, Paulette D. Chandler, Johanna W. Lampe, Penelope Duerksen-Hughes, Gary E. Fraser

**Affiliations:** 1 Adventist Health Study, Research Affairs, Loma Linda University, Loma Linda, CA 92350, USA; fmiles@llu.edu (F.L.M.); morlich@llu.edu (M.J.O.); amashchak@llu.edu (A.M.); 2 Center for Nutrition, Healthy Lifestyle and Disease Prevention, School of Public Health, Loma Linda University, Loma Linda, CA 92350, USA; 3 Department of Preventive Medicine, School of Medicine, Loma Linda University, Loma Linda, CA 92350, USA; 4 Department of Basic Science, School of Medicine, Loma Linda University, Loma Linda, CA 92350, USA; pdhughes@llu.edu; 5 Division of Preventive Medicine, Brigham and Women’s Hospital, Boston, MA 02215, USA; pchandler@bwh.harvard.edu; 6 Public Health Sciences Division, Cancer Prevention Program, Fred Hutchinson Cancer Research Center, Seattle, WA 98109, USA; jlampe@fredhutch.org; 7 Department of Medicine, School of Medicine, Loma Linda University, Loma Linda, CA 92350, USA

**Keywords:** vegetarian, metabolomics, cohort, biomarkers, dietary pattern, linear regression, false discovery

## Abstract

It is unclear how vegetarian dietary patterns influence plasma metabolites involved in biological processes regulating chronic diseases. We sought to identify plasma metabolic profiles distinguishing vegans (avoiding meat, eggs, dairy) from non-vegetarians (consuming ≥28 g/day red meat) of the Adventist Health Study-2 cohort using global metabolomics profiling with ultra-performance liquid chromatography mass spectrometry (UPLC-MS/MS). Differences in abundance of metabolites or biochemical subclasses were analyzed using linear regression models, adjusting for surrogate and confounding variables, with cross-validation to simulate results from an independent sample. Random forest was used as a learning tool for classification, and principal component analysis was used to identify clusters of related metabolites. Differences in covariate-adjusted metabolite abundance were identified in over 60% of metabolites (586/930), after adjustment for false discovery. The vast majority of differentially abundant metabolites or metabolite subclasses showed lower abundance in vegans, including xanthine, histidine, branched fatty acids, acetylated peptides, ceramides, and long-chain acylcarnitines, among others. Many of these metabolite subclasses have roles in insulin dysregulation, cardiometabolic phenotypes, and inflammation. Analysis of metabolic profiles in vegans and non-vegetarians revealed vast differences in these two dietary groups, reflecting differences in consumption of animal and plant products. These metabolites serve as biomarkers of food intake, many with potential pathophysiological consequences for cardiometabolic diseases.

## 1. Introduction

Findings from the Adventist Health Study-2 Cohort have demonstrated that vegetarian dietary patterns have been associated with many positive health outcomes, including lower risk of metabolic syndrome (56%) [1], lower incidence of diabetes (39–62%) [2], lower overall mortality (hazard ratio (HR): 0.88, 95% confidence interval (CI): 0.80–0.97), and cardiovascular disease mortality (HR: 0.71, 95% CI: 0.57–0.90) for males [3], as well as lower risk of gastrointestinal cancers (HR: 0.76, 95% CI: 0.63–0.90) and cancer overall (HR: 0.92, 95% CI: 0.85–0.99) [4,5]. Vegans, particularly have lower risk of female-specific cancers (HR: 0.66, 95% CI: 0.47–0.92) [5], prostate cancer (HR: 0.65; 95% CI: 0.49, 0.85) [6], and cancer overall (HR: 0.84; 95% CI: 0.72–0.99) [5] relative to non-vegetarians. Metabolic pathways associated with such health-promoting dietary patterns, and particularly a vegan diet, remain to be elucidated.

The metabolome may be modulated by select phytochemical-rich plant-based foods. Many studies have shown higher abundance of potentially beneficial metabolites with higher consumption of fruits and vegetables. Metabolic profiles have been compared in subjects adhering to western or prudent dietary patterns in cross-sectional studies, where differences in plasmalogens, phosphatidylcholines, cholesteryl esters, and acylcarnitines, among other compounds, were reported [7,8]. Additionally, a Mediterranean dietary pattern or score has been associated with changes in carbohydrate and lipid metabolites including phosphatidylcholines and acylcarnitines, amino acids, biogenic amines, xenobiotics, and microbial cometabolites [9,10,11]. Metabolic profiles have also been characterized and compared among dietary patterns defined by various healthy eating indices [9,12].

Few studies have compared metabolic profiles between habitual vegetarians, especially vegans, and omnivores. Long-term, habitual dietary patterns may be associated with more stable alterations in the metabolome. In one plasma metabolomics study where omnivores were compared to vegans and other vegetarians, omnivores were found to have highest concentrations of lipid metabolites, acylcarnitines, glycerophospholipids, and sphingolipids, with many of these mediating biological processes relevant to various pathophysiological conditions [13]. Vegans have also been found to have higher abundance of metabolites related to microbial and polyphenol metabolism in spite of minimal differences in gut microbial composition [14].

We previously measured biomarkers of dietary intake in biospecimens from vegetarians and non-vegetarians in the Adventist Health Study-2 (AHS-2) cohort participating in a calibration study (*n* = 1011), and reported notable differences, including increased abundance of phytochemicals and healthier profiles of fatty acids in the vegetarians [15]. We also recently reported differences in DNA methylation patterns between vegans and non-vegetarians, noting some alterations in genes with relevance to cancer development [16]. It is probable that dietary profiles characterized by high consumption of phytochemical and polyphenolic compounds present in plant-based foods stimulate metabolic pathways associated with disease prevention.

Metabolomics may prove to be very helpful in elucidating diet-mediated biochemical functions by linking eating patterns with the pathophysiology of chronic diseases. In light of our previous findings associating vegetarian dietary patterns with reduced risk of chronic conditions and metabolic diseases, we sought to analyze differences in profiles of metabolic compounds comparing vegans to non-vegetarians in the AHS-2 cohort, using an untargeted metabolomics approach.

## 2. Materials and Methods

### 2.1. Study Design

The AHS-2 cohort (established 2002–2007) consists of over 96,000 Seventh Day Adventists age ≥29, with roughly 55% distributed among vegan (8–9%), and lacto-ovo-(29%), pesco-(10%) and semi-non-vegetarian diet groups (5–6%), and the remainder categorized as non-vegetarians (45%). The cohort was established to examine the influence of diet and lifestyle on various health outcomes including cancer, cardiovascular disease, obesity, diabetes, and total mortality. All participants completed a food frequency questionnaire (FFQ) at enrollment (2002–2007), which was used to assign dietary status [17]. The FFQ was a self-administered semi-quantitative instrument collecting basic demographic information including sex, age, and ethnicity, and assessing dietary habits over the past year. The FFQ collected information on frequency of consumption of over 200 foods, including fresh, cooked or canned fruits and vegetables, legumes (lentils, soybeans, and other beans), breads and grains, soy foods/drinks/supplements, dairy, eggs, red meats, processed meats, poultry, and fish consumption, among other items. Frequency categories varied with food type, and portion sizes included three levels: standard, ½ or less, and 1½ or more. Nutrient intake data were collected with the use of the Nutrition Data System for Research software versions 4.06 and 5.03 (The Nutrition Coordinating Center, University of Minnesota, Minneapolis, MN, USA) [18]. Validity of dietary intake has been assessed extensively using 24-h diet recalls and biomarkers [17], and methods developed for dealing with measurement error [17,19,20]. Biennial health and hospitalization history forms (HHF) captured changes in exposures as well as lifetime dietary pattern trends for AHS-2 members [21]. The current study included vegans and non-vegetarians from the AHS-2 cohort who were previously recruited to participate in one of various substudies, where they were asked to attend clinic and provide blood, urine, saliva, and/or adipose samples. These included a calibration study [17], Biological Manifestations of Religion Substudy (BioMRS), which was nested within the Biology, Religion, and Health substudy [22] and other pilot studies (Supplementary Materials, Figure S1). For the calibration and pilot studies, fasting blood samples were collected at field clinics held in church halls, as described previously, where healthy participants were selected randomly by church (Adventist churches were randomly selected from those within the US and Canada, weighted by church size) and then subject within church [23], while BioMRS participants were recruited to clinic sites in Loma Linda, Riverside, and Los Angeles, CA, USA [22]. Anthropometric data were also collected during clinic visits. For the current cross-sectional study, 96 subjects, including vegans and non-vegetarians were selected with stratified random sampling, balancing by sex and race, and excluding individuals with extreme body mass index (BMI) (<14 or >50) and total caloric intake of <500 or >4500 kcal/day. Vegans were defined as never or rarely (less than once per month) consuming eggs, dairy, fish, and other meats, based on responses to the FFQ. Non-vegetarians were defined as consuming non-fish meats at least once a month and any meat (including but not only fish) more than once per week. Vegans were of primary interest for this pilot study because of their complete avoidance of animal products and high consumption of fruits, vegetables, whole grains, legumes, and soy [24]. Non-vegetarians, who consume relatively low amounts of non-fish meats relative to the general population, were selected only if red meat consumption was ≥28 g/day (1 ounce) as an attempt to maximize the contrast between the two groups, although the majority (>70%) consumed more than 56 g/day (2 ounces). Viable plasma samples from a total of 46 non-vegetarians and 47 vegans were included in the current study for metabolomics profiling. The procedures followed were in accordance with the ethical standards of Loma Linda University and approved by the institutional review board for research involving human subjects. 

### 2.2. Metabolomics Profiling

Ninety-three heparin plasma samples were analyzed by Metabolon Inc. (Durham, NC, USA). Samples were profiled using a Global Assay (DiscoveryHD4) (Metabolon Inc., Durham, NC, USA) that provides a comprehensive picture of metabolites within central carbon metabolism and various biochemical classes/pathways including amino acid, nucleotide, carbohydrate, lipid, xenobiotic, microbial, and others. The Metabolon platform yielded 1017 compounds of known identity.

Samples were prepared using the automated MicroLab STAR^®^ system from Hamilton company (Reno, NV, USA), as described previously [25]. After precipitation of proteins, the resulting extracts were analyzed on four independent ultra-high-performance liquid chromatography-tandem mass spectroscopy (UPLC-MS/MS) platforms. A thorough description of the metabolic platform and quality control procedures have been described previously [25]. Various controls were used in tandem with experimental samples, prior to injection into the mass spectrometer. A cocktail of QC standards was added to each sample to monitor instrument performance and aid in chromatographic alignment. Aliquots of a pooled plasma sample (obtained by taking a small amount of each experimental sample) were included as technical replicates. Extracted water samples served as process blanks. Experimental samples were randomized across the platform run with QC samples spaced evenly among the injections. Biochemical compounds were identified by comparison to library entries of purified, authenticated standards or recurrent unknown entities. The median relative standard deviation (RSD) was calculated for the standards added to each sample as a measure of instrument variability, as well as for all endogenous metabolites within the pooled plasma samples (non-instrument standards) as a measure of overall process variability. The median RSD for internal standards and endogenous metabolites was 6% and 14%, respectively. Samples were measured in one batch and randomized by diet group.

### 2.3. Statistical Analysis

#### 2.3.1. Data Transformation and Linear Regression

Raw metabolite values were median scaled (divided by the median value), and missing metabolite values (below the detection threshold) were imputed with the minimum value for a given metabolite. Data was subsequently log transformed. Metabolites that were below the detection limit for >50% vegans and omnivores were excluded from the analysis, yielding an analytical set of 930 metabolites.

Linear regression models were generated to determine if individual plasma metabolites differed between vegans and non-vegetarians, using smart surrogate variable analysis (SmartSVA). With this approach, regression residuals, obtained from regression of log-transformed metabolite abundance (response) on diet group and covariates—age at blood collection, sex (male vs. female), race (Black vs. White), and BMI (continuous)—were used to obtain surrogate variables representing other unknown, unwanted sources of variation. A linear model was then fitted where the dependent and independent variables were the residuals obtained from regressing metabolite abundance, and dietary pattern, respectively, on surrogate variables and the other covariates, excluding BMI, which was considered as a mediating variable. Models including energy intake, which differed between vegans and non-vegetarians at baseline, did not show appreciable changes in results, so energy intake was not included in final models. The resulting univariate beta coefficients and adjusted predicted means with 95% confidence intervals were then obtained for each metabolite. The adjusted means estimate log (geometric means of untransformed data), and the difference in these between vegans to non-vegetarians, are estimates of log (fold change) metabolite abundance. Linear regression models were also generated without SmartSVA, regressing metabolite abundance on dietary pattern, adjusting only for age, sex, and race. 

An analysis of differential abundance of metabolites at the subclass level used the same approach, but operated on the average of the numerators of the individual metabolite t statistics, i.e., regression beta coefficients that contributed to that subclass. Composite t-statistics were produced by dividing by the standard deviations of the averaged numerators taking account of the covariances between subclass members. Each component metabolite had been measured as a multiple of its own median, then log-transformed. The differences between predicted means of subclass members in vegans compared to non-vegetarians were calculated. These estimated logs (the ratio of composite geometric means of untransformed values) were finally exponentiated to produce estimated fold changes. All statistical analyses were performed in R Statistical Software (version 4.0.2; R Core Team, Vienna, Austria).

#### 2.3.2. Adjustment for False Discovery

The partial t-statistics from the regressions described above correspond to differences in metabolite abundance according to dietary pattern. To control for multiple testing, an adapted Storey et al. [26] permutation approach was used. The residualized dietary pattern variables were permuted as a means of defining the null distribution of the t scores for metabolite abundance [27], thereby retaining covariances between residualized metabolite abundances. Estimating the proportion of null metabolites allows an estimate of the false discovery rate (FDR) avoiding the over-conservative Benjamini–Hochberg approach [26] and the consequent selection of metabolites with small FDR. An identical procedure was also used at the subclass level.

#### 2.3.3. Principal Components Analysis

Principal components analysis (PCA) was performed using the FactoMineR package in R on 930 log-transformed metabolites as a dimension reduction approach to identify principal components (PCs) or axes that maximize/explain the variation in metabolite abundance. This was done to test the hypothesis that a vegan or non-vegetarian diet could be defined by select groups of metabolites. A PCA plot was generated by obtaining individual scores (coordinates) of vegans and non-vegetarians for top PCs (PC1 and PC3) in 92 subjects, excluding one outlier. Ten PCs explained 50% of the variance. These were retained for regression analysis (eigenvalues >20), where associations of the 12 PCs with diet group and other dietary covariates (energy-adjusted) of interest were examined. Partial correlation coefficients between PCs and diet group or other variables of interest were also obtained (using ppcor package in R). For the PCA regression, variables with missing values (BMI, *n* = 2; Kcal, *n* = 1) were imputed with the mean value. Any missing dietary data was handled using multiple imputation with appropriate standard errors. Metabolites with loadings of 0.5 or higher (representing metabolite correlations with PCs) were identified for 10 components. 

#### 2.3.4. Random Forest Analysis

Random forest analysis was also used as a supervised approach for classifying metabolites to identify the most informative metabolites distinguishing vegans from non-vegetarians. All 930 log transformed metabolites were considered in the analysis, with each of 50,000 trees learning from a random sample of fifty percent of all the data (23 vegans and 23 non-vegetarians) without replacement, and the remaining data (representing the out of bag variables) passed down the tree for class prediction to calculate the out of bag (OOB) error. Mean decrease in accuracy was calculated by randomly permuting a variable (metabolite), and subsequently passing the data down the trees for re-assessment of class prediction. Hence, the most influential metabolites were determined after permuting each predictor variable and measuring the change or decrease in predictive accuracy [28].

#### 2.3.5. Bootstrap Regression

Our bootstrap procedure chose 930 subjects with replacement, and then in each of 100 such choices performed the FDR analysis. At the metabolite level, 129 of the 930 metabolites were significant (FDR < 0.05) in at least 90% of all bootstrap samples (44 significant 100% of samples). At the subclass level, there were 18 significant (FDR < 0.05) at least 90% of the time, and 9 significant in 100% of the samples. The metabolites and subclasses are listed in the Supplementary Materials (Tables S16–S19).

While the FDR < 0.05, should be a relatively unbiased estimator of the fact that only 5% of such metabolites will be erroneously selected as significant, it gives no information about truly differential metabolites that by chance had a t-score that just missed the FDR cut-point (Type II error). This was assessed by identifying metabolites significant in >50% of bootstrap samples. The bootstrap result provided added information by tending to identify metabolites that may be part of the 5% of false positives (they would show significance in few samples), while also identifying some metabolites that by chance missed significance in the parent sample, but achieved it in many of the bootstrap samples. 

#### 2.3.6. Cross-Validation

A separate consideration is that the regressions run to adjust for confounding are somewhat overoptimistic in the t-scores that they produce for the beta coefficients, as likelihoods to some extent are being maximized to also reflect random idiosyncrasies of our sample. This can be largely overcome by a cross-validation procedure, as follows: Randomly, divide the participant sample to K parts. Excluding one part at a time, develop a regression model using subjects in the remaining K-1 parts, which is then used to predict new predicted dependent values (Y(p)) of all metabolites for subjects in the Kth partition. Finally, all subjects receive such a set of Y(p) values. New improved estimates of the residual regression variances are then Sum(Y−Y(p))^2^/N for each metabolite, and these are always larger than those estimated from the total sample. Noting that var(beta) for a particular metabolite in the full sample equals residual variance/(N.var(X)), a new somewhat smaller t score (beta/sqrt(Var(beta)) is calculated using the improved estimates of residual variances, and these smaller t-scores are submitted to the FDR procedure. 

For this study, K = 10 partitions were used, and the increase in residual variances, and corresponding changes in t-scores was small. Very few metabolites lost significance as compared to the original results (see Supplementary Materials, Tables S14–S15).

## 3. Results

### 3.1. Baseline Characteristics

Plasma metabolomics profiling was performed on 93 participants—47 vegans and 46 non-vegetarians of the AHS-2 cohort. Roughly equal numbers of male and female and African American and Caucasian participants were included in this study and balanced among the two diet groups. Hence, there were no significant differences at baseline in race or sex comparing these two groups (Table 1). However, vegans were older than non-vegetarians (mean 66.5 vs. 60.8 years), and BMI was significantly higher in non-vegetarians (31.3 vs. 24.7), consistent with greater mean dietary kcals/day. Intakes of select foods or nutrients differed greatly by diet group, as non-vegetarians had significantly higher intakes of red meat, total meat, poultry, fish, dairy, and saturated fat (*p* < 0.001), and vegans had higher intakes of fiber, fruit, vegetables, soy, legumes (*p* < 0.001), and whole grains (*p* < 0.022). Significant differences in all lifestyle factors were seen when comparing vegans with non-vegetarians, most notably coffee drinking, where 39% of non-vegetarians consumed coffee (once or more per month) compared to 0% of vegans (*p* < 0.001). A significantly greater proportion of non-vegetarians also had a history of smoking (*p* = 0.008), alcohol drinking (*p* = 0.015), and used aspirin or non-steroidal anti-inflammatory drugs (NSAIDS) (*p* = 0.038), while the number of minutes of exercise per week for vegans was more than twice as long as non-vegetarians (132 vs. 64 min/wk; *p* = 0.003).

### 3.2. Linear Regression—Abundance of Metabolites or Biochemical Subclasses in Vegans and Non-Vegetarians

#### 3.2.1. Linear Regression to Analyze Abundance of Individual Metabolites

Linear regression of individual metabolites with SmartSVA yielded a total of 586 differential metabolites after adjustment for false discovery. The top 40 metabolites present at higher abundance in vegans relative to non-vegetarians ordered by fold change are shown in Table 2. Fold changes of metabolites comparing vegans with non-vegetarians were derived from geometric mean ratios, based on adjusted means. Differences up to nearly 7-fold were observed. Several top metabolites with fold changes above 2-fold were lipids and xenobiotics. In addition, among metabolites showing greatest abundance in vegans were compounds within amino acid subclasses—including methionine/cysteine metabolism, urea cycle, tryptophan, and tyrosine metabolism, besides lipid subclasses including dicarboxylic acids and bile acid metabolism, among others (Table 2). 

However, the vast majority of differential metabolites differentially abundant at FDR <0.05 (422/586 = 72%) were decreased in vegans. Metabolites showing the most marked decreases in vegans were again prominently xenobiotics, followed by lipids and amino acids (Table 3). This included metabolites from primarily xanthine, histidine, food component, dicarboxylic acid, and drug metabolism, where values were ~3 to ~25-fold higher in the non-vegetarians. Differentially abundant (positively and negatively associated) metabolites belonging to all major classes were identified (Supplementary Materials, Tables S1–S5). 

When surrogate variables were not included in models, a smaller number of metabolites (346) were found to be differentially abundant in vegans relative to non-vegetarians (Supplementary Materials, Tables S6–S11). There was, however, considerable overlap between differentially abundant metabolites (FDR < 0.05) when comparing the regression approaches with and without Smart SVA, as the vast majority of differential metabolites (FDR < 0.05) detected in the regression excluding surrogate variables were identified with inclusion of the SmartSVA approach in the analysis. Metabolites showing greatest fold changes were in strong agreement comparing the two approaches (Table 2 and Table 3, Supplementary Materials Tables S6 and S7). 

#### 3.2.2. Linear Regression to Analyze Metabolite Subclasses

Regression analysis of metabolite subclasses with at least two component metabolites identified 50 differentially abundant subclasses at FDR < 0.05 (Table 4) of 93 total subclasses. Subclasses with increased abundance in vegans prominently included ketone bodies, followed by vitamin A metabolism, inositol, fatty acid acyl glycine metabolism, lactosylceramides, and benzoate metabolism subclasses. For each of these subclasses, all or the majority of the component metabolites significant at FDR < 0.05 were positively associated with a vegan dietary pattern. The directionality of the significant metabolites is notable considering the analysis included all metabolites in a subclass that were represented on the panel. Hence, differential abundance of subclasses, and directionality, were highly driven by component metabolites which had reached statistical significance in linear regression analysis. 

Similar to findings of differential abundance of individual metabolites, the majority of subclasses showing statistical differences between vegans and non-vegetarians were negatively associated with vegans (Table 4). The subclasses with the greatest negative associations in vegans (>1.5-fold change) included xanthine metabolism, drug, branched fatty acid, histidine metabolism, acetylated peptides, ceramides, and dihydroceramides, where all or the vast majority of the statistically significant component metabolites were in decreased abundance. Additionally, all or the vast majority of statistically significant metabolites within many other negatively associated subclasses were also inversely associated with a vegan diet. These included subclasses representing long-chain acyl carnitine metabolism, long-chain saturated fatty acids, lysoplasmalogens, phenylalanines, long-chain monounsaturated fatty acids, monoacylglycerols, and leucine, isoleucine and valine, which are branched chain amino acids (BCAA), among other subclasses. Very similar results were obtained in the analysis excluding surrogate variables, where the top subclasses showing the greatest positive or negative associations with vegans were represented (Supplementary Materials, Table S12) (Names of individual, statistically significant metabolites in each of these differentially abundant subclasses are listed in Supplementary Materials, Table S13).

#### 3.2.3. Cross-Validation and Bootstrapped Regression for Error Analysis 

Linear regression with cross-validation was performed to identify possible metabolites or subclasses falsely rejected as null or non-null. Cross validation yielded results very similar to those obtained with the entire sample, with only 15 metabolites originally found to be differential at FDR < 0.05 not showing significance with cross-validation (type I error) (Supplementary Materials, Table S14), and no metabolites showing significance with cross-validation that were not identified in the analysis of the entire sample (type II error). All 50 subclasses found to be differential with analysis of the entire sample were also significantly differential with cross-validation, with potentially three additional subclasses that were not identified in analysis of the entire sample (Supplementary Materials, Table S15). Bootstrapped regression models also very much coincided with results of the non-bootstrapped regression analysis, with no more than three potentially new, non-null metabolites or subclasses identified (Supplementary Materials, Table S16). The majority of bootstrapped samples showed numbers of differential metabolites or metabolite subclasses comparable to those obtained in the non-bootstrapped analysis, and all metabolites or subclasses that were differential in at least 90% of bootstrapped samples were also differential in the non-bootstrapped regression analysis (Supplementary Materials, Tables S17–S19).

### 3.3. Random Forest and Principal Components Analyses

#### 3.3.1. Random Forest Analysis for Classification by Diet Group

**Figure 1:**
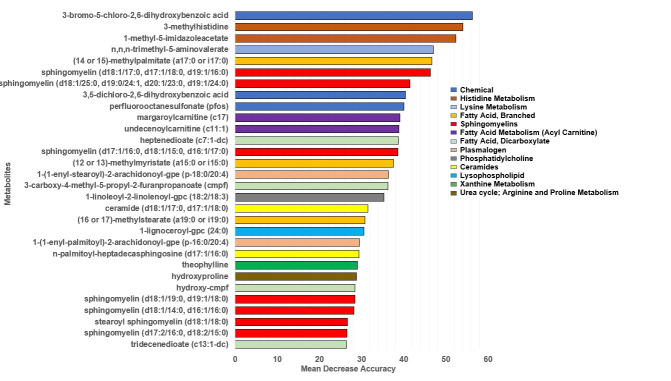
Random forest variable importance. Mean decrease accuracy from random forest analysis classifying vegans and non-vegetarians represents average decrease in accuracy in model prediction after permutation of each indicated variable.

Random forest analysis was used to determine the ability of metabolites to identify the vegan and non-vegetarian dietary classes and to identify metabolites with the greatest predictive accuracy. The highest ranked metabolites with the ability to distinguish vegans from non-vegetarians (with greatest mean decrease accuracy determined by the out-of-bag error/permutation method) included 3-bromo-5-chloro,2-6dihydroxybenzoic acid, 3-methylhistidine, 1-methyl-5-imidazoleacetate, (14 or 15)-methyl palmitate (a17:0 or i17:0), n,n,n-trimethyl-5-aminovalerate, and sphingomyelin (d18:1/17:0, d17:1/18:0, d19:1/16:0) (Figure 1). Classification by diet group showed a predictive accuracy of 92.5%, with a misclassification (out of bag) error of 7.5%. These influential metabolites overlapped with metabolites determined to be highly statistically significant or have large fold changes from the linear regression analysis.

#### 3.3.2. Principal Component Analysis for Dimension Reduction

**Figure 2:**
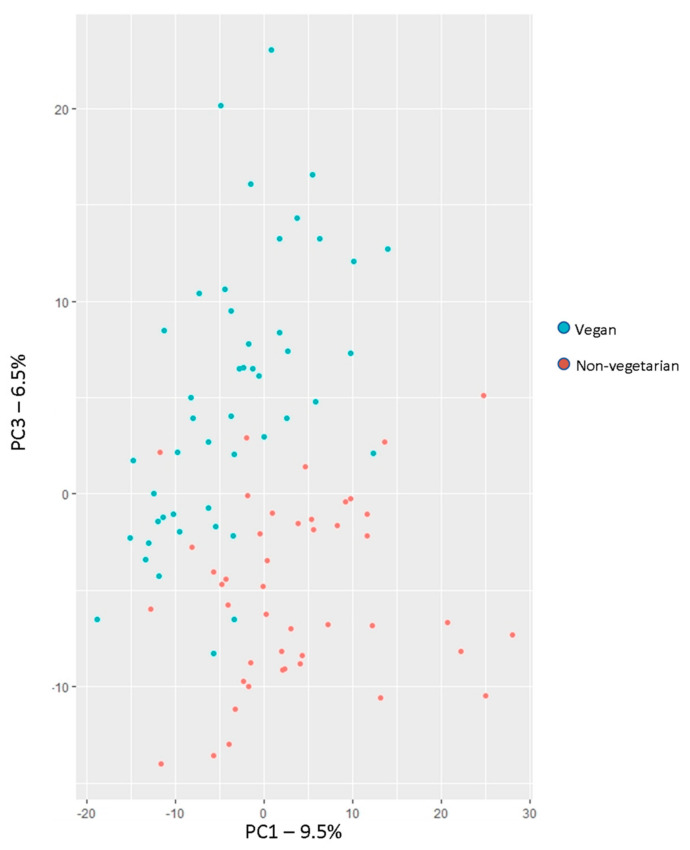
PCA plot of scores for vegan and non-vegetarian participants for principal components 1 and 3. Individual scores were obtained from covariance matrix of 930 log-transformed metabolites and plotted to analyze variation explained by diet group.

Principal component analysis was used to collapse data into orthogonal components and generate clusters of correlated metabolites. Nine hundred thirty metabolites were collapsed into 91 PCs explaining 100% of the variance, for 92 subjects (with exclusion of one outlier). Ten PCs with eigenvalues >20 explained 50% of the variance, with eight of these PCs containing metabolites with loadings >0.5. Metabolites driving PC1 included predominantly long-chain acylcarnitines and ceramides, followed by branched fatty acids and long-chain saturated fatty acids (Supplementary Materials, Figure S2 and Table S20). Most prominent subclasses represented in PC3 included long-chain polyunsaturated fatty acids, and dicarboxylic acids, and there was representation of other types of fatty acids (monohydroxy fatty acids, long-chain monounsaturated fatty acids). Lysophospholipids were the predominant subclass represented in PC4 (Supplementary Materials, Table S20). The top four PCs accounted for nearly 1/3 of the variance, with PC1 and PC3 most clearly separating dietary groups. (Figure 2).

Linear regression was performed to examine associations of each of the 10 PCs with various dietary variables of interest, and correlations determined. Significantly associated PCs (*p* ≤ 0.05) are shown in Table 5. PC1, PC3, and PC4 were associated with a vegan diet (combined r = −0.5, *p* = 7.9 × 10^−7^). These PCs were also associated with consumption of red meat, total meat, processed meat, and poultry, along with fiber and saturated fat, with partial correlations ranging from r = +/− 0.41 to 0.61. The first four PCs were highly correlated with consumption of fish (r = 0.52, *p* = 2.6 × 10^−7^), and dairy kcals (r = 0.61, *p* = 6.4 × 10^−10^). Correlations of red meat, total meat, and processed meat with these PCs were largely attenuated in expanded models including additional dietary covariates. Inclusion of poultry, fish, dairy, and fruit in the model attenuated associations between red meat or total meat and PCs, and an association remained only with PC1 for processed meat. After adjustment for saturated fat and fiber, significant associations remained for red meat and total meat with PC4 (β = 1.7, *p* = 0.009; β = 1.47, *p* = 0.008) (Supplementary Materials, Table S21). 

## 4. Discussion

Among participants in the AHS-2 cohort, distinct metabolic profiles for vegans and non-vegetarians were discovered, with over 60% of metabolites being significantly discriminatory after adjustment for false discovery. Clearly, the serological characteristics of vegans and non-vegetarians differ substantially. The most notable metabolites more abundant in vegans belonged to categories mostly related to plant-food intakes. Those more abundant in non-vegetarians included subclasses of lipids and amino acids, which are mostly related to intakes of animal foods, besides xenobiotics reflecting other lifestyle behaviors such as caffeine consumption and medication use. 

Prominent among metabolites more abundant in vegans were products of benzoate metabolism derived from polyphenols in plant foods, possibly also reflecting gut microbial activity. For example, the top compound, 4-ethylphenyl sulfate, is generated by the metabolism of soy protein by gut bacteria [29,30,31], and other metabolites may be generated through microbial metabolism of dietary polyphenols (hippurate metabolites, catechol sulfate, and others). Higher abundance of other food component/plant metabolites (glucopyranoside metabolites, stachydrine), besides vitamins in vegans, similarly reflects consumption of fruits, vegetables, and herbs [32,33], which have roles in reducing risk of chronic diseases. These findings in vegans are consistent with those obtained by Wu et al., where increases were found in benzoate metabolism products and polyphenolic plant compounds as well as gut microbial metabolites (hippurate, 4-ethylphenyl sulfate, 4-hydoxyhippurate, catechol sulfate, phenol sulfate). Notably, vegans showed a significant increase (30%) in butyrate, a short-chain fatty acid that is generated with increased fermentation of nondigestible carbohydrates or dietary fiber, and has a role in regulating inflammation and epithelial barrier function. 

Other metabolites that were more abundant in vegan serum have relevance to lipid metabolism and metabolic homeostasis. The cause of the observed higher abundance of ketone bodies in vegans is not clear but may be related to the length of the overnight fast, possibly longer in vegans, caloric restriction, or exercise, which was more frequent in vegans. The acyl glycine subclass of lipids also showed higher abundance in vegans. Acyl glycines are metabolites of fatty acids with important roles in lipid signaling, some with anti-inflammatory ability [34]. There is evidence of negative regulation of acyl glycines by branched chain amino acids [35] through lowering of glycine. Hence, the lower abundance of branched chain fatty acids in vegans in our study might explain in part the higher abundance of acyl glycines, and of primary glycine-conjugated bile acids (glycohyocholate, glycochenodeoxycholate, glycohyocholate, glycol-beta-muricholate.). 

The biological and pathophysiologic effects of these differences are several. Carotenoids, associated with vitamin A, and other polyphenols as well as microbial metabolites produced during breakdown of dietary fiber, have anti-inflammatory and antioxidant properties. These compounds counteract oxidative stress and support immune function and gut health to prevent cancer, diabetes, cardiovascular and other diseases. This may happen through inhibition of nuclear factor of activated B-cells (NFkB), regulation of inflammatory cytokines through epigenetic modifications, and increased transcription of antioxidant defense and xenobiotic detoxification genes [36,37,38]. Ketone bodies have beneficial roles in energy metabolism and glucose homeostasis, and thus may prevent or counteract inflammation and oxidative stress [39,40]. Glycine levels have also shown inverse associations with cardiometabolic disease phenotypes [41,42,43,44]. Higher abundance of glycine, along with glycine-conjugated bile acids, has been reported in other cross-sectional studies of vegans and vegetarians [13,45,46,47,48]. 

The vast majority of metabolites showed lower abundance in vegan serum most often reflecting the absence of animal products in the diet, or the much lower intakes of caffeine and use of medications. Many metabolites within the xanthine metabolism subclass, significantly different in vegans and non-vegetarians, are metabolites of caffeine—theophylline, paraxanthine, 5-acetylamino-6-amino-3-methyluracil, and several others, and would reflect the greater coffee consumption (or perhaps medication use) in non-vegetarians. Other xenobiotic metabolites present at significantly lower abundance in vegans included chemical and drug metabolites, which can be explained by the increased use of acetaminophen and other NSAIDs in non-vegetarians. The large (3-fold) decrease in the acetylated peptides (phenylacetylglutamine, phenylacetyl carnitine) in vegans is likely a consequence of metabolism of phenylalanine (converted to phenylacetate), along with glutamine or L-carnitine, which may increase with higher intake of animal meats, protein, or certain pharmaceutics (NSAIDs). More notable was the high abundance of a large number of histidine metabolites and branched chain amino acids—isoleucine, leucine, and valine—in non-vegetarians, likely reflecting dietary consumption of meat and animal products [49,50,51]. 1- and 3-methylhistidine, for example, are biomarkers of skeletal muscle protein breakdown [51,52]. Histidine and other imidazole-containing compounds may also be related to use of pharmaceuticals [49].

The markedly lower abundance of various lipid metabolites in vegans such as ceramides and dihydroceramides, long-chain acylcarnitines, long-chain saturated fatty acids, monoacylglycerols, and branched chain fatty acids may reflect reduced intake of saturated fats and lipids/sphingolipids derived from animal sources in the diet. Only lactosylceramides were increased in vegans, possibly reflecting consumption of the precursor glucosylceramide from dietary plant sources (i.e., soy, wheat) [52].

Our findings are largely consistent with previous studies examining metabolic profiles associated with vegetarian/vegan, and plant-based diets. Decreases in sphingolipids and some acylcarnitines have been observed in vegans relative to omnivores in a cross-sectional study of individuals in the European Prospective Investigation into Cancer and Nutrition (EPIC) cohort following habitual dietary patterns [13]. In another study of individuals following a vegan diet for at least six months, decreases in phospholipids, saturated fatty acids, 3-carboxy-4-methyl-5-propyl-2-furanpropanoic acid (CMPF), and methylhistidine, but increases in plant-derived and microbial metabolites, were observed [14]. Further, the Mediterranean dietary pattern has been associated with alterations in acylcarnitines and phospholipids [10]. Additionally, ceramides, plasmalogens and acylcarnitines were positively associated with a Western dietary pattern in a cross-sectional study examining associations of plasma metabolites with Western or Prudent dietary patterns in the Women’s Health Initiative cohort [8].

There are important biological and pathophysiologic implications of these differences. While histidine may have some beneficial health effects (i.e., antioxidant activity), histidine metabolites and related imidazole derivatives may be associated with impaired insulin signaling, type 2 diabetes, and kidney disease [53,54]. Several cross-sectional studies have shown positive associations of branched chain amino acids with cardiovascular disease risk, metabolic dysregulation, impaired glucose signaling and insulin resistance [55,56,57], and a number of studies have shown positive associations with type 2 diabetes [58,59,60]. Ceramides are critical lipid signaling molecules, and have various and complex biological roles. Both ceramides and sphingolipids may play a role in insulin resistance [61,62,63]. Ceramides have been found to accumulate in tissues of obese individuals, and have been associated with inflammation [64], and also have roles in the regulation of apoptosis and development of neurological disorders [65,66,67]. Lactosylceramides may have a role in promoting innate immunity [68], besides other biological functions. Phenylacetylglutamine, among others of these metabolites, may reflect activity of gut microbiota, and has been associated with cardiovascular disease risk [69,70,71]. Acylcarnitines are formed in the mitochondria during beta oxidation of fatty acids, but increases in plasma may reflect metabolic disorders [72], as these compounds have been associated with insulin resistance, diabetes, and increased cardiovascular disease risk. Similarly, long-chain saturated fatty acids are implicated in increased risk of obesity and cardiometabolic diseases [73]. Mono-acylglycerols are converted to triacylglycerols, which are associated with heart disease, obesity, and metabolic syndrome [74]. Branched chain fatty acids, on the other hand, which are derived largely from dairy and meat products (though also synthesized by gut bacteria from branched chain amino acids in herbivores [75]), may favorably influence metabolic health. Accordingly, they have potentially beneficial effects on insulin sensitivity and weight management, and may attenuate inflammation [76,77,78], but further human studies are needed.

Vegetarians and particularly vegans have notably higher consumption of fruits, vegetables, legumes, whole grains, soy foods, and nuts, and markedly reduced or absent intake of animal products, as demonstrated previously in the AHS-2 cohort [25]. Additionally, compared to non-vegetarians, they have more favorable fatty acid profiles, including lower saturated fatty acids, and higher total omega-3, along with higher levels of phytochemicals such as carotenoids, enterolactone, and isoflavones in plasma or urine [15], but lower inflammatory cytokines [79]. Importantly, vegetarians have shown significantly reduced risks of diabetes, hypertension, cardiovascular disease, select cancers, and all-cause mortality relative to non-vegetarians [1,2,3,80]. These differences in disease and biomarker profiles between vegans and non-vegetarians coincide with the differences in plasma metabolites identified in the present study.

Strengths of the current study include well-defined diet groups (vegans and non-vegetarians) reflecting habitual dietary patterns [21], and the inclusion of higher meat consuming non-vegetarians. An additional strength is the analytical rigor applied with the use of multiple regression and other approaches, including adjustment for surrogate variables to address additional confounding and unwanted variation, besides classification and dimensionality reduction approaches. Further, adjustment for false discovery with use of the adapted Storey et al. [26] method allowed for an accurate estimation of the proportion of t-statistics from a non-null distribution, which provides an improvement in power compared to other approaches. AHS-2 participants have provided extensive dietary data, besides demographic and medical data, strengthening the analysis of the current study. Limitations of the study are the single measurement of plasma metabolites, and the somewhat limited sample size, although there was sufficient power to detect a large number of statistically significant differences between vegans and non-vegetarians. Estimates of results from an independent sample are obtained by cross-validation. There are notable lifestyle differences in this Adventist cohort compared to other vegan and non-vegetarian individuals. Seventh-day Adventists place an emphasis on health and wellness, and there are religious guidelines prohibiting certain lifestyle behaviors (smoking, alcohol drinking, biblically unclean meats), but no prohibitions for clean meats in general or dairy. These healthy practices, which limit confounding by these factors, may possibly also limit generalizability of results. The overall reduced consumption of non-fish, and particularly red meats among AHS-2 non-vegetarians relative to the general population, was a limitation, although this was partially overcome by selection of subjects with higher meat consumption. As many diet-derived metabolites are converted by gut bacteria, it remains to be understood how differences in these metabolites might be linked with alterations in composition of the gut microbiome.

## 5. Conclusions

In conclusion, in this study we report marked differences in metabolic profiles between vegans and non-vegetarians. Our results suggest that multiple potentially bioactive metabolites are increased by consumption of plant-based foods, and may lower the risk of metabolic diseases through anti-inflammatory mechanisms. On the other hand, diets high in animal products may lead to increases in various amino acids and lipid species (acyl carnitines, saturated fatty acids, ceramides, branched chain amino acids) that promote chronic diseases by increasing inflammation and insulin dysregulation, so disrupting metabolic homeostasis. The exact roles or physiological functions of other differentially abundant metabolites are not clear. It may be that some differentially abundant metabolites in vegans and non-vegetarians serve only as markers of different foods or eating patterns, while others also have important pathophysiological consequences. This study helps lay the foundation for a deeper understanding of the relationship of diet-associated metabolites to the pathophysiology of chronic diseases.

## Figures and Tables

**Table 1:** Demographic, dietary, and lifestyle characteristics of study population ^1,2^.

	Non-Vegetarian	Vegan	*p*-Value
Participants, *n*	46	47	
Demographic			
Sex			1
Male	23 (50)	24 (51)	
Female	23 (50)	23 (49)	
Age (years)	60.8 (11.1)	66.5 (9.9)	0.011
BMI (kg/m^2^) ^3^	31.3 (7.0)	24.7 (3.4)	<0.001
Ethnicity			1
Caucasian	23 (50)	23 (49)	
Black	23 (50)	24 (51)	
Dietary			
Total kca^3^	2520.8 (902.8)	1776.8 (744.7)	<0.001
Fiber (g/d)	19.6 (6.5)	40.5 (8.8)	<0.001
Saturated fat (g/d)	21.8 (7.9)	9.0 (2.7)	<0.001
Total meat (g/d)	104.9 (41.0)	0	< 0.001
Red meat (g/d)	54.9 (22.8)	0	< 0.001
Processed meat (g/d)	6.4 (6.6)	0	<0.001
Poultry (g/d)	31.2 (21.6)	0	< 0.001
Fish (g/d)	20.7 (17.2)	0	< 0.001
Dairy (kcal/d)	252.8 (151.7)	17.0 (14.7) ^3^	<0.001
Fruit (kcal/d)	108.3 (64.9)	320.9 (159.3)	<0.001
Vegetables (kcal/d)	67.6 (36.3)	134.5 (115.4)	<0.001
Soy (kcal/d)	60.06 (42.3)	156.3 (99.2)	<0.001
Legumes (kcal/d)	46.1 (56.4)	73.2 (56.0)	<0.001
Whole grains (kcal/d)	172.4 (130.9)	326.6 (189.8)	<0.022
Lifestyle			
Exercise (min/week) ^4^	63.6 (100.8)	132.3 (110.6)	0.003
Smoking (ever vs. never)	12 (26.1)	2 (4.3)	0.008
Alcohol drinking (ever vs. never)	24 (52.2)	12 (25.5)	0.015
Coffee (any vs. never/rarely)	18 (39.1)	0	<0.001
Aspirin/NSAIDS (<1/wk vs. ≥1/wk)	11 (23.9)	3 (6.4)	0.038

^1^ Values presented as n (%) or mean (SD). ^2^ Dietary variables adjusted for kcal/day. ^3^ Based on follow up phone calls in a sample, these small quantities of dairy are most likely artefactual coming from recipes of certain mixed foods where vegans can choose non-dairy options not reflected in these recipes. ^4^ Missing values: body mass index (BMI), *n* = 2; kcal, *n* = 1; Exercise, *n* = 2. NSAIDS = non-steroidal anti-inflammatory drugs.

**Table 2:** Top 40 (of 164) metabolites positively associated with a vegan relative to non-vegetarian dietary pattern at FDR < 0.05 ^1,2^.

Metabolite	Fold Change	FDR	Subclass	Major Class
4-ethylphenyl sulfate	6.58	5.2 × 10^−4^	Benzoate Metabolism	Xenobiotics
s-methylmethionine	4.61	2.0 × 10^−4^	Methionine, Cysteine, SAM, and Taurine Metabolism	Amino Acid
branched chain 14:0 dicarboxylic acid	3.54	2.2 × 10^−4^	Fatty Acid, Dicarboxylate	Lipid
4-acetylphenyl sulfate	3.20	2.6 × 10^−4^	Benzoate Metabolism	Xenobiotics
glycohyocholate	2.86	6.3 × 10^−4^	Secondary Bile Acid Metabolism	Lipid
ethyl beta-glucopyranoside	2.79	1.5 × 10^−4^	Food Component/Plant	Xenobiotics
methyl glucopyranoside (alpha + beta)	2.64	2.9 × 10^−4^	Food Component/Plant	Xenobiotics
beta-cryptoxanthin	2.52	1.0 × 10^−3^	Vitamin A Metabolism	Cofactors and Vitamins
n-methylproline	2.28	9.0 × 10^−5^	Urea cycle; Arginine and Proline Metabolism	Amino Acid
stachydrine	2.27	1.6 × 10^−4^	Food Component/Plant	Xenobiotics
4-allylcatechol sulfate	2.21	6.7 × 10^−5^	Benzoate Metabolism	Xenobiotics
indolepropionate	2.03	9.8 × 10^−5^	Tryptophan Metabolism	Amino Acid
2-methylserine	1.95	1.3 × 10^−4^	Glycine, Serine, and Threonine Metabolism	Amino Acid
1-linoleoyl-2-linolenoyl-gpc (18:2/18:3)	1.93	4.5 × 10^−4^	Phosphatidylcholine (PC)	Lipid
s-methylcysteine sulfoxide	1.93	1.1 × 10^−4^	Methionine, Cysteine, SAM, and Taurine Metabolism	Amino Acid
daidzein sulfate (2)	1.91	9.8 × 10^−3^	Food Component/Plant	Xenobiotics
gentisate	1.90	7.8 × 10^−5^	Tyrosine Metabolism	Amino Acid
4-allylphenol sulfate	1.89	2.4 × 10^−3^	Food Component/Plant	Xenobiotics
dodecanedioate (c12:1-dc)	1.88	1.8 × 10^−4^	Fatty Acid, Dicarboxylate	Lipid
glycochenodeoxycholate 3-sulfate	1.87	2.3 × 10^−3^	Primary Bile Acid Metabolism	Lipid
3-hydroxybutyrate (bhba)	1.86	1.7 × 10^−4^	Ketone Bodies	Lipid
octadecanedioate (c18:1-dc)	1.83	3.5 × 10^−4^	Fatty Acid, Dicarboxylate	Lipid
cinnamoylglycine	1.82	1.7 × 10^−3^	Food Component/Plant	Xenobiotics
n-delta-acetylornithine	1.79	1.6 × 10^−4^	Urea cycle; Arginine and Proline Metabolism	Amino Acid
3-hydroxydodecanedioate	1.76	3.9 × 10^−4^	Fatty Acid, Dicarboxylate	Lipid
catechol sulfate	1.75	1.2 × 10^−4^	Benzoate Metabolism	Xenobiotics
octadecadienedioate (c18:2-dc)	1.75	1.2 × 10^−4^	Fatty Acid, Dicarboxylate	Lipid
tryptophan betaine	1.74	1.8 × 10^−3^	Tryptophan Metabolism	Amino Acid
n-linoleoylglycine	1.70	6.2 × 10^−5^	Fatty Acid Metabolism (Acyl Glycine)	Lipid
4-methoxyphenol sulfate	1.69	1.4 × 10^−3^	Tyrosine Metabolism	Amino Acid
octadecenedioylcarnitine (c18:1-dc)	1.69	1.4 × 10^−4^	Fatty Acid Metabolism (Acyl Carnitine, Dicarboxylate)	Lipid
4-ethylcatechol sulfate	1.68	3.5 × 10^−3^	Benzoate Metabolism	Xenobiotics
12,13-dihome	1.66	9.5 × 10^−5^	Fatty Acid, Dihydroxy	Lipid
carotene diol (2)	1.65	8.0 × 10^−5^	Vitamin A Metabolism	Cofactors and Vitamins
chiro-inositol	1.65	1.2 × 10^−3^	Inositol Metabolism	Lipid
2-acetamidophenol sulfate	1.65	2.1 × 10^−2^	Food Component/Plant	Xenobiotics
3-hydroxysebacate	1.63	2.1 × 10^−4^	Fatty Acid, Monohydroxy	Lipid
acetoacetate	1.63	1.6 × 10^−4^	Ketone Bodies	Lipid
Cis-4-decenoate (10:1n6)	1.63	1.2 × 10^−4^	Medium Chain Fatty Acid	Lipid
pentose acid	1.62	7.7 × 10^−5^	Partially Characterized Molecules	Partially Characterized Molecules

^1^ Fold change represents ratio of geometric means of vegans relative to non-vegetarians obtained from linear regression model with SmartSVA. ^2^ Adapted Storey et al. [26] permutation approach used to adjust for false discovery.

**Table 3:** Top 40 (of 422) metabolites inversely associated with a vegan relative to non-vegetarian dietary pattern at FDR < 3.9 × 10^−5 1,2^.

Metabolite	Fold Change	Subclass	Major Class
5-acetylamino-6-amino-3-methyluracil	0.04	Xanthine Metabolism	Xenobiotics
theobromine	0.04	Xanthine Metabolism	Xenobiotics
3-carboxy-4-methyl-5-propyl-2-furanpropanoate (cmpf)	0.05	Fatty Acid, Dicarboxylate	Lipid
paraxanthine	0.07	Xanthine Metabolism	Xenobiotics
theophylline	0.07	Xanthine Metabolism	Xenobiotics
3-methylhistidine	0.07	Histidine Metabolism	Amino Acid
1-methyl-5-imidazoleacetate	0.09	Histidine Metabolism	Amino Acid
hydroxy-cmpf	0.09	Fatty Acid, Dicarboxylate	Lipid
1,7-dimethylurate	0.09	Xanthine Metabolism	Xenobiotics
4-acetaminophen sulfate	0.10	Drug—Analgesics, Anesthetics	Xenobiotics
caffeine	0.11	Xanthine Metabolism	Xenobiotics
piperine	0.12	Food Component/Plant	Xenobiotics
7-methylxanthine	0.17	Xanthine Metabolism	Xenobiotics
2-hydroxyacetaminophen sulfate	0.18	Drug—Analgesics, Anesthetics	Xenobiotics
3-methylxanthine	0.19	Xanthine Metabolism	Xenobiotics
4-acetamidophenol	0.19	Drug—Analgesics, Anesthetics	Xenobiotics
sulfate of piperine metabolite c16h19no3 (2)	0.19	Food Component/Plant	Xenobiotics
1-methylurate	0.20	Xanthine Metabolism	Xenobiotics
5-acetylamino-6-formylamino-3-methyluracil	0.20	Xanthine Metabolism	Xenobiotics
3-bromo-5-chloro-2,6-dihydroxybenzoic acid	0.22	Chemical	Xenobiotics
sulfate of piperine metabolite c16h19no3 (3)	0.23	Food Component/Plant	Xenobiotics
n,n,n-trimethyl-5-aminovalerate	0.24	Lysine Metabolism	Amino Acid
glucuronide of piperine metabolite c17h21no3 (4)	0.25	Food Component/Plant	Xenobiotics
sulfate of piperine metabolite c18h21no3 (1)	0.26	Food Component/Plant	Xenobiotics
heptenedioate (c7:1-dc)	0.27	Fatty Acid, Dicarboxylate	Lipid
glucuronide of piperine metabolite c17h21no3 (3)	0.27	Food Component/Plant	Xenobiotics
glucuronide of piperine metabolite c17h21no3 (5)	0.28	Food Component/Plant	Xenobiotics
1-methylxanthine	0.29	Xanthine Metabolism	Xenobiotics
(14 or 15)-methyl palmitate (a17:0 or i17:0)	0.29	Fatty Acid, Branched	Lipid
sulfate of piperine metabolite c18h21no3 (3)	0.29	Food Component/Plant	Xenobiotics
ibuprofen	0.29	Drug—Analgesics, Anesthetics	Xenobiotics
(12 or 13)-methyl myristate (a15:0 or i15:0)	0.30	Fatty Acid, Branched	Lipid
1,3-dimethylurate	0.32	Xanthine Metabolism	Xenobiotics
3-methyl catechol sulfate (1)	0.32	Benzoate Metabolism	Xenobiotics
1-margaroylglycerol (17:0)	0.32	Monoacylglycerol	Lipid
perfluorooctanesulfonate (pfos)	0.33	Chemical	Xenobiotics
saccharin	0.35	Food Component/Plant	Xenobiotics
tridecenedioate (c13:1-dc)	0.36	Fatty Acid, Dicarboxylate	Lipid
3,5-dichloro-2,6-dihydroxybenzoic acid	0.38	Chemical	Xenobiotics
sphingomyelin (d18:1/25:0, d19:0/24:1, d20:1/23:0, d19:1/24:0)	0.39	Sphingomyelins	Lipid

^1^ Fold change represents ratio of geometric means of vegans relative to non-vegetarians obtained from linear regression model with SmartSVA. ^2^ Adapted Storey et al. [26] permutation approach used to adjust for false discovery.

**Table 4:** Metabolite subclasses associated with diet group (vegan vs. non-vegetarian) at FDR < 0.05 ^1^.

Subclass	Fold Change (95% CI)	FDR ^2^	Significant Metabolites (*n*)	#↓ ^3^	#↑ ^4^	*n* Total Metabolites ^5^
Ketone Bodies	1.75 (2.16, 1.43)	1.0 × 10^−3^	2	0	2	2
Vitamin A Metabolism	1.40 (1.57, 1.25)	2.0 × 10^−3^	6	2	4	6
Inositol Metabolism	1.35 (1.57, 1.16)	6.0 × 10^−3^	2	0	2	2
Fatty Acid Metabolism (Acyl Glycine)	1.21 (1.35, 1.08)	2.6 × 10^−2^	5	1	4	7
Lactosylceramides (LCER)	1.17 (1.28, 1.07)	2.8 × 10^−2^	2	0	2	3
Benzoate Metabolism	1.16 (1.27, 1.06)	4.8 × 10^−2^	16	7	9	24
Amino Sugar Metabolism	0.94 (1.00, 0.88)	4.7 × 10^−2^	2	2	0	5
Urea cycle; Arginine and Proline Metabolism	0.94 (0.99, 0.90)	2.3 × 10^−2^	14	10	4	21
Glutamate Metabolism	0.94 (0.99, 0.89)	3.4 × 10^−2^	8	5	3	12
Tyrosine Metabolism	0.92 (0.98, 0.86)	1.1 × 10^−2^	11	9	2	22
Pyrimidine Metabolism, Uracil containing	0.92 (0.97, 0.88)	1.1 × 10^−3^	5	5	0	12
Purine Metabolism, Adenine containing	0.92 (0.97, 0.88)	1.4 × 10^−3^	3	3	0	6
Fatty Acid, Dicarboxylate	0.91 (0.98, 0.85)	1.5 × 10^−2^	22	10	12	34
Long-Chain Polyunsaturated Fatty Acid (n3 and n6)	0.90 (0.97, 0.83)	1.0 × 10^−2^	12	9	3	17
Phospholipid Metabolism	0.90 (0.96, 0.85)	1.2 × 10^−3^	4	4	0	7
Sphingolipid Synthesis	0.89 (0.98, 0.80)	2.1 × 10^−2^	2	2	0	3
Alanine and Aspartate Metabolism	0.89 (0.94, 0.85)	1.1 × 10^−4^	6	5	1	9
Partially Characterized Molecules	0.88 (0.98, 0.79)	2.8 × 10^−2^	8	6	2	13
Creatine Metabolism	0.86 (0.92, 0.79)	1.7 × 10^−4^	3	2	1	3
Diacylglycerol	0.84 (0.97, 0.72)	2.5 × 10^−2^	6	6	0	14
Pyrimidine Metabolism, Orotate containing	0.84 (0.93, 0.76)	9.9 × 10^−4^	3	3	0	4
Purine Metabolism, (Hypo)Xanthine/Inosine containing	0.84 (0.90, 0.77)	<3.9 × 10^−5^	5	5	0	7
Fructose, Mannose, and Galactose Metabolism	0.83 (0.98, 0.70)	3.4 × 10^−2^	2	2	0	4
Secondary Bile Acid Metabolism	0.83 (0.95, 0.72)	8.1 × 10^−3^	11	9	2	21
Purine Metabolism, Guanine containing	0.83 (0.90, 0.76)	<3.9 × 10^−5^	3	3	0	3
Sphingomyelins	0.83 (0.89, 0.77)	<3.9 × 10^−5^	22	18	4	29
Food Component/Plant	0.83 (0.88, 0.78)	<3.9 × 10^−5^	36	22	14	51
Fatty Acid Metabolism (also BCAA Metabolism)	0.82 (0.93, 0.72)	4.8 × 10^−3^	4	4	0	5
Tryptophan Metabolism	0.82 (0.88, 0.76)	<3.9 × 10^−5^	17	14	3	20
Pantothenate and CoA Metabolism	0.81 (0.97, 0.68)	2.6 × 10^−2^	1	1	0	2
Guanidino and Acetamido Metabolism	0.80 (0.94, 0.69)	1.0 × 10^−2^	2	2	0	2
Lysine Metabolism	0.80 (0.87, 0.74)	<3.9 × 10^−5^	11	11	0	18
Plasmalogen	0.80 (0.85, 0.75)	<3.9 × 10^−5^	7	6	1	11
Glycerolipid Metabolism	0.79 (0.88, 0.71)	4.3 × 10^−5^	3	3	0	3
Leucine, Isoleucine, and Valine Metabolism	0.79 (0.84, 0.75)	<3.9 × 10^−5^	24	23	1	32
Dihydrosphingomyelins	0.78 (0.89, 0.69)	3.0 × 10^−4^	5	4	1	5
Chemical	0.78 (0.83, 0.73)	<3.9 × 10^−5^	13	10	3	20
Monoacylglycerol	0.77 (0.84, 0.69)	<3.9 × 10^−5^	11	11	0	17
Lysoplasmalogen	0.75 (0.82, 0.68)	<3.9 × 10^−5^	4	4	0	4
Phenylalanine Metabolism	0.75 (0.80, 0.70)	<3.9 × 10^−5^	6	6	0	7
Long-Chain Monounsaturated Fatty Acid	0.74 (0.80, 0.68)	<3.9 × 10^−5^	4	4	0	7
Long-Chain Saturated Fatty Acid	0.73 (0.78, 0.68)	<3.9 × 10^−5^	7	7	0	8
Fatty Acid Metabolism (Acyl Carnitine, Long-Chain Saturated)	0.72 (0.80, 0.65)	<3.9 × 10^−5^	8	8	0	8
Ceramides	0.64 (0.72, 0.57)	<3.9 × 10^−5^	10	10	0	11
Dihydroceramides	0.62 (0.74, 0.52)	<3.9 × 10^−5^	2	2	0	2
Acetylated Peptides	0.59 (0.74, 0.48)	<3.9 × 10^−5^	4	4	0	4
Histidine Metabolism	0.59 (0.65, 0.55)	<3.9 × 10^−5^	11	10	1	15
Fatty Acid, Branched	0.33 (0.38, 0.29)	<3.9 × 10^−5^	3	3	0	3
Drug—Analgesics, Anesthetics	0.22 (0.29, 0.16)	<3.9 × 10^−5^	5	5	0	5
Xanthine Metabolism	0.14 (0.20, 0.10)	<3.9 × 10^−5^	13	13	0	13

^1^ Linear regression analysis with SmartSVA based on composite t-statistics generated by dividing standard deviation of averaged numerators representing log transformed metabolites. ^2^ Adapted Storey et al. [26] permutation approach used to adjust for false discovery. ^3^ Number of metabolites within subclass that were inversely associated with a vegan diet among those significantly differential in the linear regression analysis. ^4^ Number of metabolites within subclass that were positively associated with a vegan diet among those significantly differential in the linear regression analysis. ^5^ Total number of metabolites measured.

**Table 5:** Adjusted linear regression predicting dietary and lifestyle characteristics from top principal components derived from principal component analysis ^1^.

	β	SE	T Value	*p*-Value	Correlation Coefficient	*p*-Value (Correlation)
Vegan					−0.5	7.9 × 10^−7^
PC1	−7.33	1.96	−3.7	3.3 × 10^−4^		
PC3	8.52	1.88	4.6	1.7 × 10^−5^		
PC4	−5.23	1.44	−3.6	5.0 × 10^−4^		
BMI					0.61	2.1 × 10^−10^
PC1	0.99	0.15	6.5	4.7 × 10^−9^		
PC2	−0.28	0.13	−2.1	4.0 × 10^−2^		
PC3	−0.35	0.15	−2.4	2.0 × 10^−2^		
Red meat					0.52	3.5 × 10^−7^
PC1	1.89	0.49	3.9	3.7 × 10^−3^		
PC3	−2.14	0.47	−4.6	1.7 × 10^−5^		
PC4	1.35	0.36	3.7	3.5 × 10^−4^		
Total meat					0.51	4.0 × 10^−7^
PC1	1.61	0.42	3.8	2.7 × 10^−4^		
PC3	−1.86	0.4	−4.6	1.4 × 10^−5^		
PC4	1.17	0.31	3.8	3.2 × 10^−4^		
Processed meat						
PC1	1.51	0.37	4.1	8.9 × 10^−5^	0.44	2.04 × 10^−5^
PC3	−0.79	0.39	−2.1	4.0 × 10^−2^		
PC4	0.75	0.28	2.7	9.6 × 10^−3^		
Poultry						
PC1	1.99	0.57	3.49	7.6 × 10^−4^	0.45	1.10 x 10^−5^
PC3	−2.25	0.55	−4.1	9.2 × 10^−5^		
PC4	1.45	0.42	3.46	8.5 × 10^−4^		
Fish					0.52	2.6 × 10^−7^
PC1	1.91	0.61	3.1	2.0 × 10^−3^		
PC2	−1.15	0.56	−2.1	4.0 × 10^−2^		
PC3	−2.27	0.58	−3.9	2.0 × 10^−4^		
PC4	1.64	0.44	3.8	3.0 × 10^−4^		
Fiber					−0.41	7.2 × 10^−5^
PC1	−6.67	2.18	−3.1	2.9 × 10^−3^		
PC3	9.13	2.04	4.5	2.2 × 10^−5^		
PC4	−3.32	1.65	−2.0	5.0 × 10^−2^		
Soy					−0.22	0.04
PC4	−1.7	0.72	−2.4	2.0 × 10^−2^		
PC7	1.21	0.53	2.3	2.5 × 10^−2^		
Vegetables					−0.23	0.03
PC1	−2.7	1.18	−2.3	2.5 × 10^−2^		
PC8	−2.4	0.65	−3.7	4.3 × 10^−4^		
Fruit						
PC1	−4.69	0.94	−5.0	3.4 × 10^−6^	−0.47	3.4 × 10^−6^
Dairy					0.63	9.6 × 10^−11^
PC1	2.27	0.56	4.1	9.7 × 10^−5^		
PC2	−1.03	0.53	−2.0	5.4 × 10^−2^		
PC3	−2.06	0.56	−3.7	3.8 × 10^−4^		
PC4	1.26	0.42	3.0	3.9 × 10^−3^		
						
Saturated fat						
PC1	7.44	1.63	4.6	1.7 × 10^−5^	0.55	3.1 × 10^−8^
PC3	−6.7	1.64	−4.1	9.3 × 10^−5^		
PC4	3.24	1.29	2.5	1.4 × 10^−2^		
						
Whole grains						
PC3	3.14	0.89	3.5	6.6 × 10^−4^	0.36	6.6 × 10^−4^

^1^ Individual principal components (PCs) regressed on indicated dietary variables in linear regression model adjusted for age, sex, race, and BMI.

## Data Availability

Data supporting the results of this study will be available upon request, once reviewed and approved by the authors.

## Supplementary Materials

The following supporting information can be downloaded at: https://www.mdpi.com/article/10.3390/nu14030709/s1; Table S1. Amino acid metabolites associated with a vegan (relative to non-vegetarian) dietary pattern at FDR < 0.05 in linear regression models with SmartSVA approach; Table S2: Lipid metabolites associated with a vegan (relative to non-vegetarian) dietary pattern at FDR < 0.05 in linear regression models with SmartSVA approach; Table S3: Carbohydrate, cofactor/vitamin, and energy metabolites associated with a vegan (relative to non-vegetarian) dietary pattern at FDR < 0.05 in linear regression models with SmartSVA approach; Table S4: Nucleotides, partially characterized molecules, and peptides associated with a vegan (relative to non-vegetarian) dietary pattern at FDR < 0.05 in linear regression models with SmartSVA approach; Table S5: Xenobiotic metabolites associated with a vegan (relative to non-vegetarian) dietary pattern at FDR < 0.05 in linear regression models with SmartSVA approach; Table S6: Top 40 metabolites positively associated with a vegan dietary pattern at FDR < 0.05 in linear regression analysis without SmartSVA; Table S7: Top 40 metabolites inversely associated with a vegan dietary pattern at FDR < 0.05 in linear regression analysis without SmartSVA; Table S8: Amino acid metabolites associated with a vegan (relative to non-vegetarian) dietary pattern at FDR < 0.05 in linear regression analysis without SmartSVA; Table S9: Lipid metabolites associated with a vegan (relative to non-vegetarian) dietary pattern at FDR < 0.05 in linear regression analysis without SmartSVA; Table S10: Carbohydrate, cofactors/vitamins, energy, nucleotide, partially characterized, and peptide metabolites associated with a vegan dietary pattern in linear regression analysis without SmartSVA; Table S11: Xenobiotic metabolites associated with a vegan (relative to non-vegetarian) dietary pattern at FDR < 0.05 in linear regression analysis without SmartSVA; Table S12: Metabolite subclasses associated with diet group (vegan vs. non-vegetarian) at FDR < 0.05 without SmartSVA; Table S13: Component metabolites of each subclass associated with a vegan (relative to non-vegetarian dietary pattern at FDR < 0.05; Table S14: Metabolites differentially abundant (FDR < 0.05) in linear regression analysis with full sample that were nondifferential post cross-validation; Table S15: Subclasses differential after cross-validation that were not differential in regression analysis with entire sample; Table S16: Metabolites or metabolite subclasses showing differential abundance between vegans and non-vegetarians (FDR < 0.05) in > 50% of bootstrapped linear regression analyses, but not differential in non-bootstrapped regression analysis; Table S17: Numbers of differential metabolites or metabolite subclasses (at FDR < 0.05) in regression analysis with bootstrap sampling; Table S18: List of 129 metabolites showing differential abundance (FDR < 0.05) in at least 90% of bootstrap regressions; Table S19: Metabolite subclasses showing differential abundance (FDR < 0.05) in at least 90% of bootstrap regressions; Table S20: Top components from principal components analysis and most influential metabolites; Table S21:Adjusted linear regression predicting red meat, processed, and total meat consumption from top principal com-ponents from principal components analysis, with adjustment for additional potential dietary confounders; Figure S1. Study design for individuals in the Metabolomics Pilot Study. Footnotes: (a) The Calibration sub-study was a random sample of the cohort, except for an overweighting of Black subjects so they formed 40% of the total. (b) The two pilot sub-studies were convenience samples of study subjects living in Texas (Black subjects) or Washington State designed to test our bio-sample acquisition strategies. (c) The BioMRS sub-study was a local sample of AHS-2 subjects who had responded to a request to complete a questionnaire containing psychosocial and religiosity questions. They lived within 50 miles of Loma Linda, Riverside, or downtown Los Angeles and were at least 50 years of age, Figure S2. Subclass loadings from first principal component (PC1). Principal component analysis was used to identify components explaining variation in metabolites comparing vegans and non-vegetarians. Metabolite loadings > 0.5 were extracted and averaged across each represented subclass.
