# Supplementary material for: The Biology of Veganism: Plasma Metabolomics Analysis Reveals Distinct Profiles of Vegans and Non-Vegetarians in the Adventist Health Study-2 Cohort
Source: Nutrients. 2022 Feb 8;14(3):709. doi: 10.3390/nu14030709 (PMC8839915; doi:10.3390/nu14030709)
Supplement: Supplementary file 1 [file nutrients-14-00709-s001.zip › nutrients-1568819-supplementary.pdf]

Table S1. Amino acid metabolites associated with a vegan (relative to non-vegetarian) dietary pattern at FDR < 0.05 in linear regression models with SmartSVA approach.

Metabolite	Fold Change	FDR	Subclass
2-hydroxy-4-(methylthio)butanoic acid	1.57	<3.9E-05	Methionine, Cysteine, SAM and Taurine Metabolism
kynurenate	1.32	<3.9E-05	Tryptophan Metabolism
indoleacetylglutamine	2.31	<3.9E-05	Tryptophan Metabolism
2-oxoarginine	1.86	<3.9E-05	Urea cycle; Arginine and Proline Metabolism
phenylalanine	1.11	<3.9E-05	Phenylalanine Metabolism
isovalerylcarnitine (c5)	1.57	<3.9E-05	Leucine, Isoleucine and Valine Metabolism
3-hydroxy-2-ethylpropionate	1.41	<3.9E-05	Leucine, Isoleucine and Valine Metabolism
isovalerylglycine	1.65	<3.9E-05	Leucine, Isoleucine and Valine Metabolism
n-acetylphenylalanine	1.45	<3.9E-05	Phenylalanine Metabolism
n-formylanthranilic acid	1.77	<3.9E-05	Tryptophan Metabolism
n-acetyltyrosine	1.61	<3.9E-05	Tyrosine Metabolism
indole-3-carboxylate	1.59	<3.9E-05	Tryptophan Metabolism
2-hydroxyphenylacetate	1.48	<3.9E-05	Phenylalanine Metabolism
anthranilate	1.69	<3.9E-05	Tryptophan Metabolism
glutamate	1.41	<3.9E-05	Glutamate Metabolism
xanthurenate	2.01	<3.9E-05	Tryptophan Metabolism
valine	1.30	<3.9E-05	Leucine, Isoleucine and Valine Metabolism
1-carboxyethylleucine	1.69	<3.9E-05	Leucine, Isoleucine and Valine Metabolism
6-oxopiperidine-2-carboxylate	1.87	<3.9E-05	Lysine Metabolism
2,3-dihydroxy-5-methylthio-4-pentenoate (dmtpa)	1.21	<3.9E-05	Methionine, Cysteine, SAM and Taurine Metabolism
2-methylbutyrylcarnitine (c5)	1.71	<3.9E-05	Leucine, Isoleucine and Valine Metabolism
1-carboxyethyltyrosine	1.80	<3.9E-05	Tyrosine Metabolism
creatine	1.68	<3.9E-05	Creatine Metabolism
homocitrulline	1.80	<3.9E-05	Urea cycle; Arginine and Proline Metabolism
tiglyl carnitine (c5)	1.65	<3.9E-05	Leucine, Isoleucine and Valine Metabolism
beta-hydroxyisovaleroylcarnitine	1.67	<3.9E-05	Leucine, Isoleucine and Valine Metabolism
1-carboxyethylvaline	1.66	<3.9E-05	Leucine, Isoleucine and Valine Metabolism
urea	1.38	<3.9E-05	Urea cycle; Arginine and Proline Metabolism
methionine sulfoxide	1.34	<3.9E-05	Methionine, Cysteine, SAM and Taurine Metabolism
n-acetylvaline	1.27	<3.9E-05	Leucine, Isoleucine and Valine Metabolism
formiminoglutamate	1.84	<3.9E-05	Histidine Metabolism
1-carboxyethylphenylalanine	1.54	<3.9E-05	Phenylalanine Metabolism
1-carboxyethylisoleucine	1.77	<3.9E-05	Leucine, Isoleucine and Valine Metabolism
hydroxyproline	1.48	<3.9E-05	Urea cycle; Arginine and Proline Metabolism
n6-acetyllysine	1.41	<3.9E-05	Lysine Metabolism
1-methylhistidine	1.66	<3.9E-05	Histidine Metabolism
2-aminoadipate	1.84	<3.9E-05	Lysine Metabolism
n,n,n-trimethyl-5-aminovalerate	4.16	<3.9E-05	Lysine Metabolism
3-methylhistidine	14.99	<3.9E-05	Histidine Metabolism
1-methyl-5-imidazoleacetate	11.46	<3.9E-05	Histidine Metabolism
phenyllactate (pla)	1.26	5.6E-05	Phenylalanine Metabolism
lysine	1.14	5.8E-05	Lysine Metabolism
asparagine	0.86	6.0E-05	Alanine and Aspartate Metabolism
2,3-dihydroxy-2-methylbutyrate	0.65	6.4E-05	Leucine, Isoleucine and Valine Metabolism
3-indoxyl sulfate	1.61	7.3E-05	Tryptophan Metabolism
cysteine sulfinic acid	1.12	7.3E-05	Methionine, Cysteine, SAM and Taurine Metabolism
gentisate	0.53	7.8E-05	Tyrosine Metabolism
n-acetylmethionine sulfoxide	1.43	8.6E-05	Methionine, Cysteine, SAM and Taurine Metabolism
n2,n5-diacetylornithine	0.65	8.7E-05	Urea cycle; Arginine and Proline Metabolism
aspartate	1.29	8.7E-05	Alanine and Aspartate Metabolism
n-methylproline	0.44	9.0E-05	Urea cycle; Arginine and Proline Metabolism
indolepropionate	0.49	9.8E-05	Tryptophan Metabolism
c-glycosyltryptophan	1.16	1.0E-04	Tryptophan Metabolism
s-methylcysteine sulfoxide	0.52	1.1E-04	Methionine, Cysteine, SAM and Taurine Metabolism
pyroglutamine	0.66	1.1E-04	Glutamate Metabolism
s-methylcysteine	0.63	1.1E-04	Methionine, Cysteine, SAM and Taurine Metabolism
2-hydroxybutyrate/2-hydroxyisobutyrate	1.31	1.2E-04	Glutathione Metabolism
2-methylserine	0.51	1.3E-04	Glycine, Serine and Threonine Metabolism
beta-hydroxyisovalerate	1.46	1.3E-04	Leucine, Isoleucine and Valine Metabolism
n-acetylglutamate	1.23	1.3E-04	Glutamate Metabolism
n-delta-acetylornithine	0.56	1.6E-04	Urea cycle; Arginine and Proline Metabolism

kynurenine	1.19	1.6E-04	Tryptophan Metabolism
4-hydroxyglutamate	1.76	1.7E-04	Glutamate Metabolism
isoleucine	1.09	1.7E-04	Leucine, Isoleucine and Valine Metabolism
leucine	1.10	1.9E-04	Leucine, Isoleucine and Valine Metabolism
s-methylmethionine	0.22	2.0E-04	Methionine, Cysteine, SAM and Taurine Metabolism
isobutyrylcarnitine (c4)	1.48	2.0E-04	Leucine, Isoleucine and Valine Metabolism
indolelactate	1.23	2.0E-04	Tryptophan Metabolism
n-carbamoylalanine	1.90	2.1E-04	Alanine and Aspartate Metabolism
imidazole propionate	1.72	2.5E-04	Histidine Metabolism
n-acetylarginine	1.32	3.0E-04	Urea cycle; Arginine and Proline Metabolism
8-methoxykynurenate	1.51	3.4E-04	Tryptophan Metabolism
hypotaurine	0.72	3.5E-04	Methionine, Cysteine, SAM and Taurine Metabolism
n2-acetyllysine	1.46	3.7E-04	Lysine Metabolism
5-oxoproline	1.22	4.4E-04	Glutathione Metabolism
2-hydroxy-3-methylvalerate	1.29	4.6E-04	Leucine, Isoleucine and Valine Metabolism
n-acetyl-1-methylhistidine	1.69	4.8E-04	Histidine Metabolism
2-aminobutyrate	1.19	5.0E-04	Glutathione Metabolism
n-acetyltaurine	1.25	5.0E-04	Methionine, Cysteine, SAM and Taurine Metabolism
n-acetylglycine	0.74	5.8E-04	Glycine, Serine and Threonine Metabolism
5-methylthioadenosine (mta)	1.15	6.8E-04	Polyamine Metabolism
creatinine	1.08	6.9E-04	Creatine Metabolism
n-acetyltryptophan	1.27	7.0E-04	Tryptophan Metabolism
glutamine	0.84	7.8E-04	Glutamate Metabolism
beta-citrylglutamate	1.25	8.4E-04	Glutamate Metabolism
alpha-hydroxyisovalerate	1.30	9.5E-04	Leucine, Isoleucine and Valine Metabolism
glycine	0.85	1.0E-03	Glycine, Serine and Threonine Metabolism
glutarylcarnitine (c5-dc)	1.41	1.0E-03	Lysine Metabolism
n-acetylcitrulline	1.60	1.2E-03	Urea cycle; Arginine and Proline Metabolism
5-(galactosylhydroxy)-l-lysine	1.28	1.2E-03	Lysine Metabolism
3-methyl-2-oxobutyrate	1.15	1.3E-03	Leucine, Isoleucine and Valine Metabolism
4-methoxyphenol sulfate	0.59	1.4E-03	Tyrosine Metabolism
p-cresol glucuronide	2.39	1.5E-03	Tyrosine Metabolism
1-ribosyl-imidazoleacetate	0.82	1.5E-03	Histidine Metabolism
tryptophan betaine	0.58	1.8E-03	Tryptophan Metabolism
isobutyrylglycine (c4)	1.31	1.9E-03	Leucine, Isoleucine and Valine Metabolism
indoleacetate	1.41	2.0E-03	Tryptophan Metabolism
n-acetylalanine	1.09	2.0E-03	Alanine and Aspartate Metabolism
tyrosine	1.11	2.0E-03	Tyrosine Metabolism
carboxyethyl-gaba	0.83	2.0E-03	Glutamate Metabolism
prolylhydroxyproline	0.77	2.4E-03	Urea cycle; Arginine and Proline Metabolism
betaine	0.86	2.5E-03	Glycine, Serine and Threonine Metabolism
n-formylphenylalanine	1.32	2.5E-03	Tyrosine Metabolism
3-hydroxyisobutyrate	1.28	2.9E-03	Leucine, Isoleucine and Valine Metabolism
s-adenosylhomocysteine (sah)	1.14	3.3E-03	Methionine, Cysteine, SAM and Taurine Metabolism
3-(4-hydroxyphenyl)lactate (hpla)	1.18	3.5E-03	Tyrosine Metabolism
cysteinylglycine disulfide	0.87	4.2E-03	Glutathione Metabolism
hydroxyasparagine	1.10	4.2E-03	Alanine and Aspartate Metabolism
alpha-hydroxyisocaproate	1.17	5.4E-03	Leucine, Isoleucine and Valine Metabolism
n-acetylproline	1.40	5.4E-03	Urea cycle; Arginine and Proline Metabolism
dimethylglycine	1.31	5.5E-03	Glycine, Serine and Threonine Metabolism
cysteine s-sulfate	1.19	5.8E-03	Methionine, Cysteine, SAM and Taurine Metabolism
imidazole lactate	1.19	6.0E-03	Histidine Metabolism
alpha-ketoglutaramate	1.14	6.5E-03	Glutamate Metabolism
phenylacetate	1.54	6.7E-03	Phenylalanine Metabolism
hydroxy-n6,n6,n6-trimethyllysine	1.14	7.1E-03	Lysine Metabolism
vanillactate	1.17	7.6E-03	Tyrosine Metabolism
5-hydroxylysine	1.13	8.6E-03	Lysine Metabolism
3-methylglutarylcarnitine (2)	1.38	1.0E-02	Leucine, Isoleucine and Valine Metabolism
guanidinosuccinate	1.29	1.1E-02	Guanidino and Acetamido Metabolism
alanine	1.08	1.1E-02	Alanine and Aspartate Metabolism
4-hydroxyphenylacetatoylcarnitine	1.23	1.2E-02	Tyrosine Metabolism
threonine	1.10	1.3E-02	Glycine, Serine and Threonine Metabolism
hydantoin-5-propionate	1.25	1.3E-02	Histidine Metabolism
3-sulfo-l-alanine	1.33	1.3E-02	Methionine, Cysteine, SAM and Taurine Metabolism
cysteine-glutathione disulfide	0.68	1.5E-02	Glutathione Metabolism
proline	1.11	1.6E-02	Urea cycle; Arginine and Proline Metabolism

n-acetylcarnosine	1.21	1.6E-02	Histidine Metabolism
3-amino-2-piperidone	1.13	1.7E-02	Urea cycle; Arginine and Proline Metabolism
n-acetyl-isoputreanine	0.87	2.1E-02	Polyamine Metabolism
tyramine o-sulfate	1.54	2.3E-02	Tyrosine Metabolism
homoarginine	1.16	2.5E-02	Urea cycle; Arginine and Proline Metabolism
5-methylthioribose	0.93	2.5E-02	Methionine, Cysteine, SAM and Taurine Metabolism
6-bromotryptophan	0.89	3.0E-02	Tryptophan Metabolism
n-acetylputrescine	0.89	3.0E-02	Polyamine Metabolism
cysteine	1.07	3.3E-02	Methionine, Cysteine, SAM and Taurine Metabolism
4-guanidinobutanoate	1.22	3.5E-02	Guanidino and Acetamido Metabolism
fructosyllysine	1.16	3.5E-02	Lysine Metabolism
indolebutyrate	1.28	3.6E-02	Tryptophan Metabolism
guanidinoacetate	0.89	4.5E-02	Creatine Metabolism
n-formylmethionine	1.06	4.5E-02	Methionine, Cysteine, SAM and Taurine Metabolism
n-acetylisoleucine	1.17	4.6E-02	Leucine, Isoleucine and Valine Metabolism
n-acetylhistidine	1.11	4.8E-02	Histidine Metabolism

Table S2. Lipid metabolites associated with a vegan (relative to non-vegetarian) dietary pattern at FDR < 0.05 in linear regression models with SmartSVA approach.

Metabolite	Fold Change	FDR	Subclass
palmitoyl-linoleoyl-glycerol (16:0/18:2) [1]	1.88	<3.9E-05	Diacylglycerol
glycerophosphoethanolamine	1.16	<3.9E-05	Phospholipid Metabolism
1-stearoyl-2-docosahexaenoyl-gpe (18:0/22:6)	1.62	<3.9E-05	Phosphatidylethanolamine (PE)
glycerol	1.35	<3.9E-05	Glycerolipid Metabolism
choline	1.14	<3.9E-05	Phospholipid Metabolism
3,4-dihydroxybutyrate	1.20	<3.9E-05	Fatty Acid, Dihydroxy
docosahexaenoylcarnitine (c22:6)	1.80	<3.9E-05	Fatty Acid Metabolism (Acyl Carnitine, Polyunsaturated)
n-stearoyltaurine	1.37	<3.9E-05	Endocannabinoid
glutarate (c5-dc)	1.83	<3.9E-05	Fatty Acid, Dicarboxylate
myristoyl dihydrosphingomyelin (d18:0/14:0)	1.34	<3.9E-05	Dihydrosphingomyelins
docosahexaenoate (dha; 22:6n3)	1.47	<3.9E-05	Long Chain Polyunsaturated Fatty Acid (n3 and n6)
1-palmitoylglycerol (16:0)	1.46	<3.9E-05	Monoacylglycerol
trimethylamine n-oxide	1.53	<3.9E-05	Phospholipid Metabolism
myristoleate (14:1n5)	1.39	<3.9E-05	Long Chain Monounsaturated Fatty Acid
ceramide (d18:1/20:0, d16:1/22:0, d20:1/18:0)	1.42	<3.9E-05	Ceramides
stearoyl sphingomyelin (d18:1/18:0)	1.25	<3.9E-05	Sphingomyelins
n-stearoyl-sphingadienine (d18:2/18:0)	1.65	<3.9E-05	Ceramides
stearoylcarnitine (c18)	1.33	<3.9E-05	Fatty Acid Metabolism (Acyl Carnitine, Long Chain Saturated)
myristoylcarnitine (c14)	1.38	<3.9E-05	Fatty Acid Metabolism (Acyl Carnitine, Long Chain Saturated)
1-myristoyl-2-palmitoyl-gpc (14:0/16:0)	1.74	<3.9E-05	Phosphatidylcholine (PC)
palmitoylcarnitine (c16)	1.25	<3.9E-05	Fatty Acid Metabolism (Acyl Carnitine, Long Chain Saturated)
1-(1-enyl-palmitoyl)-gpe (p-16:0)	1.34	<3.9E-05	Lysoplasmalogen
1-(1-enyl-palmitoyl)-2-arachidonoyl-gpc (p-16:0/20:4)	1.32	<3.9E-05	Plasmalogen
sphingomyelin (d18:0/18:0, d19:0/17:0)	1.64	<3.9E-05	Dihydrosphingomyelins
palmitoyl ethanolamide	1.29	<3.9E-05	Endocannabinoid
sphingomyelin (d18:2/23:1)	1.35	<3.9E-05	Sphingomyelins
ceramide (d16:1/24:1, d18:1/22:1)	1.82	<3.9E-05	Ceramides
propionylcarnitine (c3)	1.65	<3.9E-05	Fatty Acid Metabolism (also BCAA Metabolism)
1-pentadecanoylglycerol (15:0)	1.51	<3.9E-05	Monoacylglycerol
1-palmitoleoylglycerol (16:1)	1.65	<3.9E-05	Monoacylglycerol
docosapentaenoate (n6 dpa; 22:5n6)	1.59	<3.9E-05	Long Chain Polyunsaturated Fatty Acid (n3 and n6)
palmitoleate (16:1n7)	1.48	<3.9E-05	Long Chain Monounsaturated Fatty Acid
stearate (18:0)	1.22	<3.9E-05	Long Chain Saturated Fatty Acid
n-stearoyl-sphinganine (d18:0/18:0)	2.17	<3.9E-05	Dihydroceramides
10-undecenoate (11:1n1)	1.77	<3.9E-05	Medium Chain Fatty Acid
palmitate (16:0)	1.25	<3.9E-05	Long Chain Saturated Fatty Acid
1-(1-enyl-palmitoyl)-2-linoleoyl-gpe (p-16:0/18:2)	1.46	<3.9E-05	Plasmalogen
n-stearoyl-sphingosine (d18:1/18:0)	1.79	<3.9E-05	Ceramides
1-(1-enyl-stearoyl)-2-linoleoyl-gpe (p-18:0/18:2)	1.46	<3.9E-05	Plasmalogen
ceramide (d18:1/14:0, d16:1/16:0)	1.91	<3.9E-05	Ceramides
1-stearoyl-gpg (18:0)	1.89	<3.9E-05	Lysophospholipid
sphingomyelin (d18:1/21:0, d17:1/22:0, d16:1/23:0)	1.49	<3.9E-05	Sphingomyelins
3-carboxy-4-methyl-5-pentyl-2-furanpropionate (3-cmpfp)	1.89	<3.9E-05	Fatty Acid, Dicarboxylate
1-(1-enyl-stearoyl)-2-oleoyl-gpe (p-18:0/18:1)	1.57	<3.9E-05	Plasmalogen
2r,3r-dihydroxybutyrate	1.68	<3.9E-05	Fatty Acid, Dihydroxy
1-myristoylglycerol (14:0)	1.99	<3.9E-05	Monoacylglycerol
1-(1-enyl-palmitoyl)-2-arachidonoyl-gpe (p-16:0/20:4)	1.62	<3.9E-05	Plasmalogen
sphingomyelin (d18:1/19:0, d19:1/18:0)	1.56	<3.9E-05	Sphingomyelins
picolinoylglycine	1.84	<3.9E-05	Fatty Acid Metabolism (Acyl Glycine)
hydroxy-cmpf	11.34	<3.9E-05	Fatty Acid, Dicarboxylate
ceramide (d18:1/17:0, d17:1/18:0)	2.36	<3.9E-05	Ceramides
sphingomyelin (d18:1/14:0, d16:1/16:0)	1.51	<3.9E-05	Sphingomyelins
1-(1-enyl-stearoyl)-gpe (p-18:0)	1.75	<3.9E-05	Lysoplasmalogen
myristate (14:0)	1.67	<3.9E-05	Long Chain Saturated Fatty Acid
1-(1-enyl-stearoyl)-2-arachidonoyl-gpe (p-18:0/20:4)	1.81	<3.9E-05	Plasmalogen
undecenoylcarnitine (c11:1)	2.18	<3.9E-05	Fatty Acid Metabolism (Acyl Carnitine, Monounsaturated)
tridecenedioate (c13:1-dc)	2.79	<3.9E-05	Fatty Acid, Dicarboxylate
1-margaroylglycerol (17:0)	3.09	<3.9E-05	Monoacylglycerol
sphingomyelin (d18:1/17:0, d17:1/18:0, d19:1/16:0)	1.49	<3.9E-05	Sphingomyelins
10-nonadecenoate (19:1n9)	1.76	<3.9E-05	Long Chain Monounsaturated Fatty Acid

sphingomyelin (d18:1/25:0, d19:0/24:1, d20:1/23:0, d19:1/24:0)	2.59	<3.9E-05	Sphingomyelins
sphingomyelin (d17:1/14:0, d16:1/15:0)	1.94	<3.9E-05	Sphingomyelins
n-palmitoyl-heptadecasphingosine (d17:1/16:0)	2.10	<3.9E-05	Ceramides
sphingomyelin (d17:2/16:0, d18:2/15:0)	1.91	<3.9E-05	Sphingomyelins
3-carboxy-4-methyl-5-propyl-2-furanpropanoate (cmpf)	19.15	<3.9E-05	Fatty Acid, Dicarboxylate
sphingomyelin (d17:1/16:0, d18:1/15:0, d16:1/17:0)	1.58	<3.9E-05	Sphingomyelins
(16 or 17)-methylstearate (a19:0 or i19:0)	2.43	<3.9E-05	Fatty Acid, Branched
margarate (17:0)	1.64	<3.9E-05	Long Chain Saturated Fatty Acid
heptenedioate (c7:1-dc)	3.68	<3.9E-05	Fatty Acid, Dicarboxylate
pentadecanoate (15:0)	1.52	<3.9E-05	Long Chain Saturated Fatty Acid
10-heptadecenoate (17:1n7)	2.07	<3.9E-05	Long Chain Monounsaturated Fatty Acid
(12 or 13)-methylmyristate (a15:0 or i15:0)	3.30	<3.9E-05	Fatty Acid, Branched
margaroylcarnitine (c17)	2.41	<3.9E-05	Fatty Acid Metabolism (Acyl Carnitine, Long Chain Saturated)
(14 or 15)-methylpalmitate (a17:0 or i17:0)	3.44	<3.9E-05	Fatty Acid, Branched
stearoyl ethanolamide	1.22	3.9E-05	Endocannabinoid
cortolone glucuronide (1)	1.40	5.8E-05	Corticosteroids
1-palmitoyl-2-palmitoleoyl-gpc (16:0/16:1)	1.35	5.8E-05	Phosphatidylcholine (PC)
n-linoleoylglycine	0.59	6.2E-05	Fatty Acid Metabolism (Acyl Glycine)
(2 or 3)-decenoate (10:1n7 or n8)	0.66	6.3E-05	Medium Chain Fatty Acid
linolenoylcarnitine (c18:3)	0.77	6.8E-05	Fatty Acid Metabolism (Acyl Carnitine, Polyunsaturated)
glycosyl ceramide (d18:2/24:1, d18:1/24:2)	0.72	7.1E-05	Hexosylceramides (HCER)
behenate (22:0)	1.68	7.1E-05	Long Chain Saturated Fatty Acid
n-behenoyl-sphingadienine (d18:2/22:0)	1.36	7.2E-05	Ceramides
palmitoyl-linoleoyl-glycerol (16:0/18:2) [2]	1.55	7.2E-05	Diacylglycerol
3-hydroxydecanoate	0.74	7.3E-05	Fatty Acid, Monohydroxy
1-oleoyl-gpc (18:1)	0.83	8.3E-05	Lysophospholipid
sphingomyelin (d18:2/24:2)	0.78	8.5E-05	Sphingomyelins
nonanoylcarnitine (c9)	1.42	8.8E-05	Fatty Acid Metabolism (Acyl Carnitine, Medium Chain)
adrenate (22:4n6)	1.29	8.8E-05	Long Chain Polyunsaturated Fatty Acid (n3 and n6)
12,13-dihome	0.60	9.5E-05	Fatty Acid, Dihydroxy
tetradecadienoate (14:2)	0.70	1.0E-04	Long Chain Polyunsaturated Fatty Acid (n3 and n6)
1-palmitoyl-2-docosahexaenoyl-gpe (16:0/22:6)	1.45	1.0E-04	Phosphatidylethanolamine (PE)
1-cerotoyl-gpc (26:0)	0.64	1.0E-04	Lysophospholipid
1-linoleoyl-gpe (18:2)	0.78	1.1E-04	Lysophospholipid
dodecadienoate (12:2)	0.66	1.1E-04	Fatty Acid, Dicarboxylate
octadecadienedioate (c18:2-dc)	0.57	1.2E-04	Fatty Acid, Dicarboxylate
deoxycholic acid 12-sulfate	2.01	1.2E-04	Secondary Bile Acid Metabolism
sphingomyelin (d18:1/24:1, d18:2/24:0)	0.87	1.2E-04	Sphingomyelins
cis-4-decenoate (10:1n6)	0.61	1.2E-04	Medium Chain Fatty Acid
lactosyl-n-nervonoyl-sphingosine (d18:1/24:1)	0.72	1.3E-04	Lactosylceramides (LCER)
sphingomyelin (d18:2/18:1)	1.27	1.3E-04	Sphingomyelins
1,2-dilinoleoyl-gpc (18:2/18:2)	0.76	1.4E-04	Phosphatidylcholine (PC)
octadecenedioylcarnitine (c18:1-dc)	0.59	1.4E-04	Fatty Acid Metabolism (Acyl Carnitine, Dicarboxylate)
1-docosahexaenoylglycerol (22:6)	1.57	1.5E-04	Monoacylglycerol
acetoacetate	0.61	1.6E-04	Ketone Bodies
trans-2-hexenoylglycine	0.63	1.7E-04	Fatty Acid Metabolism (Acyl Glycine)
3-hydroxybutyrate (bhba)	0.54	1.7E-04	Ketone Bodies
13-hode + 9-hode	1.27	1.8E-04	Fatty Acid, Monohydroxy
dodecenedioate (c12:1-dc)	0.53	1.8E-04	Fatty Acid, Dicarboxylate
sphingomyelin (d18:0/20:0, d16:0/22:0)	1.38	1.8E-04	Dihydrosphingomyelins
sphingomyelin (d18:1/18:1, d18:2/18:0)	1.18	1.9E-04	Sphingomyelins
1-linoleoyl-2-arachidonoyl-gpc (18:2/20:4n6)	0.84	2.1E-04	Phosphatidylcholine (PC)
3-hydroxysebacate	0.61	2.1E-04	Fatty Acid, Monohydroxy
lactosyl-n-palmitoyl-sphingosine (d18:1/16:0)	0.83	2.1E-04	Lactosylceramides (LCER)
1-(1-enyl-palmitoyl)-gpc (p-16:0)	1.19	2.1E-04	Lysoplasmalogen
palmitoleoyl-linoleoyl-glycerol (16:1/18:2) [1]	1.60	2.1E-04	Diacylglycerol
isoursodeoxycholate	2.00	2.1E-04	Secondary Bile Acid Metabolism
branched chain 14:0 dicarboxylic acid	0.28	2.2E-04	Fatty Acid, Dicarboxylate
linoleoyl-docosahexaenoyl-glycerol (18:2/22:6) [2]	1.69	2.4E-04	Diacylglycerol
1-linoleoyl-gpc (18:2)	0.81	2.4E-04	Lysophospholipid
dodecanedioate (c12)	0.67	2.5E-04	Fatty Acid, Dicarboxylate
sphinganine	1.26	2.7E-04	Sphingolipid Synthesis
1-palmitoyl-gpg (16:0)	1.47	3.1E-04	Lysophospholipid
sphingomyelin (d18:2/24:1, d18:1/24:2)	0.81	3.1E-04	Sphingomyelins

2-hydroxyadipate	1.63	3.3E-04	Fatty Acid, Dicarboxylate
oleoyl-arachidonoyl-glycerol (18:1/20:4) [2]	1.40	3.3E-04	Diacylglycerol
octadecenedioate (c18:1-dc)	0.55	3.5E-04	Fatty Acid, Dicarboxylate
2-hydroxyglutarate	1.20	3.7E-04	Fatty Acid, Dicarboxylate
3-hydroxydodecanedioate	0.57	3.9E-04	Fatty Acid, Dicarboxylate
tetradecadienedioate (c14:2-dc)	0.69	3.9E-04	Fatty Acid, Dicarboxylate
1-linoleoyl-2-linolenoyl-gpc (18:2/18:3)	0.52	4.5E-04	Phosphatidylcholine (PC)
sphingomyelin (d18:2/14:0, d18:1/14:1)	1.27	4.9E-04	Sphingomyelins
3-hydroxyoctanoate	0.78	5.5E-04	Fatty Acid, Monohydroxy
n-linoleoyltaurine	0.68	5.8E-04	Endocannabinoid
lignoceroylcarnitine (c24)	1.35	5.8E-04	Fatty Acid Metabolism (Acyl Carnitine, Long Chain Saturated)
malonate	0.79	5.9E-04	Fatty Acid Synthesis
palmitoyl-sphingosine-phosphoethanolamine (d18:1/16:0)	0.86	6.2E-04	Ceramide PEs
glycohyocholate	0.35	6.3E-04	Secondary Bile Acid Metabolism
2-aminoheptanoate	0.79	7.7E-04	Fatty Acid, Amino
hexanoylglutamine	0.70	8.1E-04	Fatty Acid Metabolism (Acyl Glutamine)
2-palmitoleoylglycerol (16:1)	1.69	8.3E-04	Monoacylglycerol
sebacate (c10-dc)	0.66	8.7E-04	Fatty Acid, Dicarboxylate
sphingosine	1.25	1.0E-03	Sphingosines
1-palmitoyl-2-docosahexaenoyl-gpc (16:0/22:6)	1.16	1.1E-03	Phosphatidylcholine (PC)
3-hydroxylaurate	0.77	1.2E-03	Fatty Acid, Monohydroxy
3-hydroxybutyroylglycine	0.70	1.2E-03	Fatty Acid Metabolism (Acyl Glycine)
2-hydroxysebacate	0.72	1.2E-03	Fatty Acid, Dicarboxylate
3-hydroxy-3-methylglutarate	1.16	1.2E-03	Mevalonate Metabolism
14-hdohe/17-hdohe	1.45	1.2E-03	Docosanoid
behenoyl sphingomyelin (d18:1/22:0)	1.16	1.2E-03	Sphingomyelins
chiro-inositol	0.61	1.2E-03	Inositol Metabolism
ursodeoxycholate	2.27	1.3E-03	Secondary Bile Acid Metabolism
1-dihomo-linolenylglycerol (20:3)	1.26	1.7E-03	Monoacylglycerol
1-stearoyl-2-oleoyl-gpc (18:0/18:1)	1.13	1.8E-03	Phosphatidylcholine (PC)
nonadecanoate (19:0)	1.14	1.8E-03	Long Chain Saturated Fatty Acid
arachidoylcarnitine (c20)	1.24	1.8E-03	Fatty Acid Metabolism (Acyl Carnitine, Long Chain Saturated)
linolenate (18:3n3 or 3n6)	0.79	1.9E-03	Long Chain Polyunsaturated Fatty Acid (n3 and n6)
1-stearoyl-2-docosahexaenoyl-gpc (18:0/22:6)	1.23	2.2E-03	Phosphatidylcholine (PC)
glycochenodeoxycholate 3-sulfate	0.53	2.3E-03	Primary Bile Acid Metabolism
1-linoleoyl-gpa (18:2)	1.23	2.5E-03	Lysophospholipid
1-linolenoyl-gpc (18:3)	0.83	2.7E-03	Lysophospholipid
2-hydroxynervonate	0.82	2.7E-03	Fatty Acid, Monohydroxy
1-palmitoyl-gpe (16:0)	1.13	2.8E-03	Lysophospholipid
sphingomyelin (d18:2/21:0, d16:2/23:0)	1.19	2.8E-03	Sphingomyelins
n-palmitoyl-sphingosine (d18:1/16:0)	1.15	2.8E-03	Ceramides
2-butenoylglycine	0.68	2.9E-03	Fatty Acid Metabolism (Acyl Glycine)
cortisone	0.79	2.9E-03	Corticosteroids
sphingomyelin (d18:1/20:0, d16:1/22:0)	1.11	3.1E-03	Sphingomyelins
eicosapentaenoate (epa; 20:5n3)	1.27	3.1E-03	Long Chain Polyunsaturated Fatty Acid (n3 and n6)
1-lignoceroyl-gpc (24:0)	0.62	3.1E-03	Lysophospholipid
butyrate/isobutyrate (4:0)	1.30	3.2E-03	Short Chain Fatty Acid
behenoylcarnitine (c22)	1.30	3.2E-03	Fatty Acid Metabolism (Acyl Carnitine, Long Chain Saturated)
taurodeoxycholate	2.07	3.2E-03	Secondary Bile Acid Metabolism
docosatrienoate (22:3n6)	1.56	3.3E-03	Long Chain Polyunsaturated Fatty Acid (n3 and n6)
nisinate (24:6n3)	1.79	3.4E-03	Long Chain Polyunsaturated Fatty Acid (n3 and n6)
1-(1-enyl-oleoyl)-gpe (p-18:1)	1.16	3.5E-03	Lysoplasmalogen
butyrylcarnitine (c4)	1.32	3.8E-03	Fatty Acid Metabolism (also BCAA Metabolism)
hexadecanedioate (c16)	0.80	3.8E-03	Fatty Acid, Dicarboxylate
9,10-dihome	0.73	3.8E-03	Fatty Acid, Dihydroxy
3-hydroxyadipate	0.78	4.1E-03	Fatty Acid, Dicarboxylate
linoleoylcarnitine (c18:2)	0.82	4.1E-03	Fatty Acid Metabolism (Acyl Carnitine, Polyunsaturated)
3beta-hydroxy-5-cholestenoate	0.84	4.2E-03	Sterol
carnitine	1.24	4.3E-03	Carnitine Metabolism
oleoyl-arachidonoyl-glycerol (18:1/20:4) [1]	1.29	4.3E-03	Diacylglycerol
n-palmitoyl-sphinganine (d18:0/16:0)	1.21	4.4E-03	Dihydroceramides
9-hydroxystearate	1.34	4.6E-03	Fatty Acid, Monohydroxy
1-palmitoleoyl-2-linolenoyl-gpc (16:1/18:3)	0.71	4.9E-03	Phosphatidylcholine (PC)
deoxycholic acid glucuronide	1.73	5.0E-03	Secondary Bile Acid Metabolism
docosapentaenoate (dpa; 22:5n3)	1.17	5.5E-03	Long Chain Polyunsaturated Fatty Acid (n3 and n6)

caproate (6:0)	1.15	5.5E-03	Medium Chain Fatty Acid
2-hydroxystearate	1.10	5.5E-03	Fatty Acid, Monohydroxy
androstenediol (3alpha, 17alpha) monosulfate (3)	0.72	5.5E-03	Androgenic Steroids
1-stearoyl-2-oleoyl-gpe (18:0/18:1)	1.15	5.7E-03	Phosphatidylethanolamine (PE)
pregnen-diol disulfate	0.75	6.3E-03	Pregnenolone Steroids
lithocholate sulfate (1)	1.58	6.5E-03	Secondary Bile Acid Metabolism
adipoylcarnitine (c6-dc)	1.31	6.5E-03	Fatty Acid Metabolism (Acyl Carnitine, Dicarboxylate)
dihomolinolenate (20:3n3 or 3n6)	1.14	6.9E-03	Long Chain Polyunsaturated Fatty Acid (n3 and n6)
behenoyl dihydrosphingomyelin (d18:0/22:0)	1.28	7.0E-03	Dihydrosphingomyelins
docosapentaenoylcarnitine (c22:5n3)	1.26	7.0E-03	Fatty Acid Metabolism (Acyl Carnitine, Polyunsaturated)
taurodeoxycholic acid 3-sulfate	1.70	7.1E-03	Secondary Bile Acid Metabolism
5alpha-androstan-3alpha,17beta-diol monosulfate (1)	1.37	7.5E-03	Androgenic Steroids
11beta-hydroxyandrosterone glucuronide	1.30	7.6E-03	Androgenic Steroids
hexadecadienoate (16:2n6)	1.17	7.6E-03	Long Chain Polyunsaturated Fatty Acid (n3 and n6)
sphingomyelin (d18:1/22:2, d18:2/22:1, d16:1/24:2)	1.12	8.1E-03	Sphingomyelins
erucoylcarnitine (c22:1)	1.35	8.7E-03	Fatty Acid Metabolism (Acyl Carnitine, Monounsaturated)
sphingadienine	1.23	8.7E-03	Sphingolipid Synthesis
n-palmitoyl-sphingadienine (d18:2/16:0)	1.13	8.7E-03	Ceramides
5alpha-androstan-3beta,17beta-diol monosulfate (2)	1.31	9.6E-03	Androgenic Steroids
2s,3r-dihydroxybutyrate	1.19	9.6E-03	Fatty Acid, Dihydroxy
n-oleoyltaurine	0.84	9.9E-03	Endocannabinoid
undecanoate (11:0)	1.07	1.0E-02	Medium Chain Fatty Acid
1-linoleoyl-gpi (18:2)	0.86	1.0E-02	Lysophospholipid
glycerophosphoglycerol	1.37	1.0E-02	Glycerolipid Metabolism
glycoursodeoxycholic acid sulfate (1)	1.69	1.1E-02	Secondary Bile Acid Metabolism
17alpha-hydroxypregnenolone 3-sulfate	0.74	1.1E-02	Pregnenolone Steroids
maleate	1.35	1.1E-02	Fatty Acid, Dicarboxylate
docosahexaenoylcholine	1.26	1.1E-02	Fatty Acid Metabolism (Acyl Choline)
lignoceroyl sphingomyelin (d18:1/24:0)	0.88	1.1E-02	Sphingomyelins
glycocholate	0.66	1.1E-02	Primary Bile Acid Metabolism
pregnenediol sulfate (c21h34o5s)	0.80	1.1E-02	Pregnenolone Steroids
1-stearoyl-2-linoleoyl-gpi (18:0/18:2)	0.86	1.2E-02	Phosphatidylinositol (PI)
palmitoyl dihydrosphingomyelin (d18:0/16:0)	0.89	1.3E-02	Dihydrosphingomyelins
phosphocholine	1.12	1.3E-02	Phospholipid Metabolism
2-hydroxydecanoate	0.76	1.4E-02	Fatty Acid, Monohydroxy
3b-hydroxy-5-cholenoic acid	0.74	1.4E-02	Secondary Bile Acid Metabolism
1,2-dilinoleoyl-gpe (18:2/18:2)	0.75	1.4E-02	Phosphatidylethanolamine (PE)
2-stearoyl-gpe (18:0)	1.12	1.4E-02	Lysophospholipid
3-hydroxyhexanoate	0.87	1.4E-02	Fatty Acid, Monohydroxy
hexanoylcarnitine (c6)	1.17	1.5E-02	Fatty Acid Metabolism (Acyl Carnitine, Medium Chain)
1-palmitoyl-gpc (16:0)	0.94	1.5E-02	Lysophospholipid
3-hydroxydecanoylcarnitine	0.79	1.5E-02	Fatty Acid Metabolism (Acyl Carnitine, Hydroxy)
propionylglycine (c3)	1.27	1.5E-02	Fatty Acid Metabolism (also BCAA Metabolism)
heptanoate (7:0)	1.16	1.7E-02	Medium Chain Fatty Acid
pregnanediol-3-glucuronide	1.31	1.7E-02	Progestin Steroids
glycochenodeoxycholate	0.70	1.8E-02	Primary Bile Acid Metabolism
1-oleoyl-2-linoleoyl-gpe (18:1/18:2)	0.84	1.8E-02	Phosphatidylethanolamine (PE)
1-(1-enyl-palmitoyl)-2-oleoyl-gpc (p-16:0/18:1)	0.90	1.8E-02	Plasmalogen
sphingosine 1-phosphate	0.90	1.8E-02	Sphingosines
caprate (10:0)	1.17	1.9E-02	Medium Chain Fatty Acid
1-oleoylglycerol (18:1)	1.19	1.9E-02	Monoacylglycerol
1-palmitoyl-2-oleoyl-gpe (16:0/18:1)	1.14	1.9E-02	Phosphatidylethanolamine (PE)
3-methyladipate	1.33	2.0E-02	Fatty Acid, Dicarboxylate
nervonoylcarnitine (c24:1)	1.17	2.2E-02	Fatty Acid Metabolism (Acyl Carnitine, Monounsaturated)
1-oleoyl-gpe (18:1)	0.86	2.4E-02	Lysophospholipid
1-arachidonoyl-gpc (20:4)	0.89	2.4E-02	Lysophospholipid
2-methylmalonylcarnitine (c4-dc)	1.12	2.4E-02	Fatty Acid Metabolism (also BCAA Metabolism)
palmitoloelycholine	1.20	2.6E-02	Fatty Acid Metabolism (Acyl Choline)
sphingomyelin (d18:1/20:2, d18:2/20:1, d16:1/22:2)	1.12	2.7E-02	Sphingomyelins
3beta,7alpha-dihydroxy-5-cholestenoate	0.81	2.8E-02	Sterol
beta-sitosterol	0.77	2.8E-02	Sterol
adrenoylcarnitine (c22:4)	1.19	2.8E-02	Fatty Acid Metabolism (Acyl Carnitine, Polyunsaturated)
2-oleoylglycerol (18:1)	1.23	2.9E-02	Monoacylglycerol
2-hydroxyoctanoate	1.22	2.9E-02	Fatty Acid, Monohydroxy
androsterone glucuronide	1.30	2.9E-02	Androgenic Steroids
pregnenolone sulfate	0.75	2.9E-02	Pregnenolone Steroids

2-hydroxypalmitate	1.07	3.0E-02	Fatty Acid, Monohydroxy
docosadienoate (22:2n6)	0.89	3.0E-02	Long Chain Polyunsaturated Fatty Acid (n3 and n6)
myo-inositol	0.91	3.2E-02	Inositol Metabolism
5alpha-androstan-3alpha,17alpha-diol monosulfate	1.22	3.2E-02	Androgenic Steroids
n-acetyl-2-aminooctanoate	1.22	3.4E-02	Fatty Acid, Amino
1-arachidonoyl-gpe (20:4n6)	0.91	3.4E-02	Lysophospholipid
pimeloylcarnitine/3-methyladipoylcarnitine (c7-dc)	1.22	3.5E-02	Fatty Acid Metabolism (Acyl Carnitine, Dicarboxylate)
1-palmitoyl-2-linoleoyl-gpe (16:0/18:2)	1.12	3.6E-02	Phosphatidylethanolamine (PE)
1-oleoyl-2-arachidonoyl-gpe (18:1/20:4)	0.87	3.6E-02	Phosphatidylethanolamine (PE)
4-cholesten-3-one	1.09	3.9E-02	Sterol
1-myristoyl-2-arachidonoyl-gpc (14:0/20:4)	1.15	4.0E-02	Phosphatidylcholine (PC)
1-palmitoyl-gpa (16:0)	1.13	4.3E-02	Lysophospholipid
cerotoylcarnitine (c26)	1.16	4.3E-02	Fatty Acid Metabolism (Acyl Carnitine, Long Chain Saturated)
12-hete	1.22	4.4E-02	Eicosanoid
deoxycholate	1.20	4.5E-02	Secondary Bile Acid Metabolism
1-palmitoyl-2-dihomo-linolenoyl-gpc (16:0/20:3n3 or 6)	0.95	4.6E-02	Phosphatidylcholine (PC)
1-stearoyl-2-linoleoyl-gpc (18:0/18:2)	0.96	4.7E-02	Phosphatidylcholine (PC)
1-palmitoyl-2-oleoyl-gpi (16:0/18:1)	1.12	4.7E-02	Phosphatidylinositol (PI)
glycochenodeoxycholate glucuronide (1)	1.32	4.7E-02	Primary Bile Acid Metabolism
glycerol 3-phosphate	1.10	4.8E-02	Glycerolipid Metabolism
dehydroepiandrosterone sulfate (dhea-s)	0.85	4.8E-02	Androgenic Steroids
glyco-beta-muricholate	0.73	4.9E-02	Primary Bile Acid Metabolism
2-palmitoylglycerol (16:0)	1.38	5.0E-02	Monoacylglycerol

Table S3. Carbohydrate, cofactor/vitamin, and energy metabolites associated with a vegan (relative to non-vegetarian) dietary pattern at FDR < 0.05 in linear regression models with SmartSVA approach.

Metabolite	Fold Change	FDR	Subclass	Major Class
glucuronate	1.33	<3.9E-05	Aminosugar Metabolism	Carbohydrate
ribitol	1.16	7.3E-05	Pentose Metabolism	Carbohydrate
sucrose	1.62	3.3E-04	Disaccharides and Oligosaccharides	Carbohydrate
ribonate	1.14	3.6E-04	Pentose Metabolism	Carbohydrate
erythronate	1.10	3.8E-04	Aminosugar Metabolism	Carbohydrate
lactate	1.13	2.3E-03	Glycolysis, Gluconeogenesis, and Pyruvate Metabolism	Carbohydrate
pyruvate	1.25	8.1E-03	Glycolysis, Gluconeogenesis, and Pyruvate Metabolism	Carbohydrate
fructose	1.12	2.1E-02	Fructose, Mannose and Galactose Metabolism	Carbohydrate
maltose	1.16	2.2E-02	Glycogen Metabolism	Carbohydrate
galactonate	1.63	4.6E-02	Fructose, Mannose and Galactose Metabolism	Carbohydrate
quinolinate	1.56	<3.9E-05	Nicotinate and Nicotinamide Metabolism	Cofactors and Vitamins
carotene diol (1)	0.65	6.5E-05	Vitamin A Metabolism	Cofactors and Vitamins
carotene diol (2)	0.60	8.0E-05	Vitamin A Metabolism	Cofactors and Vitamins
carotene diol (3)	0.65	5.6E-04	Vitamin A Metabolism	Cofactors and Vitamins
threonate	0.79	8.4E-04	Ascorbate and Aldarate Metabolism	Cofactors and Vitamins
beta-cryptoxanthin	0.40	1.0E-03	Vitamin A Metabolism	Cofactors and Vitamins
oxalate (ethanedioate)	0.79	2.1E-03	Ascorbate and Aldarate Metabolism	Cofactors and Vitamins
n1-methyl-2-pyridone-5-carboxamide	1.42	2.5E-03	Nicotinate and Nicotinamide Metabolism	Cofactors and Vitamins
pantoate	1.38	2.8E-03	Pantothenate and CoA Metabolism	Cofactors and Vitamins
gulonate	1.16	3.8E-03	Ascorbate and Aldarate Metabolism	Cofactors and Vitamins
retinol (vitamin a)	1.10	1.7E-02	Vitamin A Metabolism	Cofactors and Vitamins
retinal	1.22	1.7E-02	Vitamin A Metabolism	Cofactors and Vitamins
pyridoxate	0.75	2.8E-02	Vitamin B6 Metabolism	Cofactors and Vitamins
pyridoxal	0.84	3.5E-02	Vitamin B6 Metabolism	Cofactors and Vitamins
gamma-tocopherol/beta-tocopherol	1.24	4.2E-02	Tocopherol Metabolism	Cofactors and Vitamins
delta-cehc glucuronide	1.26	4.7E-02	Tocopherol Metabolism	Cofactors and Vitamins
2-methylcitrate/homocitrate	1.31	<3.9E-05	TCA Cycle	Energy
isocitrate	0.73	7.5E-05	TCA Cycle	Energy
citraconate/glutaconate	1.41	8.7E-05	TCA Cycle	Energy
succinate	1.15	5.6E-04	TCA Cycle	Energy
citrate	0.83	7.8E-04	TCA Cycle	Energy
alpha-ketoglutarate	1.20	3.9E-03	TCA Cycle	Energy
aconitate [cis or trans]	0.90	2.6E-02	TCA Cycle	Energy

Table S4. Nucleotides, partially characterized molecules, and peptides associated with a vegan (relative to non-vegetarian) dietary pattern at FDR < 0.05 in linear regression models with SmartSVA approach.

Metabolite	Fold Change	FDR	Subclass	Major Class
n-acetyl-beta-alanine	1.20	<3.9E-05	Pyrimidine Metabolism, Uracil containing	Nucleotide
beta-alanine	1.25	<3.9E-05	Pyrimidine Metabolism, Uracil containing	Nucleotide
adenine	1.25	<3.9E-05	Purine Metabolism, Adenine containing	Nucleotide
orotidine	1.31	<3.9E-05	Pyrimidine Metabolism, Orotate containing	Nucleotide
xanthosine	1.48	5.7E-05	Purine Metabolism, (Hypo)Xanthine/Inosine containing	Nucleotide
n6-carbamoylthreonyladenosine	1.17	5.7E-05	Purine Metabolism, Adenine containing	Nucleotide
n2,n2-dimethylguanosine	1.15	7.4E-05	Purine Metabolism, Guanine containing	Nucleotide
3-(3-amino-3-carboxypropyl)uridine	1.18	8.9E-05	Pyrimidine Metabolism, Uracil containing	Nucleotide
n1-methylinosine	1.20	1.0E-04	Purine Metabolism, (Hypo)Xanthine/Inosine containing	Nucleotide
7-methylguanine	1.12	1.7E-04	Purine Metabolism, Guanine containing	Nucleotide
5,6-dihydrothymine	1.22	3.7E-04	Pyrimidine Metabolism, Thymine containing	Nucleotide
pseudouridine	1.11	5.2E-04	Pyrimidine Metabolism, Uracil containing	Nucleotide
urate	1.16	1.0E-03	Purine Metabolism, (Hypo)Xanthine/Inosine containing	Nucleotide
uracil	1.22	1.8E-03	Pyrimidine Metabolism, Uracil containing	Nucleotide
hypoxanthine	1.27	2.8E-03	Purine Metabolism, (Hypo)Xanthine/Inosine containing	Nucleotide
allantoin	1.19	3.5E-03	Purine Metabolism, (Hypo)Xanthine/Inosine containing	Nucleotide
n-carbamoylaspartate	1.24	6.5E-03	Pyrimidine Metabolism, Orotate containing	Nucleotide
guanosine	1.37	6.6E-03	Purine Metabolism, Guanine containing	Nucleotide
1-methyladenosine	1.06	7.0E-03	Purine Metabolism, Adenine containing	Nucleotide
orotate	1.12	2.0E-02	Pyrimidine Metabolism, Orotate containing	Nucleotide
glutamine conjugate of c7h12o2	1.97	<3.9E-05	Partially Characterized Molecules	Partially Characterized
pentose acid	0.62	7.7E-05	Partially Characterized Molecules	Partially Characterized
carnitine of c10h14o2 (5)	1.50	3.9E-04	Partially Characterized Molecules	Partially Characterized
glycine conjugate of c10h12o2	1.49	5.8E-04	Partially Characterized Molecules	Partially Characterized
glutamine conjugate of c6h10o2 (2)	0.62	1.2E-03	Partially Characterized Molecules	Partially Characterized
branched-chain, straight-chain, or cyclopropyl 10:1 fatty acid (1)	1.34	2.4E-03	Partially Characterized Molecules	Partially Characterized
glycine conjugate of c10h14o2 (1)	1.43	4.9E-03	Partially Characterized Molecules	Partially Characterized
branched-chain, straight-chain, or cyclopropyl 10:1 fatty acid (3)	1.37	2.1E-02	Partially Characterized Molecules	Partially Characterized
gamma-glutamylisoleucine	1.24	<3.9E-05	Gamma-glutamyl Amino Acid	Peptide
phenylacetylglutamate	1.70	<3.9E-05	Acetylated Peptides	Peptide
gamma-glutamylphenylalanine	1.24	<3.9E-05	Gamma-glutamyl Amino Acid	Peptide
gamma-glutamylleucine	1.26	<3.9E-05	Gamma-glutamyl Amino Acid	Peptide
gamma-glutamylvaline	1.33	<3.9E-05	Gamma-glutamyl Amino Acid	Peptide
gamma-glutamyltyrosine	1.27	5.7E-05	Gamma-glutamyl Amino Acid	Peptide
4-hydroxyphenylacetylglutamine	1.57	7.4E-05	Acetylated Peptides	Peptide
gamma-glutamylglycine	0.76	9.2E-05	Gamma-glutamyl Amino Acid	Peptide
phenylacetylcarnitine	2.38	1.0E-04	Acetylated Peptides	Peptide
gamma-glutamylglutamine	0.67	1.6E-03	Gamma-glutamyl Amino Acid	Peptide
gamma-glutamylcitrulline	0.83	2.8E-03	Gamma-glutamyl Amino Acid	Peptide
hwesasxx	1.32	5.4E-03	Polypeptide	Peptide
gamma-glutamyl-alpha-lysine	1.12	6.6E-03	Gamma-glutamyl Amino Acid	Peptide
phenylacetylglutamine	1.33	1.3E-02	Acetylated Peptides	Peptide
gamma-glutamylthreonine	1.11	1.3E-02	Gamma-glutamyl Amino Acid	Peptide
gamma-glutamylglutamate	1.34	1.5E-02	Gamma-glutamyl Amino Acid	Peptide
prolylglycine	1.16	1.9E-02	Dipeptide	Peptide
gamma-glutamylhistidine	0.91	3.4E-02	Gamma-glutamyl Amino Acid	Peptide
gamma-glutamyltryptophan	1.07	4.6E-02	Gamma-glutamyl Amino Acid	Peptide

Table S5. Xenobiotic metabolites associated with a vegan (relative to non-vegetarian) dietary pattern at FDR < 0.05 in linear regression models with SmartSVA approach.

Metabolite	Fold Change	FDR	Subclass
saccharin	2.89	<3.9E-05	Food Component/Plant
1,3,7-trimethylurate	2.07	<3.9E-05	Xanthine Metabolism
perfluorooctanoate (pfoa)	1.67	<3.9E-05	Chemical
3-methyl catechol sulfate (1)	3.11	<3.9E-05	Benzoate Metabolism
mannonate	1.36	<3.9E-05	Food Component/Plant
ibuprofen	3.40	<3.9E-05	Drug - Analgesics, Anesthetics
2-hydroxyacetaminophen sulfate	5.41	<3.9E-05	Drug - Analgesics, Anesthetics
sulfate of piperine metabolite c18h21no3 (3)	3.41	<3.9E-05	Food Component/Plant
4-acetaminophen sulfate	9.79	<3.9E-05	Drug - Analgesics, Anesthetics
3-methylxanthine	5.30	<3.9E-05	Xanthine Metabolism
glucuronide of piperine metabolite c17h21no3 (4)	4.06	<3.9E-05	Food Component/Plant
sulfate of piperine metabolite c18h21no3 (1)	3.89	<3.9E-05	Food Component/Plant
glucuronide of piperine metabolite c17h21no3 (5)	3.63	<3.9E-05	Food Component/Plant
7-methylxanthine	5.88	<3.9E-05	Xanthine Metabolism
theobromine	23.76	<3.9E-05	Xanthine Metabolism
1,3-dimethylurate	3.15	<3.9E-05	Xanthine Metabolism
glucuronide of piperine metabolite c17h21no3 (3)	3.68	<3.9E-05	Food Component/Plant
caffeine	8.80	<3.9E-05	Xanthine Metabolism
perfluorooctanesulfonate (pfos)	3.07	<3.9E-05	Chemical
piperine	8.56	<3.9E-05	Food Component/Plant
1-methylxanthine	3.47	<3.9E-05	Xanthine Metabolism
4-acetamidophenol	5.26	<3.9E-05	Drug - Analgesics, Anesthetics
5-acetylamino-6-formylamino-3-methyluracil	4.96	<3.9E-05	Xanthine Metabolism
sulfate of piperine metabolite c16h19no3 (3)	4.37	<3.9E-05	Food Component/Plant
1-methylurate	5.03	<3.9E-05	Xanthine Metabolism
sulfate of piperine metabolite c16h19no3 (2)	5.14	<3.9E-05	Food Component/Plant
5-acetylamino-6-amino-3-methyluracil	26.43	<3.9E-05	Xanthine Metabolism
1,7-dimethylurate	10.59	<3.9E-05	Xanthine Metabolism
theophylline	15.13	<3.9E-05	Xanthine Metabolism
paraxanthine	15.35	<3.9E-05	Xanthine Metabolism
3,5-dichloro-2,6-dihydroxybenzoic acid	2.60	<3.9E-05	Chemical
3-bromo-5-chloro-2,6-dihydroxybenzoic acid	4.45	<3.9E-05	Chemical
salicyluric glucuronide	2.58	5.6E-05	Drug - Analgesics, Anesthetics
4-allylcatechol sulfate	0.45	6.7E-05	Benzoate Metabolism
ergothioneine	0.62	7.0E-05	Food Component/Plant
indolin-2-one	1.50	1.0E-04	Food Component/Plant
2-piperidinone	1.79	1.0E-04	Food Component/Plant
catechol sulfate	0.57	1.2E-04	Benzoate Metabolism
6-hydroxyindole sulfate	1.57	1.4E-04	Chemical
ethyl beta-glucopyranoside	0.36	1.5E-04	Food Component/Plant
stachydrine	0.44	1.6E-04	Food Component/Plant
gluconate	1.31	1.7E-04	Food Component/Plant
4-acetylphenyl sulfate	0.31	2.6E-04	Benzoate Metabolism
methyl glucopyranoside (alpha + beta)	0.38	2.9E-04	Food Component/Plant
3-acetylphenol sulfate	1.53	3.1E-04	Chemical
homostachydrine	2.16	3.2E-04	Food Component/Plant
2,3-dihydroxypyridine	1.54	4.4E-04	Food Component/Plant
4-ethylphenyl sulfate	0.15	5.2E-04	Benzoate Metabolism
methyl indole-3-acetate	1.74	5.7E-04	Food Component/Plant
histidine betaine (hercynine)	0.66	5.7E-04	Food Component/Plant
3-indoleglyoxylic acid	1.21	1.2E-03	Food Component/Plant
thymol sulfate	2.06	1.3E-03	Food Component/Plant
cinnamoylglycine	0.55	1.7E-03	Food Component/Plant
4-acetylcatechol sulfate (1)	0.65	1.8E-03	Food Component/Plant
sulfate	1.12	2.1E-03	Chemical
3-methoxycatechol sulfate (2)	0.69	2.1E-03	Benzoate Metabolism
4-allylphenol sulfate	0.53	2.4E-03	Food Component/Plant
guaiacol sulfate	0.69	2.4E-03	Benzoate Metabolism
ectoine	1.79	2.4E-03	Chemical
3-formylindole	1.30	2.5E-03	Food Component/Plant
2-hydroxyhippurate (salicylurate)	1.83	2.5E-03	Benzoate Metabolism
4-acetamidobenzoate	0.64	2.7E-03	Chemical

4-ethylcatechol sulfate	0.59	3.5E-03	Benzoate Metabolism
p-cresol sulfate	1.49	6.0E-03	Benzoate Metabolism
quinate	2.32	6.5E-03	Food Component/Plant
3-hydroxyhippurate sulfate	1.38	9.0E-03	Benzoate Metabolism
3-hydroxypyridine sulfate	1.50	9.4E-03	Chemical
2-oxindole-3-acetate	0.62	9.8E-03	Food Component/Plant
daidzein sulfate (2)	0.52	9.8E-03	Food Component/Plant
dihydrocaffeate sulfate (2)	0.64	9.9E-03	Food Component/Plant
thioproline	0.93	2.0E-02	Chemical
2-acetamidophenol sulfate	0.61	2.1E-02	Food Component/Plant
3-(3-hydroxyphenyl)propionate	1.32	2.9E-02	Benzoate Metabolism
alliin	0.70	3.0E-02	Food Component/Plant
3-phenylpropionate (hydrocinnamate)	0.70	3.1E-02	Benzoate Metabolism
beta-guanidinopropanoate	1.15	3.2E-02	Food Component/Plant
o-sulfo-l-tyrosine	1.09	3.4E-02	Chemical
2,3-dihydroxyisovalerate	0.76	3.4E-02	Food Component/Plant
salicylate	1.44	4.1E-02	Drug - Topical Agents
o-cresol sulfate	1.30	4.2E-02	Benzoate Metabolism
(2,4 or 2,5)-dimethylphenol sulfate	1.36	4.3E-02	Food Component/Plant
hippurate	0.73	4.4E-02	Benzoate Metabolism
4-hydroxyhippurate	1.18	4.7E-02	Benzoate Metabolism
2,6-dihydroxybenzoic acid	0.79	4.9E-02	Drug - Topical Agents
2,2'-methylenebis(6-tert-butyl-p-cresol)	0.69	4.9E-02	Chemical

Table S6. Top 40 metabolites positively associated with a vegan dietary pattern at FDR < 0.05 in linear regression analysis without SmartSVA.

Metabolites	Fold Change	FDR	Subclass	Major Class
4-ethylphenyl sulfate	6.48	<1.0E-04	Benzoate Metabolism	Xenobiotics
s-methylmethionine	5.02	<1.0E-04	Methionine, Cysteine, SAM and Taurine Metabolism	Amino Acid
4-acetylphenyl sulfate	3.92	<1.0E-04	Benzoate Metabolism	Xenobiotics
branched chain 14:0 dicarboxylic acid	3.48	<1.0E-04	Fatty Acid, Dicarboxylate	Lipid
glycohyocholate	3.12	<1.0E-04	Secondary Bile Acid Metabolism	Lipid
ethyl beta-glucopyranoside	3.09	<1.0E-04	Food Component/Plant	Xenobiotics
methyl glucopyranoside (alpha + beta)	2.86	<1.0E-04	Food Component/Plant	Xenobiotics
stachydrine	2.73	1.4E-03	Food Component/Plant	Xenobiotics
4-allylcatechol sulfate	2.47	1.5E-03	Benzoate Metabolism	Xenobiotics
indolepropionate	2.28	<1.0E-04	Tryptophan Metabolism	Amino Acid
1-linoleoyl-2-linolenoyl-gpc (18:2/18:3)	2.26	<1.0E-04	Phosphatidylcholine (PC)	Lipid
beta-cryptoxanthin	2.26	<1.0E-04	Vitamin A Metabolism	Cofactors and Vitamins
2-acetamidophenol sulfate	2.26	5.9E-03	Food Component/Plant	Xenobiotics
cinnamoylglycine	2.24	1.2E-02	Food Component/Plant	Xenobiotics
n-methylproline	2.23	5.9E-03	Urea cycle; Arginine and Proline Metabolism	Amino Acid
glycochenodeoxycholate 3-sulfate	2.14	9.2E-03	Primary Bile Acid Metabolism	Lipid
catechol sulfate	2.13	4.4E-04	Benzoate Metabolism	Xenobiotics
daidzein sulfate (2)	2.07	2.1E-02	Food Component/Plant	Xenobiotics
pentose acid	2.04	3.7E-04	Partially Characterized Molecules	Partially Characterized Molecules
dihydrocaffeate sulfate (2)	2.03	1.3E-02	Food Component/Plant	Xenobiotics
s-methylcysteine sulfoxide	1.99	<1.0E-04	Methionine, Cysteine, SAM and Taurine Metabolism	Amino Acid
n-delta-acetylornithine	1.98	<1.0E-04	Urea cycle; Arginine and Proline Metabolism	Amino Acid
gentisate	1.98	5.3E-04	Tyrosine Metabolism	Amino Acid
octadecadienedioate (c18:2-dc)	1.96	<1.0E-04	Fatty Acid, Dicarboxylate	Lipid
glycocholate	1.95	4.6E-03	Primary Bile Acid Metabolism	Lipid
2-methylserine	1.94	<1.0E-04	Glycine, Serine and Threonine Metabolism	Amino Acid
n-linoleoylglycine	1.94	5.1E-04	Fatty Acid Metabolism (Acyl Glycine)	Lipid
4-methoxyphenol sulfate	1.93	3.4E-03	Tyrosine Metabolism	Amino Acid
4-acetylcatechol sulfate (1)	1.91	4.4E-03	Food Component/Plant	Xenobiotics
4-allylphenol sulfate	1.88	8.4E-03	Food Component/Plant	Xenobiotics
chiro-inositol	1.85	1.0E-02	Inositol Metabolism	Lipid
octadecenedioate (c18:1-dc)	1.83	<1.0E-04	Fatty Acid, Dicarboxylate	Lipid
1,2-dilinoleoyl-gpe (18:2/18:2)	1.81	2.3E-03	Phosphatidylethanolamine (PE)	Lipid
4-ethylcatechol sulfate	1.80	2.4E-02	Benzoate Metabolism	Xenobiotics
12,13-dihome	1.80	<1.0E-04	Fatty Acid, Dihydroxy	Lipid
glycochenodeoxycholate	1.79	6.8E-03	Primary Bile Acid Metabolism	Lipid
2-aminophenol sulfate	1.77	2.8E-02	Food Component/Plant	Xenobiotics
hippurate	1.76	1.8E-02	Benzoate Metabolism	Xenobiotics
histidine betaine (hercynine)	1.75	7.8E-04	Food Component/Plant	Xenobiotics
2-oxindole-3-acetate	1.75	8.5E-03	Food Component/Plant	Xenobiotics

Table S7. Top 40 metabolites inversely associated with a vegan dietary pattern at FDR < 0.05 in linear regression analysis without SmartSVA.

Metabolites	Fold Change	FDR	Subclass	Major Class
theobromine	0.05	<1.0E-04	Xanthine Metabolism	Xenobiotics
3-carboxy-4-methyl-5-propyl-2-furanpropanoate (cmpf)	0.06	<1.0E-04	Fatty Acid, Dicarboxylate	Lipid
5-acetylamino-6-amino-3-methyluracil	0.06	<1.0E-04	Xanthine Metabolism	Xenobiotics
3-methylhistidine	0.07	<1.0E-04	Histidine Metabolism	Amino Acid
1-methyl-5-imidazoleacetate	0.09	<1.0E-04	Histidine Metabolism	Amino Acid
paraxanthine	0.10	<1.0E-04	Xanthine Metabolism	Xenobiotics
theophylline	0.10	<1.0E-04	Xanthine Metabolism	Xenobiotics
hydroxy-cmpf	0.10	<1.0E-04	Fatty Acid, Dicarboxylate	Lipid
piperine	0.13	<1.0E-04	Food Component/Plant	Xenobiotics
4-acetaminophen sulfate	0.14	1.3E-02	Drug - Analgesics, Anesthetics	Xenobiotics
1,7-dimethylurate	0.14	<1.0E-04	Xanthine Metabolism	Xenobiotics
caffeine	0.16	<1.0E-04	Xanthine Metabolism	Xenobiotics
4-acetamidophenol	0.19	1.6E-03	Drug - Analgesics, Anesthetics	Xenobiotics
7-methylxanthine	0.20	<1.0E-04	Xanthine Metabolism	Xenobiotics
sulfate of piperine metabolite c16h19no3 (2)	0.22	<1.0E-04	Food Component/Plant	Xenobiotics
3-methylxanthine	0.23	<1.0E-04	Xanthine Metabolism	Xenobiotics
3-bromo-5-chloro-2,6-dihydroxybenzoic acid	0.25	<1.0E-04	Chemical	Xenobiotics
2-hydroxyacetaminophen sulfate	0.25	3.1E-02	Drug - Analgesics, Anesthetics	Xenobiotics
sulfate of piperine metabolite c16h19no3 (3)	0.25	<1.0E-04	Food Component/Plant	Xenobiotics
1-methylurate	0.26	<1.0E-04	Xanthine Metabolism	Xenobiotics
5-acetylamino-6-formylamino-3-methyluracil	0.27	<1.0E-04	Xanthine Metabolism	Xenobiotics
n,n,n-trimethyl-5-aminovalerate	0.27	<1.0E-04	Lysine Metabolism	Amino Acid
sulfate of piperine metabolite c18h21no3 (1)	0.27	<1.0E-04	Food Component/Plant	Xenobiotics
heptenedioate (c7:1-dc)	0.28	<1.0E-04	Fatty Acid, Dicarboxylate	Lipid
(14 or 15)-methylpalmitate (a17:0 or i17:0)	0.30	<1.0E-04	Fatty Acid, Branched	Lipid
glucuronide of piperine metabolite c17h21no3 (4)	0.30	2.4E-04	Food Component/Plant	Xenobiotics
perfluorooctanesulfonate (pfos)	0.31	<1.0E-04	Chemical	Xenobiotics
sulfate of piperine metabolite c18h21no3 (3)	0.31	<1.0E-04	Food Component/Plant	Xenobiotics
(12 or 13)-methylmyristate (a15:0 or i15:0)	0.32	<1.0E-04	Fatty Acid, Branched	Lipid
glucuronide of piperine metabolite c17h21no3 (3)	0.32	1.3E-04	Food Component/Plant	Xenobiotics
glucuronide of piperine metabolite c17h21no3 (5)	0.33	1.9E-04	Food Component/Plant	Xenobiotics
tridecenedioate (c13:1-dc)	0.35	<1.0E-04	Fatty Acid, Dicarboxylate	Lipid
salicyluric glucuronide	0.35	1.5E-02	Drug - Analgesics, Anesthetics	Xenobiotics
1-methylxanthine	0.36	<1.0E-04	Xanthine Metabolism	Xenobiotics
1-margaroylglycerol (17:0)	0.36	<1.0E-04	Monoacylglycerol	Lipid
sphingomyelin (d18:1/25:0, d19:0/24:1, d20:1/23:0, d19:1/24:0)	0.39	<1.0E-04	Sphingomyelins	Lipid
(16 or 17)-methylstearate (a19:0 or i19:0)	0.41	<1.0E-04	Fatty Acid, Branched	Lipid
1,3-dimethylurate	0.41	<1.0E-04	Xanthine Metabolism	Xenobiotics
3,5-dichloro-2,6-dihydroxybenzoic acid	0.41	<1.0E-04	Chemical	Xenobiotics
saccharin	0.42	4.0E-03	Food Component/Plant	Xenobiotics

Table S8. Amino acid metabolites associated with a vegan (relative to non-vegetarian) dietary pattern at FDR < 0.05 in linear regression analysis without SmartSVA.

Metabolites	Fold Change	FDR	Subclass
s-methylmethionine	5.02	<1.0E-04	Methionine, Cysteine, SAM and Taurine Metabolism
indolepropionate	2.28	<1.0E-04	Tryptophan Metabolism
n-methylproline	2.23	5.9E-03	Urea cycle; Arginine and Proline Metabolism
s-methylcysteine sulfoxide	1.99	<1.0E-04	Methionine, Cysteine, SAM and Taurine Metabolism
n-delta-acetylornithine	1.98	<1.0E-04	Urea cycle; Arginine and Proline Metabolism
gentisate	1.98	5.3E-04	Tyrosine Metabolism
2-methylserine	1.94	<1.0E-04	Glycine, Serine and Threonine Metabolism
4-methoxyphenol sulfate	1.93	3.4E-03	Tyrosine Metabolism
2,3-dihydroxy-2-methylbutyrate	1.69	<1.0E-04	Leucine, Isoleucine and Valine Metabolism
tryptophan betaine	1.67	2.8E-02	Tryptophan Metabolism
n2,n5-diacetylornithine	1.67	4.8E-04	Urea cycle; Arginine and Proline Metabolism
pyroglutamine	1.64	<1.0E-04	Glutamate Metabolism
s-methylcysteine	1.61	4.2E-04	Methionine, Cysteine, SAM and Taurine Metabolism
hypotaurine	1.40	3.2E-03	Methionine, Cysteine, SAM and Taurine Metabolism
1-ribosyl-imidazoleacetate	1.37	3.6E-04	Histidine Metabolism
dopamine 3-o-sulfate	1.33	3.3E-02	Tyrosine Metabolism
n-acetylglycine	1.30	1.3E-02	Glycine, Serine and Threonine Metabolism
carboxyethyl-gaba	1.27	2.2E-03	Glutamate Metabolism
glycine	1.26	2.4E-03	Glycine, Serine and Threonine Metabolism
prolylhydroxyproline	1.25	2.2E-02	Urea cycle; Arginine and Proline Metabolism
asparagine	1.19	<1.0E-04	Alanine and Aspartate Metabolism
n-acetyl-isoputreanine	1.17	3.6E-02	Polyamine Metabolism
n-acetylputrescine	1.17	4.3E-02	Polyamine Metabolism
betaine	1.17	6.4E-03	Glycine, Serine and Threonine Metabolism
glutamine	1.16	2.2E-02	Glutamate Metabolism
5-methylthioribose	1.09	2.9E-02	Methionine, Cysteine, SAM and Taurine Metabolism
isoleucine	0.94	3.6E-02	Leucine, Isoleucine and Valine Metabolism
cysteine sulfinic acid	0.90	1.7E-03	Methionine, Cysteine, SAM and Taurine Metabolism
5-methylthioadenosine (mta)	0.89	2.8E-02	Polyamine Metabolism
lysine	0.89	8.0E-03	Lysine Metabolism
2,3-dihydroxy-5-methylthio-4-pentenoate (dmtpa)	0.88	1.9E-02	Methionine, Cysteine, SAM and Taurine Metabolism
kynurenine	0.87	2.1E-02	Tryptophan Metabolism
3-methyl-2-oxobutyrate	0.86	4.8E-02	Leucine, Isoleucine and Valine Metabolism
5-(galactosylhydroxy)-l-lysine	0.82	3.0E-02	Lysine Metabolism
valine	0.81	1.4E-02	Leucine, Isoleucine and Valine Metabolism
kynurenate	0.80	2.2E-02	Tryptophan Metabolism
homoarginine	0.79	1.2E-02	Urea cycle; Arginine and Proline Metabolism
dimethylglycine	0.79	3.5E-02	Glycine, Serine and Threonine Metabolism
2-aminobutyrate	0.78	3.0E-04	Glutathione Metabolism
hydantoin-5-propionate	0.78	3.5E-02	Histidine Metabolism
glutamate	0.78	2.8E-02	Glutamate Metabolism
1-carboxyethylphenylalanine	0.77	3.4E-02	Phenylalanine Metabolism
3-hydroxyisobutyrate	0.77	2.2E-02	Leucine, Isoleucine and Valine Metabolism
urea	0.76	6.1E-04	Urea cycle; Arginine and Proline Metabolism
2-hydroxy-4-(methylthio)butanoic acid	0.73	2.3E-02	Methionine, Cysteine, SAM and Taurine Metabolism
isovalerylglycine	0.73	4.5E-02	Leucine, Isoleucine and Valine Metabolism
n2-acetyllysine	0.72	1.9E-02	Lysine Metabolism
1-carboxyethylvaline	0.72	2.0E-02	Leucine, Isoleucine and Valine Metabolism
3-hydroxy-2-ethylpropionate	0.72	2.4E-03	Leucine, Isoleucine and Valine Metabolism
2-hydroxyphenylacetate	0.72	5.4E-03	Phenylalanine Metabolism
1-carboxyethylisoleucine	0.70	2.0E-02	Leucine, Isoleucine and Valine Metabolism
hydroxyproline	0.69	<1.0E-04	Urea cycle; Arginine and Proline Metabolism
1-carboxyethyltyrosine	0.69	2.5E-02	Tyrosine Metabolism
3-indoxyl sulfate	0.69	9.4E-03	Tryptophan Metabolism
n-acetylcitrulline	0.68	3.4E-02	Urea cycle; Arginine and Proline Metabolism
n-acetyltyrosine	0.68	9.6E-03	Tyrosine Metabolism
8-methoxykynurenate	0.68	1.3E-02	Tryptophan Metabolism
2-hydroxybutyrate/2-hydroxyisobutyrate	0.68	1.0E-03	Glutathione Metabolism
isobutyrylcarnitine (c4)	0.67	5.4E-03	Leucine, Isoleucine and Valine Metabolism
glutarylcarnitine (c5-dc)	0.67	1.0E-02	Lysine Metabolism
indole-3-carboxylate	0.66	7.9E-03	Tryptophan Metabolism
imidazole propionate	0.66	3.2E-02	Histidine Metabolism

n-formylanthranilic acid	0.65	4.2E-03	Tryptophan Metabolism
anthranilate	0.64	2.1E-02	Tryptophan Metabolism
1-methylhistidine	0.64	<1.0E-04	Histidine Metabolism
isovalerylcarnitine (c5)	0.63	8.4E-04	Leucine, Isoleucine and Valine Metabolism
tiglyl carnitine (c5)	0.62	<1.0E-04	Leucine, Isoleucine and Valine Metabolism
homocitrulline	0.62	3.5E-04	Urea cycle; Arginine and Proline Metabolism
n-acetyl-1-methylhistidine	0.62	7.9E-03	Histidine Metabolism
2-oxoarginine	0.61	7.0E-03	Urea cycle; Arginine and Proline Metabolism
6-oxopiperidine-2-carboxylate	0.60	2.7E-03	Lysine Metabolism
4-hydroxyglutamate	0.60	1.2E-02	Glutamate Metabolism
beta-hydroxyisovaleroylcarnitine	0.60	<1.0E-04	Leucine, Isoleucine and Valine Metabolism
creatine	0.59	<1.0E-04	Creatine Metabolism
2-methylbutyrylcarnitine (c5)	0.59	<1.0E-04	Leucine, Isoleucine and Valine Metabolism
n-carbamoylalanine	0.58	7.9E-03	Alanine and Aspartate Metabolism
formiminoglutamate	0.55	<1.0E-04	Histidine Metabolism
2-aminoadipate	0.55	<1.0E-04	Lysine Metabolism
xanthurenate	0.54	7.6E-04	Tryptophan Metabolism
indoleacetylglutamine	0.51	3.5E-03	Tryptophan Metabolism
n,n,n-trimethyl-5-aminovalerate	0.27	<1.0E-04	Lysine Metabolism
1-methyl-5-imidazoleacetate	0.09	<1.0E-04	Histidine Metabolism
3-methylhistidine	0.07	<1.0E-04	Histidine Metabolism

Table S9. Lipid metabolites associated with a vegan (relative to non-vegetarian) dietary pattern at FDR < 0.05 in linear regression analysis without SmartSVA approach.

Metabolites	Fold Change	FDR	Subclass
branched chain 14:0 dicarboxylic acid	3.48	<1.0E-04	Fatty Acid, Dicarboxylate
glycohyocholate	3.12	<1.0E-04	Secondary Bile Acid Metabolism
1-linoleoyl-2-linolenoyl-gpc (18:2/18:3)	2.26	<1.0E-04	Phosphatidylcholine (PC)
glycochenodeoxycholate 3-sulfate	2.14	9.2E-03	Primary Bile Acid Metabolism
octadecadienedioate (c18:2-dc)	1.96	<1.0E-04	Fatty Acid, Dicarboxylate
glycocholate	1.95	4.6E-03	Primary Bile Acid Metabolism
n-linoleoylglycine	1.94	5.1E-04	Fatty Acid Metabolism (Acyl Glycine)
chiro-inositol	1.85	1.0E-02	Inositol Metabolism
octadecenedioate (c18:1-dc)	1.83	<1.0E-04	Fatty Acid, Dicarboxylate
1,2-dilinoleoyl-gpe (18:2/18:2)	1.81	2.3E-03	Phosphatidylethanolamine (PE)
12,13-dihome	1.80	<1.0E-04	Fatty Acid, Dihydroxy
glycochenodeoxycholate	1.79	6.8E-03	Primary Bile Acid Metabolism
dodecenedioate (c12:1-dc)	1.75	6.0E-03	Fatty Acid, Dicarboxylate
1-lignoceroyl-gpc (24:0)	1.74	<1.0E-04	Lysophospholipid
2-butenoylglycine	1.73	4.1E-03	Fatty Acid Metabolism (Acyl Glycine)
1-cerotoyl-gpc (26:0)	1.63	<1.0E-04	Lysophospholipid
trans-2-hexenoylglycine	1.62	3.3E-03	Fatty Acid Metabolism (Acyl Glycine)
3-hydroxybutyroylglycine	1.60	5.1E-03	Fatty Acid Metabolism (Acyl Glycine)
octadecenedioylcarnitine (c18:1-dc)	1.59	3.3E-03	Fatty Acid Metabolism (Acyl Carnitine, Dicarboxylate)
cis-4-decenoate (10:1n6)	1.58	1.1E-03	Medium Chain Fatty Acid
3-hydroxydodecanedioate	1.57	2.2E-02	Fatty Acid, Dicarboxylate
dodecanedioate (c12)	1.56	6.4E-03	Fatty Acid, Dicarboxylate
n-linoleoyltaurine	1.55	6.7E-03	Endocannabinoid
1-palmitoleoyl-2-linolenoyl-gpc (16:1/18:3)	1.53	1.1E-02	Phosphatidylcholine (PC)
9,10-dihome	1.52	4.0E-03	Fatty Acid, Dihydroxy
3-hydroxysebacate	1.51	1.8E-02	Fatty Acid, Monohydroxy
dodecadienoate (12:2)	1.50	5.5E-04	Fatty Acid, Dicarboxylate
(2 or 3)-decenoate (10:1n7 or n8)	1.48	4.0E-03	Medium Chain Fatty Acid
2-hydroxysebacate	1.45	6.0E-03	Fatty Acid, Dicarboxylate
1-oleoyl-2-linoleoyl-gpe (18:1/18:2)	1.44	6.0E-03	Phosphatidylethanolamine (PE)
1,2-dilinoleoyl-gpc (18:2/18:2)	1.43	<1.0E-04	Phosphatidylcholine (PC)
linolenoylcarnitine (c18:3)	1.42	5.0E-04	Fatty Acid Metabolism (Acyl Carnitine, Polyunsaturated)
beta-sitosterol	1.41	1.6E-02	Sterol
tetradecadienoate (14:2)	1.40	1.9E-02	Long Chain Polyunsaturated Fatty Acid (n3 and n6)
lactosyl-n-nervonoyl-sphingosine (d18:1/24:1)	1.40	<1.0E-04	Lactosylceramides (LCER)
linoleoyl-linolenoyl-glycerol (18:2/18:3) [2]	1.39	4.1E-02	Diacylglycerol
linolenate (18:3n3 or 3n6)	1.39	2.9E-02	Long Chain Polyunsaturated Fatty Acid (n3 and n6)
17alpha-hydroxypregnenolone 3-sulfate	1.38	3.1E-02	Pregnenolone Steroids
glycosyl ceramide (d18:2/24:1, d18:1/24:2)	1.37	8.8E-04	Hexosylceramides (HCER)
1-linoleoyl-gpe (18:2)	1.37	2.2E-02	Lysophospholipid
2-hydroxydecanoate	1.34	1.7E-02	Fatty Acid, Monohydroxy
2-aminoheptanoate	1.33	1.3E-03	Fatty Acid, Amino
1-linolenoyl-gpc (18:3)	1.32	2.2E-03	Lysophospholipid
1-oleoyl-gpe (18:1)	1.30	2.2E-02	Lysophospholipid
1-oleoyl-2-arachidonoyl-gpe (18:1/20:4)	1.30	1.5E-02	Phosphatidylethanolamine (PE)
1-stearoyl-2-linoleoyl-gpi (18:0/18:2)	1.30	2.3E-03	Phosphatidylinositol (PI)
3-hydroxydecanoate	1.29	2.6E-02	Fatty Acid, Monohydroxy
sphingomyelin (d18:2/24:2)	1.28	4.6E-04	Sphingomyelins
1-linoleoyl-gpc (18:2)	1.27	1.8E-02	Lysophospholipid
1-linoleoyl-gpi (18:2)	1.26	1.7E-02	Lysophospholipid
1-oleoyl-gpc (18:1)	1.25	1.3E-03	Lysophospholipid
3beta,7alpha-dihydroxy-5-cholestenoate	1.25	4.3E-02	Sterol
sphingomyelin (d18:2/24:1, d18:1/24:2)	1.23	<1.0E-04	Sphingomyelins
1-linoleoyl-2-arachidonoyl-gpc (18:2/20:4n6)	1.23	6.4E-03	Phosphatidylcholine (PC)
malonate	1.22	1.9E-02	Fatty Acid Synthesis
3beta-hydroxy-5-cholestenoate	1.20	2.0E-02	Sterol
lactosyl-n-palmitoyl-sphingosine (d18:1/16:0)	1.20	2.6E-03	Lactosylceramides (LCER)
2-hydroxynervonate	1.20	2.8E-02	Fatty Acid, Monohydroxy
myo-inositol	1.18	1.8E-02	Inositol Metabolism
palmitoyl-sphingosine-phosphoethanolamine (d18:1/16:0)	1.18	2.7E-03	Ceramide PEs
lignoceroyl sphingomyelin (d18:1/24:0)	1.16	1.3E-02	Sphingomyelins

sphingosine 1-phosphate	1.16	2.2E-02	Sphingosines
sphingomyelin (d18:1/24:1, d18:2/24:0)	1.14	1.4E-03	Sphingomyelins
palmitoyl dihydrosphingomyelin (d18:0/16:0)	1.13	2.8E-02	Dihydrosphingomyelins
glycerophosphorylcholine (gpc)	1.12	2.4E-02	Phospholipid Metabolism
1-palmitoyl-2-dihomo-linolenoyl-gpc (16:0/20:3n3 or 6)	1.08	2.2E-02	Phosphatidylcholine (PC)
1-stearoyl-2-linoleoyl-gpc (18:0/18:2)	1.08	2.1E-02	Phosphatidylcholine (PC)
sphingomyelin (d18:1/20:0, d16:1/22:0)	0.91	2.2E-02	Sphingomyelins
3,4-dihydroxybutyrate	0.88	2.2E-02	Fatty Acid, Dihydroxy
behenoyl sphingomyelin (d18:1/22:0)	0.86	8.6E-03	Sphingomyelins
sphingomyelin (d18:2/21:0, d16:2/23:0)	0.86	2.8E-02	Sphingomyelins
1-palmitoyl-2-docosahexaenoyl-gpc (16:0/22:6)	0.85	6.9E-03	Phosphatidylcholine (PC)
sphingomyelin (d18:2/14:0, d18:1/14:1)	0.84	4.1E-02	Sphingomyelins
2-hydroxyglutarate	0.84	7.3E-03	Fatty Acid, Dicarboxylate
sphingomyelin (d18:1/18:1, d18:2/18:0)	0.82	8.0E-04	Sphingomyelins
stearate (18:0)	0.82	2.7E-02	Long Chain Saturated Fatty Acid
1-(1-enyl-palmitoyl)-gpe (p-16:0)	0.81	1.4E-02	Lysoplasmalogen
arachidoylcarnitine (c20)	0.80	2.8E-02	Fatty Acid Metabolism (Acyl Carnitine, Long Chain Saturated)
palmitate (16:0)	0.80	3.1E-02	Long Chain Saturated Fatty Acid
hexanoylcarnitine (c6)	0.80	2.7E-02	Fatty Acid Metabolism (Acyl Carnitine, Medium Chain)
palmitoylcarnitine (c16)	0.80	4.1E-03	Fatty Acid Metabolism (Acyl Carnitine, Long Chain Saturated)
palmitoyl ethanolamide	0.80	7.4E-03	Endocannabinoid
sphingomyelin (d18:2/18:1)	0.78	9.5E-03	Sphingomyelins
1-stearoyl-2-docosahexaenoyl-gpc (18:0/22:6)	0.78	5.7E-03	Phosphatidylcholine (PC)
n-behenoyl-sphingadienine (d18:2/22:0)	0.78	1.0E-02	Ceramides
myristoyl dihydrosphingomyelin (d18:0/14:0)	0.77	5.6E-04	Dihydrosphingomyelins
stearoyl sphingomyelin (d18:1/18:0)	0.77	<1.0E-04	Sphingomyelins
1-palmitoyl-2-palmitoleoyl-gpc (16:0/16:1)	0.77	2.0E-02	Phosphatidylcholine (PC)
stearoylcarnitine (c18)	0.76	5.4E-03	Fatty Acid Metabolism (Acyl Carnitine, Long Chain Saturated)
1-palmitoyl-gpg (16:0)	0.76	4.4E-02	Lysophospholipid
adrenate (22:4n6)	0.76	2.4E-02	Long Chain Polyunsaturated Fatty Acid (n3 and n6)
1-palmitoyl-2-docosahexaenoyl-gpe (16:0/22:6)	0.75	3.7E-02	Phosphatidylethanolamine (PE)
n-stearoyltaurine	0.75	1.3E-02	Endocannabinoid
sphingomyelin (d18:2/23:1)	0.75	<1.0E-04	Sphingomyelins
myristoleate (14:1n5)	0.75	3.2E-02	Long Chain Monounsaturated Fatty Acid
carnitine	0.74	1.4E-02	Carnitine Metabolism
ceramide (d18:1/20:0, d16:1/22:0, d20:1/18:0)	0.74	3.5E-04	Ceramides
1-palmitoylglycerol (16:0)	0.74	2.6E-02	Monoacylglycerol
cortolone glucuronide (1)	0.74	8.7E-03	Corticosteroids
1-(1-enyl-palmitoyl)-2-arachidonoyl-gpc (p-16:0/20:4)	0.73	<1.0E-04	Plasmalogen
sphingomyelin (d18:0/20:0, d16:0/22:0)	0.73	1.9E-03	Dihydrosphingomyelins
1-(1-enyl-stearoyl)-2-linoleoyl-gpe (p-18:0/18:2)	0.73	1.8E-04	Plasmalogen
erucoylcarnitine (c22:1)	0.73	3.5E-02	Fatty Acid Metabolism (Acyl Carnitine, Monounsaturated)
butyrylcarnitine (c4)	0.73	4.4E-02	Fatty Acid Metabolism (also BCAA Metabolism)
1-pentadecanoylglycerol (15:0)	0.72	3.5E-03	Monoacylglycerol
1-(1-enyl-palmitoyl)-2-linoleoyl-gpe (p-16:0/18:2)	0.70	<1.0E-04	Plasmalogen
nonanoylcarnitine (c9)	0.70	3.2E-03	Fatty Acid Metabolism (Acyl Carnitine, Medium Chain)
myristoylcarnitine (c14)	0.70	1.6E-03	Fatty Acid Metabolism (Acyl Carnitine, Long Chain Saturated)
sphingomyelin (d18:1/21:0, d17:1/22:0, d16:1/23:0)	0.69	<1.0E-04	Sphingomyelins
trimethylamine n-oxide	0.69	6.1E-04	Phospholipid Metabolism
sphingomyelin (d18:1/14:0, d16:1/16:0)	0.69	<1.0E-04	Sphingomyelins
palmitoleate (16:1n7)	0.69	2.3E-02	Long Chain Monounsaturated Fatty Acid
docosahexaenoate (dha; 22:6n3)	0.68	5.8E-03	Long Chain Polyunsaturated Fatty Acid (n3 and n6)
sphingomyelin (d18:1/17:0, d17:1/18:0, d19:1/16:0)	0.67	<1.0E-04	Sphingomyelins
pentadecanoate (15:0)	0.67	<1.0E-04	Long Chain Saturated Fatty Acid
1-docosahexaenoylglycerol (22:6)	0.66	2.8E-02	Monoacylglycerol
1-(1-enyl-stearoyl)-2-oleoyl-gpe (p-18:0/18:1)	0.66	<1.0E-04	Plasmalogen
1-stearoyl-2-docosahexaenoyl-gpe (18:0/22:6)	0.66	7.0E-03	Phosphatidylethanolamine (PE)
1-palmitoleoylglycerol (16:1)	0.66	2.8E-02	Monoacylglycerol
sphingomyelin (d17:1/16:0, d18:1/15:0, d16:1/17:0)	0.65	<1.0E-04	Sphingomyelins
5alpha-androstan-3alpha,17beta-diol monosulfate (1)	0.65	3.5E-02	Androgenic Steroids
behenate (22:0)	0.64	1.5E-02	Long Chain Saturated Fatty Acid
sphingomyelin (d18:1/19:0, d19:1/18:0)	0.64	<1.0E-04	Sphingomyelins

1-myristoyl-2-palmitoyl-gpc (14:0/16:0)	0.64	3.9E-03	Phosphatidylcholine (PC)
1-(1-enyl-stearoyl)-gpe (p-18:0)	0.64	<1.0E-04	Lysoplasmalogen
2r,3r-dihydroxybutyrate	0.64	<1.0E-04	Fatty Acid, Dihydroxy
myristate (14:0)	0.63	5.1E-04	Long Chain Saturated Fatty Acid
docosapentaenoate (n6 dpa; 22:5n6)	0.63	1.2E-03	Long Chain Polyunsaturated Fatty Acid (n3 and n6)
1-stearoyl-gpg (18:0)	0.62	2.4E-04	Lysophospholipid
linoleoyl-docosahexaenoyl-glycerol (18:2/22:6) [2]	0.61	1.7E-02	Diacylglycerol
1-(1-enyl-palmitoyl)-2-arachidonoyl-gpe (p-16:0/20:4)	0.61	<1.0E-04	Plasmalogen
margarate (17:0)	0.61	1.3E-04	Long Chain Saturated Fatty Acid
n-stearoyl-sphingadienine (d18:2/18:0)	0.60	1.9E-04	Ceramides
glutarate (c5-dc)	0.60	3.3E-03	Fatty Acid, Dicarboxylate
1-myristoylglycerol (14:0)	0.59	7.9E-03	Monoacylglycerol
propionylcarnitine (c3)	0.58	1.1E-03	Fatty Acid Metabolism (also BCAA Metabolism)
picolinoylglycine	0.58	<1.0E-04	Fatty Acid Metabolism (Acyl Glycine)
sphingomyelin (d18:0/18:0, d19:0/17:0)	0.58	<1.0E-04	Dihydrosphingomyelins
ceramide (d16:1/24:1, d18:1/22:1)	0.58	<1.0E-04	Ceramides
10-undecenoate (11:1n1)	0.56	<1.0E-04	Medium Chain Fatty Acid
sphingomyelin (d17:1/14:0, d16:1/15:0)	0.56	<1.0E-04	Sphingomyelins
n-stearoyl-sphingosine (d18:1/18:0)	0.56	<1.0E-04	Ceramides
docosahexaenoylcarnitine (c22:6)	0.56	1.9E-04	Fatty Acid Metabolism (Acyl Carnitine, Polyunsaturated)
3-carboxy-4-methyl-5-pentyl-2-furanpropionate (3-cmpfp)	0.56	<1.0E-04	Fatty Acid, Dicarboxylate
1-(1-enyl-stearoyl)-2-arachidonoyl-gpe (p-18:0/20:4)	0.56	<1.0E-04	Plasmalogen
2-hydroxyadipate	0.56	2.2E-03	Fatty Acid, Dicarboxylate
10-nonadecenoate (19:1n9)	0.56	1.9E-04	Long Chain Monounsaturated Fatty Acid
sphingomyelin (d17:2/16:0, d18:2/15:0)	0.55	<1.0E-04	Sphingomyelins
isoursodeoxycholate	0.53	3.6E-02	Secondary Bile Acid Metabolism
ceramide (d18:1/14:0, d16:1/16:0)	0.53	<1.0E-04	Ceramides
n-palmitoyl-heptadecasphingosine (d17:1/16:0)	0.49	<1.0E-04	Ceramides
10-heptadecenoate (17:1n7)	0.49	<1.0E-04	Long Chain Monounsaturated Fatty Acid
n-stearoyl-sphinganine (d18:0/18:0)	0.44	<1.0E-04	Dihydroceramides
ceramide (d18:1/17:0, d17:1/18:0)	0.43	<1.0E-04	Ceramides
undecenoylcarnitine (c11:1)	0.43	<1.0E-04	Fatty Acid Metabolism (Acyl Carnitine, Monounsaturated)
margaroylcarnitine (c17)	0.43	<1.0E-04	Fatty Acid Metabolism (Acyl Carnitine, Long Chain Saturated)
(16 or 17)-methylstearate (a19:0 or i19:0)	0.41	<1.0E-04	Fatty Acid, Branched
sphingomyelin (d18:1/25:0, d19:0/24:1, d20:1/23:0, d19:1/24:0)	0.39	<1.0E-04	Sphingomyelins
1-margaroylglycerol (17:0)	0.36	<1.0E-04	Monoacylglycerol
tridecenedioate (c13:1-dc)	0.35	<1.0E-04	Fatty Acid, Dicarboxylate
(12 or 13)-methylmyristate (a15:0 or i15:0)	0.32	<1.0E-04	Fatty Acid, Branched
(14 or 15)-methylpalmitate (a17:0 or i17:0)	0.30	<1.0E-04	Fatty Acid, Branched
heptenedioate (c7:1-dc)	0.28	<1.0E-04	Fatty Acid, Dicarboxylate
hydroxy-cmpf	0.10	<1.0E-04	Fatty Acid, Dicarboxylate
3-carboxy-4-methyl-5-propyl-2-furanpropanoate (cmpf)	0.06	<1.0E-04	Fatty Acid, Dicarboxylate

Table S10. Carbohydrate, cofactors/vitamins, energy, nucleotide, partially characterized, and peptide metabolites associated with a vegan dietary pattern in linear regression analysis without SmartSVA.

Metabolites	Fold Change	FDR	Subclass	Major Class
ribulonate/xylulonate/lyxonate	1.25	4.3E-02	Pentose Metabolism	Carbohydrate
glucuronate	0.83	3.8E-02	Aminosugar Metabolism	Carbohydrate
mannose	0.80	3.3E-02	Fructose, Mannose and Galactose Metabolism	Carbohydrate
pyruvate	0.78	2.3E-02	Glycolysis, Gluconeogenesis, and Pyruvate Metabolism	Carbohydrate
beta-cryptoxanthin	2.26	<1.0E-04	Vitamin A Metabolism	Cofactors and Vitamins
carotene diol (2)	1.56	4.5E-04	Vitamin A Metabolism	Cofactors and Vitamins
carotene diol (1)	1.47	1.1E-03	Vitamin A Metabolism	Cofactors and Vitamins
carotene diol (3)	1.38	3.7E-02	Vitamin A Metabolism	Cofactors and Vitamins
threonate	1.30	1.1E-03	Ascorbate and Aldarate Metabolism	Cofactors and Vitamins
oxalate (ethanedioate)	1.30	1.9E-03	Ascorbate and Aldarate Metabolism	Cofactors and Vitamins
n1-methyl-2-pyridone-5-carboxamide	0.73	4.3E-02	Nicotinate and Nicotinamide Metabolism	Cofactors and Vitamins
quinolinate	0.66	6.5E-04	Nicotinate and Nicotinamide Metabolism	Cofactors and Vitamins
isocitrate	1.35	9.8E-04	TCA Cycle	Energy
citrate	1.19	2.2E-02	TCA Cycle	Energy
alpha-ketoglutarate	0.81	1.4E-02	TCA Cycle	Energy
2-methylcitrate/homocitrate	0.79	4.7E-03	TCA Cycle	Energy
cytosine	1.55	4.3E-02	Pyrimidine Metabolism, Cytidine containing	Nucleotide
n2,n2-dimethylguanosine	0.91	4.8E-02	Purine Metabolism, Guanine containing	Nucleotide
n-acetyl-beta-alanine	0.86	1.6E-02	Pyrimidine Metabolism, Uracil containing	Nucleotide
5,6-dihydrothymine	0.85	3.2E-02	Pyrimidine Metabolism, Thymine containing	Nucleotide
beta-alanine	0.83	9.9E-04	Pyrimidine Metabolism, Uracil containing	Nucleotide
orotidine	0.82	1.7E-03	Pyrimidine Metabolism, Orotate containing	Nucleotide
pentose acid	2.04	3.7E-04	Partially Characterized Molecules	Partially Characterized
branched-chain, straight-chain, or cyclopropyl 10:1 fatty acid (1)	0.75	2.8E-02	Partially Characterized Molecules	Partially Characterized
glutamine conjugate of c7h12o2	0.50	4.7E-03	Partially Characterized Molecules	Partially Characterized
gamma-glutamylglycine	1.40	<1.0E-04	Gamma-glutamyl Amino Acid	Peptide
gamma-glutamylglutamine	1.37	2.4E-02	Gamma-glutamyl Amino Acid	Peptide
gamma-glutamylhistidine	1.13	2.9E-02	Gamma-glutamyl Amino Acid	Peptide
gamma-glutamylphenylalanine	0.87	4.5E-02	Gamma-glutamyl Amino Acid	Peptide
gamma-glutamyltyrosine	0.84	2.1E-02	Gamma-glutamyl Amino Acid	Peptide
gamma-glutamylleucine	0.84	1.0E-02	Gamma-glutamyl Amino Acid	Peptide
gamma-glutamylvaline	0.81	2.7E-02	Gamma-glutamyl Amino Acid	Peptide
phenylacetylglutamate	0.65	5.9E-03	Acetylated Peptides	Peptide
phenylacetylcarnitine	0.43	1.5E-03	Acetylated Peptides	Peptide

Table S11. Xenobiotic metabolites associated with a vegan (relative to non-vegetarian) dietary pattern at FDR < 0.05 in linear regression analysis without SmartSVA.

Metabolites	Fold Change	FDR	Subclass
4-ethylphenyl sulfate	6.48	<1.0E-04	Benzoate Metabolism
4-acetylphenyl sulfate	3.92	<1.0E-04	Benzoate Metabolism
ethyl beta-glucopyranoside	3.09	<1.0E-04	Food Component/Plant
methyl glucopyranoside (alpha + beta)	2.86	<1.0E-04	Food Component/Plant
stachydrine	2.73	1.4E-03	Food Component/Plant
4-allylcatechol sulfate	2.47	1.5E-03	Benzoate Metabolism
2-acetamidophenol sulfate	2.26	5.9E-03	Food Component/Plant
cinnamoylglycine	2.24	1.2E-02	Food Component/Plant
catechol sulfate	2.13	4.4E-04	Benzoate Metabolism
daidzein sulfate (2)	2.07	2.1E-02	Food Component/Plant
dihydrocaffeate sulfate (2)	2.03	1.3E-02	Food Component/Plant
4-acetylcatechol sulfate (1)	1.91	4.4E-03	Food Component/Plant
4-allylphenol sulfate	1.88	8.4E-03	Food Component/Plant
4-ethylcatechol sulfate	1.80	2.4E-02	Benzoate Metabolism
2-aminophenol sulfate	1.77	2.8E-02	Food Component/Plant
hippurate	1.76	1.8E-02	Benzoate Metabolism
histidine betaine (hercynine)	1.75	7.8E-04	Food Component/Plant
2-oxindole-3-acetate	1.75	8.5E-03	Food Component/Plant
guaiacol sulfate	1.74	6.7E-03	Benzoate Metabolism
2,6-dihydroxybenzoic acid	1.60	6.7E-03	Drug - Topical Agents
3-methoxycatechol sulfate (2)	1.59	5.7E-03	Benzoate Metabolism
2,3-dihydroxyisovalerate	1.58	2.7E-02	Food Component/Plant
4-acetamidobenzoate	1.58	8.5E-03	Chemical
ergothioneine	1.57	2.3E-03	Food Component/Plant
tartronate (hydroxymalonate)	1.19	3.1E-02	Food Component/Plant
gluconate	0.80	1.6E-02	Food Component/Plant
mannonate	0.78	1.3E-03	Food Component/Plant
indolin-2-one	0.73	2.6E-02	Food Component/Plant
2,3-dihydroxypyridine	0.71	2.1E-02	Food Component/Plant
6-hydroxyindole sulfate	0.71	1.7E-02	Chemical
methyl indole-3-acetate	0.66	2.8E-02	Food Component/Plant
2-piperidinone	0.62	5.1E-03	Food Component/Plant
homostachydrine	0.61	4.0E-02	Food Component/Plant
perfluorooctanoate (pfoa)	0.59	<1.0E-04	Chemical
1,3,7-trimethylurate	0.57	2.3E-03	Xanthine Metabolism
3-methyl catechol sulfate (1)	0.42	2.3E-03	Benzoate Metabolism
saccharin	0.42	4.0E-03	Food Component/Plant
3,5-dichloro-2,6-dihydroxybenzoic acid	0.41	<1.0E-04	Chemical
1,3-dimethylurate	0.41	<1.0E-04	Xanthine Metabolism
1-methylxanthine	0.36	<1.0E-04	Xanthine Metabolism
salicyluric glucuronide	0.35	1.5E-02	Drug - Analgesics, Anesthetics
glucuronide of piperine metabolite c17h21no3 (5)	0.33	1.9E-04	Food Component/Plant
glucuronide of piperine metabolite c17h21no3 (3)	0.32	1.3E-04	Food Component/Plant
sulfate of piperine metabolite c18h21no3 (3)	0.31	<1.0E-04	Food Component/Plant
perfluorooctanesulfonate (pfos)	0.31	<1.0E-04	Chemical
glucuronide of piperine metabolite c17h21no3 (4)	0.30	2.4E-04	Food Component/Plant
sulfate of piperine metabolite c18h21no3 (1)	0.27	<1.0E-04	Food Component/Plant
5-acetylamino-6-formylamino-3-methyluracil	0.27	<1.0E-04	Xanthine Metabolism
1-methylurate	0.26	<1.0E-04	Xanthine Metabolism
sulfate of piperine metabolite c16h19no3 (3)	0.25	<1.0E-04	Food Component/Plant
2-hydroxyacetaminophen sulfate	0.25	3.1E-02	Drug - Analgesics, Anesthetics
3-bromo-5-chloro-2,6-dihydroxybenzoic acid	0.25	<1.0E-04	Chemical
3-methylxanthine	0.23	<1.0E-04	Xanthine Metabolism
sulfate of piperine metabolite c16h19no3 (2)	0.22	<1.0E-04	Food Component/Plant
7-methylxanthine	0.20	<1.0E-04	Xanthine Metabolism
4-acetamidophenol	0.19	1.6E-03	Drug - Analgesics, Anesthetics
caffeine	0.16	<1.0E-04	Xanthine Metabolism
1,7-dimethylurate	0.14	<1.0E-04	Xanthine Metabolism
4-acetaminophen sulfate	0.14	1.3E-02	Drug - Analgesics, Anesthetics
piperine	0.13	<1.0E-04	Food Component/Plant
theophylline	0.10	<1.0E-04	Xanthine Metabolism
paraxanthine	0.10	<1.0E-04	Xanthine Metabolism

5-acetylamino-6-amino-3-methyluracil	0.06	<1.0E-04	Xanthine Metabolism
theobromine	0.05	<1.0E-04	Xanthine Metabolism

Table 12. Metabolite subclasses associated with diet group (vegan vs non-vegetarian) at FDR < 0.05 without SVA method.

Subclass Labels	Fold Change (95% CI)	FDR	n significant metabolites	#↑	# ↓	n total metabolites
Inositol Metabolism	1.50 (1.19, 1.89)	1.5E-02	2	0	2	2
Vitamin A Metabolism	1.35 (1.18, 1.54)	<1.2E-05	4	0	4	6
Lactosylceramides (LCER)	1.18 (1.06, 1.32)	4.4E-02	2	0	2	3
Lysophospholipid	1.15 (1.04, 1.27)	4.4E-02	10	2	8	32
Sterol	1.14 (1.04, 1.25)	4.6E-02	3	0	3	7
Alanine and Aspartate Metabolism	0.94 (0.86, 1.02)	4.8E-02	2	1	1	9
Fatty Acid, Dicarboxylate	0.90 (0.79, 1.03)	4.8E-02	16	8	8	34
Purine Metabolism, Guanine containing	0.88 (0.74, 1.03)	4.3E-02	1	1	0	3
Pyrimidine Metabolism, Orotate containing	0.88 (0.78, 0.99)	1.2E-02	1	1	0	4
Tryptophan Metabolism	0.88 (0.78, 0.98)	7.3E-03	11	9	2	20
Creatine Metabolism	0.85 (0.79, 0.92)	1.8E-05	1	1	0	3
Leucine, Isoleucine and Valine Metabolism	0.84 (0.75, 0.95)	2.2E-03	14	13	1	32
Sphingomyelins	0.84 (0.78, 0.91)	1.2E-05	20	16	4	29
Monoacylglycerol	0.84 (0.67, 1.04)	3.8E-02	6	6	0	17
Lysine Metabolism	0.84 (0.76, 0.92)	2.0E-04	7	7	0	18
Chemical	0.83 (0.75, 0.93)	5.8E-04	6	5	1	20
Lysoplasmalogen	0.82 (0.71, 0.94)	2.2E-03	2	2	0	4
Phenylalanine Metabolism	0.81 (0.69, 0.95)	3.6E-03	2	2	0	7
Plasmalogen	0.80 (0.74, 0.86)	<1.2E-05	6	6	0	11
Dihydrosphingomyelins	0.79 (0.69, 0.91)	3.5E-04	4	3	1	5
Carnitine Metabolism	0.78 (0.62, 1.00)	1.7E-02	1	1	0	2
Long Chain Saturated Fatty Acid	0.75 (0.64, 0.89)	3.2E-04	6	6	0	8
Long Chain Monounsaturated Fatty Acid	0.75 (0.59, 0.94)	4.6E-03	4	4	0	7
Fatty Acid Metabolism (Acyl Carnitine, Long Chain Saturated)	0.74 (0.64, 0.86)	2.9E-05	5	5	0	8
Ceramides	0.66 (0.58, 0.76)	<1.2E-05	8	8	0	11
Acetylated Peptides	0.65 (0.50, 0.85)	6.7E-04	2	2	0	4
Dihydroceramides	0.63 (0.52, 0.77)	<1.2E-05	1	1	0	2
Histidine Metabolism	0.63 (0.56, 0.71)	<1.2E-05	8	7	1	15
Fatty Acid, Branched	0.34 (0.27, 0.42)	<1.2E-05	3	3	0	3
Drug - Analgesics, Anesthetics	0.29 (0.13, 0.61)	5.7E-04	4	4	0	5
Xanthine Metabolism	0.19 (0.12, 0.30)	<1.2E-05	13	13	0	13

Table S13. Component metabolites of each subclass associated with a vegan (relative to non-vegetarian) dietary pattern at FDR < 0.05.

Subclass	Metabolites
Vitamin A Metabolism	beta-cryptoxanthin; carotene diol (2); carotene diol (1); carotene diol (3); retinal; retinol (vitamin a)
Ketone Bodies	3-hydroxybutyrate (bhba); acetoacetate
Inositol Metabolism	chiro-inositol; myo-inositol
Fatty Acid Metabolism (Acyl Glycine)	trans-2-hexenoylglycine; n-linoleoylglycine; 3-hydroxybutyroylglycine; 2-butenoylglycine; picolinoylglycine
Lactosylceramides (LCER)	lactosyl-n-nervonoyl-sphingosine (d18:1/24:1); lactosyl-n-palmitoyl-sphingosine (d18:1/16:0)
Benzoate Metabolism	4-ethylphenyl sulfate; 4-acetylphenyl sulfate; catechol sulfate; 4-allylcatechol sulfate; 3-methoxycatechol sulfate (2); guaiacol sulfate; 4-ethylcatechol sulfate; 3-phenylpropionate (hydrocinnamate); hippurate; 4-hydroxyhippurate; o-cresol sulfate; 3-(3-hydroxyphenyl)propionate; 3-hydroxyhippurate sulfate; p-cresol sulfate; 2-hydroxyhippurate (salicylurate); 3-methyl catechol sulfate (1)
Aminosugar Metabolism	erythronate; glucuronate
Glutamate Metabolism	glutamine; pyroglutamine; carboxyethyl-gaba; alpha-ketoglutaramate; beta-citrylglutamate; 4-hydroxyglutamate; n-acetylglutamate; glutamate
Fructose, Mannose and Galactose Metabolism	galactonate; fructose
Partially Characterized Molecules	pentose acid; glutamine conjugate of c6h10o2 (2); branched-chain, straight-chain, or cyclopropyl 10:1 fatty acid (3); glycine conjugate of c10h14o2 (1); branched-chain, straight-chain, or cyclopropyl 10:1 fatty acid (1); glycine conjugate of c10h12o2; carnitine of c10h14o2 (5); glutamine conjugate of c7h12o2
Pantothenate and CoA Metabolism	pantoate
Diacylglycerol	oleoyl-arachidonoyl-glycerol (18:1/20:4) [1]; oleoyl-arachidonoyl-glycerol (18:1/20:4) [2]; linoleoyl-docosahexaenoyl-glycerol (18:2/22:6) [2]; palmitoleoyl-linoleoyl-glycerol (16:1/18:2) [1]; palmitoyl-linoleoyl-glycerol (16:0/18:2) [2]; palmitoyl-linoleoyl-glycerol (16:0/18:2) [1]
Urea cycle; Arginine and Proline Metabolism	n-delta-acetylornithine; n-methylproline; n2,n5-diacetylornithine; prolylhydroxyproline; homoarginine; 3-amino-2-piperidone; proline; n-acetylproline; n-acetylcitrulline; n-acetylarginine; 2-oxoarginine; homocitrulline; urea; hydroxyproline
Sphingolipid Synthesis	sphingadienine; sphinganine
Fatty Acid, Dicarboxylate	3-hydroxydodecanedioate; octadecenedioate (c18:1-dc); branched chain 14:0 dicarboxylic acid; dodecenedioate (c12:1-dc); dodecadienoate (12:2); octadecadienedioate (c18:2-dc); dodecanedioate (c12); tetradecadienedioate (c14:2-dc); sebacate (c10-dc); 2-hydroxysebacate; hexadecanedioate (c16); 3-hydroxyadipate; 3-methyladipate; maleate; 2-hydroxyglutarate; 2-hydroxyadipate; glutarate (c5-dc); 3-carboxy-4-methyl-5-pentyl-2-furanpropionate (3-cmpfp); hydroxy-cmpf; tridecenedioate (c13:1-dc); 3-carboxy-4-methyl-5-propyl-2-furanpropanoate (cmpf); heptenedioate (c7:1-dc)
Tyrosine Metabolism	gentisate; 4-methoxyphenol sulfate; tyramine o-sulfate; 4-hydroxyphenylacetatoylcarnitine; vanillactate; 3-(4-hydroxyphenyl)lactate (hpla); n-formylphenylalanine; tyrosine; p-cresol glucuronide; n-acetyltyrosine; 1-carboxyethyltyrosine
Long Chain Polyunsaturated Fatty Acid (n3 and n6)	tetradecadienoate (14:2); linolenate (18:3n3 or 3n6); docosadienoate (22:2n6); hexadecadienoate (16:2n6); dihomolinolenate (20:3n3 or 3n6); docosapentaenoate (dpa; 22:5n3); nisinate (24:6n3); docosatrienoate (22:3n6); eicosapentaenoate (epa; 20:5n3); adrenate (22:4n6); docosahexaenoate (dha; 22:6n3); docosapentaenoate (n6 dpa; 22:5n6)
Guanidino and Acetamido Metabolism	4-guanidinobutanoate; guanidinosuccinate
Secondary Bile Acid Metabolism	glycohyocholate; 3b-hydroxy-5-cholenoic acid; deoxycholate; glycoursodeoxycholic acid sulfate (1); taurodeoxycholic acid 3-sulfate; lithocholate sulfate (1); deoxycholic acid glucuronide; taurodeoxycholate; ursodeoxycholate; isoursodeoxycholate; deoxycholic acid 12-sulfate
Fatty Acid Metabolism (also BCAA Metabolism)	2-methylmalonylcarnitine (c4-dc); propionylglycine (c3); butyrylcarnitine (c4); propionylcarnitine (c3)
Purine Metabolism, Adenine containing	1-methyladenosine; n6-carbamoylthreonyladenosine; adenine
Phospholipid Metabolism	phosphocholine; glycerophosphoethanolamine; choline; trimethylamine n-oxide
Pyrimidine Metabolism, Uracil containing	uracil; pseudouridine; 3-(3-amino-3-carboxypropyl)uridine; n-acetyl-beta-alanine; beta-alanine
Pyrimidine Metabolism, Orotate containing	orotate; n-carbamoylaspartate; orotidine
Dihydrosphingomyelins	palmitoyl dihydrosphingomyelin (d18:0/16:0); behenoyl dihydrosphingomyelin (d18:0/22:0); sphingomyelin (d18:0/20:0, d16:0/22:0); myristoyl dihydrosphingomyelin (d18:0/14:0); sphingomyelin (d18:0/18:0, d19:0/17:0)
Creatine Metabolism	guanidinoacetate; creatinine; creatine

Alanine and Aspartate Metabolism	asparagine; alanine; hydroxyasparagine; n-acetylalanine; n-carbamoylalanine; aspartate
Glycerolipid Metabolism	glycerol 3-phosphate; glycerophosphoglycerol; glycerol
Purine Metabolism, Guanine containing	guanosine; 7-methylguanine; n2,n2-dimethylguanosine
Purine Metabolism, (Hypo)Xanthine/Inosine containing	allantoin; hypoxanthine; urate; n1-methylinosine; xanthosine
Acetylated Peptides	phenylacetylglutamine; phenylacetylcarnitine; 4-hydroxyphenylacetylglutamine; phenylacetylglutamate
Sphingomyelins	sphingomyelin (d18:2/24:1, d18:1/24:2); sphingomyelin (d18:2/24:2); sphingomyelin (d18:1/24:1, d18:2/24:0); lignoceroyl sphingomyelin (d18:1/24:0); sphingomyelin (d18:1/20:2, d18:2/20:1, d16:1/22:2); sphingomyelin (d18:1/22:2, d18:2/22:1, d16:1/24:2); sphingomyelin (d18:1/20:0, d16:1/22:0); sphingomyelin (d18:2/21:0, d16:2/23:0); behenoyl sphingomyelin (d18:1/22:0); sphingomyelin (d18:2/14:0, d18:1/14:1); sphingomyelin (d18:1/18:1, d18:2/18:0); sphingomyelin (d18:2/18:1); stearoyl sphingomyelin (d18:1/18:0); sphingomyelin (d18:2/23:1); sphingomyelin (d18:1/21:0, d17:1/22:0, d16:1/23:0); sphingomyelin (d18:1/19:0, d19:1/18:0); sphingomyelin (d18:1/14:0, d16:1/16:0); sphingomyelin (d18:1/17:0, d17:1/18:0, d19:1/16:0); sphingomyelin (d18:1/25:0, d19:0/24:1, d20:1/23:0, d19:1/24:0); sphingomyelin (d17:1/14:0, d16:1/15:0); sphingomyelin (d17:2/16:0, d18:2/15:0); sphingomyelin (d17:1/16:0, d18:1/15:0, d16:1/17:0)
Dihydroceramides	n-palmitoyl-sphinganine (d18:0/16:0); n-stearoyl-sphinganine (d18:0/18:0)
Monoacylglycerol	2-palmitoylglycerol (16:0); 2-oleoylglycerol (18:1); 1-oleoylglycerol (18:1); 1-dihomo-linolenylglycerol (20:3); 2-palmitoleoylglycerol (16:1); 1-docosahexaenoylglycerol (22:6); 1-palmitoylglycerol (16:0); 1-pentadecanoylglycerol (15:0); 1-palmitoleoylglycerol (16:1); 1-myristoylglycerol (14:0); 1-margaroylglycerol (17:0)
Lysine Metabolism	fructosyllysine; 5-hydroxylysine; hydroxy-n6,n6,n6-trimethyllysine; 5-(galactosylhydroxy)-l-lysine; glutarylcarnitine (c5-dc); n2-acetyllysine; lysine; 6-oxopiperidine-2-carboxylate; n6-acetyllysine; 2-aminoadipate; n,n,n-trimethyl-5-aminovalerate
Tryptophan Metabolism	indolepropionate; tryptophan betaine; 6-bromotryptophan; indolebutyrate; indoleacetate; n-acetyltryptophan; 8-methoxykynurenate; indolelactate; kynurenine; c-glycosyltryptophan; 3-indoxyl sulfate; kynurenate; indoleacetylglutamine; n-formylanthranilic acid; indole-3-carboxylate; anthranilate; xanthurenate
Food Component/Plant	methyl glucopyranoside (alpha + beta); ethyl beta-glucopyranoside; ergothioneine; stachydrine; histidine betaine (hercynine); cinnamoylglycine; 4-acetylcatechol sulfate (1); 4-allylphenol sulfate; dihydrocaffeate sulfate (2); daidzein sulfate (2); 2-oxindole-3-acetate; 2-acetamidophenol sulfate; alliin; 2,3-dihydroxyisovalerate; (2,4 or 2,5)-dimethylphenol sulfate; beta-guanidinopropanoate; quinate; 3-formylindole; thymol sulfate; 3-indoleglyoxylic acid; methyl indole-3-acetate; 2,3-dihydroxypyridine; homostachydrine; gluconate; indolin-2-one; 2-piperidinone; saccharin; mannonate; sulfate of piperine metabolite c18h21no3 (3); glucuronide of piperine metabolite c17h21no3 (4); sulfate of piperine metabolite c18h21no3 (1); glucuronide of piperine metabolite c17h21no3 (5); glucuronide of piperine metabolite c17h21no3 (3); piperine; sulfate of piperine metabolite c16h19no3 (3); sulfate of piperine metabolite c16h19no3 (2)
Fatty Acid Metabolism (Acyl Carnitine, Long Chain Saturated)	cerotoylcarnitine (c26); behenoylcarnitine (c22); arachidoylcarnitine (c20); lignoceroylcarnitine (c24); stearoylcarnitine (c18); myristoylcarnitine (c14); palmitoylcarnitine (c16); margaroylcarnitine (c17)
Lysoplasmalogen	1-(1-enyl-oleoyl)-gpe (p-18:1); 1-(1-enyl-palmitoyl)-gpc (p-16:0); 1-(1-enyl-palmitoyl)-gpe (p-16:0); 1-(1-enyl-stearoyl)-gpe (p-18:0)
Plasmalogen	1-(1-enyl-palmitoyl)-2-oleoyl-gpc (p-16:0/18:1); 1-(1-enyl-palmitoyl)-2-arachidonoyl-gpc (p-16:0/20:4); 1-(1-enyl-palmitoyl)-2-linoleoyl-gpe (p-16:0/18:2); 1-(1-enyl-stearoyl)-2-linoleoyl-gpe (p-18:0/18:2); 1-(1-enyl-stearoyl)-2-oleoyl-gpe (p-18:0/18:1); 1-(1-enyl-palmitoyl)-2-arachidonoyl-gpe (p-16:0/20:4); 1-(1-enyl-stearoyl)-2-arachidonoyl-gpe (p-18:0/20:4)
Long Chain Monounsaturated Fatty Acid	myristoleate (14:1n5); palmitoleate (16:1n7); 10-nonadecenoate (19:1n9); 10-heptadecenoate (17:1n7)
Ceramides	n-palmitoyl-sphingadienine (d18:2/16:0); n-palmitoyl-sphingosine (d18:1/16:0); n-behenoyl-sphingadienine (d18:2/22:0); ceramide (d18:1/20:0, d16:1/22:0, d20:1/18:0); n-stearoyl-sphingadienine (d18:2/18:0); ceramide (d16:1/24:1, d18:1/22:1); n-stearoyl-sphingosine (d18:1/18:0); ceramide (d18:1/14:0, d16:1/16:0); ceramide (d18:1/17:0, d17:1/18:0); n-palmitoyl-heptadecasphingosine (d17:1/16:0)
Chemical	4-acetamidobenzoate; thioproline; 2,2'-methylenebis(6-tert-butyl-p-cresol); o-sulfo-l-tyrosine; 3-hydroxypyridine sulfate; ectoine; sulfate; 3-acetylphenol sulfate; 6-hydroxyindole sulfate; perfluorooctanoate (pfoa); perfluorooctanesulfonate (pfos); 3,5-dichloro-2,6-dihydroxybenzoic acid; 3-bromo-5-chloro-2,6-dihydroxybenzoic acid
Leucine, Isoleucine and Valine Metabolism	2,3-dihydroxy-2-methylbutyrate; n-acetylisoleucine; 3-methylglutarylcarnitine (2); alpha-hydroxyisocaproate; 3-hydroxyisobutyrate; isobutyrylglycine (c4); 3-methyl-2-oxobutyrate; alpha-hydroxyisovalerate; 2-hydroxy-3-methylvalerate; isobutyrylcarnitine (c4); leucine; isoleucine; beta-hydroxyisovalerate; isovalerylcarnitine (c5); 3-hydroxy-2-

	ethylpropionate; isovalerylglycine; valine; 1-carboxyethylleucine; 2-methylbutyryl-carnitine (c5); tiglyl carnitine (c5); beta-hydroxyisovaleroylcarnitine; 1-carboxyethyl-valine; n-acetylvaline; 1-carboxyethylisoleucine
Long Chain Saturated Fatty Acid	nonadecanoate (19:0); behenate (22:0); stearate (18:0); palmitate (16:0); myristate (14:0); margarate (17:0); pentadecanoate (15:0)
Phenylalanine Metabolism	phenylacetate; phenyllactate (pla); phenylalanine; n-acetylphenylalanine; 2-hydroxy-phenylacetate; 1-carboxyethylphenylalanine
Drug - Analgesics, Anesthetics	salicyluric glucuronide; ibuprofen; 2-hydroxyacetaminophen sulfate; 4-acetaminophen sulfate; 4-acetamidophenol
Xanthine Metabolism	1,3,7-trimethylurate; 3-methylxanthine; 7-methylxanthine; theobromine; 1,3-dime-thylurate; caffeine; 1-methylxanthine; 5-acetylamino-6-formylamino-3-methyluracil; 1-methylurate; 5-acetylamino-6-amino-3-methyluracil; 1,7-dimethylurate; theophylline; paraxanthine
Histidine Metabolism	1-ribosyl-imidazoleacetate; n-acetylhistidine; n-acetylcarnosine; hydantoin-5-propio-nate; imidazole lactate; n-acetyl-1-methylhistidine; imidazole propionate; formimino-glutamate; 1-methylhistidine; 3-methylhistidine; 1-methyl-5-imidazoleacetate
Fatty Acid, Branched	(16 or 17)-methylstearate (a19:0 or i19:0); (12 or 13)-methylmyristate (a15:0 or i15:0); (14 or 15)-methylpalmitate (a17:0 or i17:0)

Table S14. Metabolites differentially abundant (FDR < 0.05) in linear regression analysis with full sample that were nondifferential post cross-validation[1].

Metabolite	Subclass
hippurate	Benzoate Metabolism
guanidinoacetate	Creatine Metabolism
2,6-dihydroxybenzoic acid	Drug - Topical Agents
glyco-beta-muricholate	Primary Bile Acid Metabolism
2-palmitoylglycerol (16:0)	Monoacylglycerol
glycerol 3-phosphate	Glycerolipid Metabolism
glycochenodeoxycholate glucuronide (1)	Primary Bile Acid Metabolism
4-hydroxyhippurate	Benzoate Metabolism
galactonate	Fructose, Mannose and Galactose Metabolism
gamma-glutamyltryptophan	Gamma-glutamyl Amino Acid
deoxycholate	Secondary Bile Acid Metabolism
o-cresol sulfate	Benzoate Metabolism
(2,4 or 2,5)-dimethylphenol sulfate	Food Component/Plant
gamma-tocopherol/beta-tocopherol	Tocopherol Metabolism
salicylate	Drug - Topical Agents

[1]T-scores calculated from residual regression variances obtained from cross-validation for each metabolite.

Table S15. Subclasses differential after cross-validation that were not differential in regression analysis with entire sample.

Pregnenolone Steroids
Fatty Acid Metabolism (Acyl Carnitine, Monounsaturated)
Carnitine Metabolism

Table 16. Metabolites or metabolite subclasses showing differential abundance between vegans and non-vegetarians (FDR < 0.05) in > 50% of bootstrapped linear regression analyses, but not differential in non-bootstrapped regression analysis[1].

Differential Metabolites (Associated Subclass)
xylose (Pentose Metabolism)
campesterol (Sterol)
glycodeoxycholate (Secondary Bile Acid Metabolism)
**Differential Subclasses**
Pregnenolone Steroids
Carnitine Metabolism

[1]Assessment of type II error to identify metabolites potentially misclassified as nondifferential. Metabolites differentially abundant in linear regression with full sample compared with metabolites significant in >50% of bootstrap samples.

Table S17. Numbers of differential metabolites or metabolite subclasses (at FDR < 0.05) in regression analysis with bootstrap sampling.

% of bootstrap samples	# significant metabolites
50	≥604
70	≥579
90	≥538
100	≥170
**% of bootstrap samples**	**# significant subclasses**
50	≥49
70	≥45
90	≥33
100	≥12

Table S18. List of 129 metabolites showing differential abundance (FDR < 0.05) in at least 90% of bootstrap regressions.

Metabolite	% of bootstrap samples showing significance
4-ethylphenyl sulfate	90
octadecenedioate (c18:1-dc)	90
dimethylglycine	90
n1-methylinosine	90
2-oxoarginine	90
2-hydroxyphenylacetate	90
myristoylcarnitine (c14)	90
palmitoylcarnitine (c16)	90
gamma-glutamylvaline	90
sulfate of piperine metabolite c18h21no3 (3)	90
adenine	91
n-stearoyl-sphingadienine (d18:2/18:0)	91
1,3,7-trimethylurate	91
1-(1-enyl-palmitoyl)-gpe (p-16:0)	91
1-(1-enyl-palmitoyl)-2-arachidonoyl-gpc (p-16:0/20:4)	91
1-linoleoyl-2-linolenoyl-gpc (18:2/18:3)	92
myristoleate (14:1n5)	92
stearoylcarnitine (c18)	92
gamma-glutamylphenylalanine	92
sphingomyelin (d18:2/23:1)	92
ceramide (d16:1/24:1, d18:1/22:1)	92
palmitoleate (16:1n7)	92
2-methylbutyrylcarnitine (c5)	92
glutamine	93
2-methylcitrate/homocitrate	93
anthranilate	93
perfluorooctanoate (pfoa)	93
methionine sulfoxide	93
stearoyl sphingomyelin (d18:1/18:0)	94
palmitoyl ethanolamide	94
1-pentadecanoylglycerol (15:0)	94
docosapentaenoate (n6 dpa; 22:5n6)	94
stearate (18:0)	94
tiglyl carnitine (c5)	94
beta-cryptoxanthin	95
glycohyocholate	95
sphingomyelin (d18:0/18:0, d19:0/17:0)	95
1-carboxyethylleucine	95
ibuprofen	95
1-carboxyethyltyrosine	95
homocitrulline	95
10-undecenoate (11:1n1)	95
sphingomyelin (d18:1/21:0, d17:1/22:0, d16:1/23:0)	95
sulfate of piperine metabolite c18h21no3 (1)	95
3-methyl catechol sulfate (1)	96
propionylcarnitine (c3)	96
1-palmitoleoylglycerol (16:1)	96
6-oxopiperidine-2-carboxylate	96
1-carboxyethylvaline	96
1-(1-enyl-stearoyl)-2-linoleoyl-gpe (p-18:0/18:2)	96
n-acetylvaline	96
formiminoglutamate	96
2-hydroxyacetaminophen sulfate	96
1-stearoyl-gpg (18:0)	96
3-carboxy-4-methyl-5-pentyl-2-furanpropionate (3-cmpfp)	96
1-(1-enyl-stearoyl)-2-oleoyl-gpe (p-18:0/18:1)	96
1-myristoylglycerol (14:0)	96
glucuronide of piperine metabolite c17h21no3 (5)	96
theobromine	96
1-lignoceroyl-gpc (24:0)	97
creatine	97

n-stearoyl-sphinganine (d18:0/18:0)	97
urea	97
1-(1-enyl-palmitoyl)-2-linoleoyl-gpe (p-16:0/18:2)	97
n-stearoyl-sphingosine (d18:1/18:0)	97
3-methylxanthine	97
1,3-dimethylurate	97
gamma-glutamylglutamine	98
beta-hydroxyisovaleroylcarnitine	98
palmitate (16:0)	98
ceramide (d18:1/14:0, d16:1/16:0)	98
1-carboxyethylphenylalanine	98
1-carboxyethylisoleucine	98
glucuronide of piperine metabolite c17h21no3 (4)	98
7-methylxanthine	98
1-(1-enyl-palmitoyl)-2-arachidonoyl-gpe (p-16:0/20:4)	98
4-acetaminophen sulfate	99
n6-acetyllysine	99
glucuronide of piperine metabolite c17h21no3 (3)	99
picolinoylglycine	99
perfluorooctanesulfonate (pfos)	99
1-methylxanthine	99
2-aminoadipate	99
1-methylurate	99
5-acetylamino-6-amino-3-methyluracil	99
2,3-dihydroxy-5-methylthio-4-pentenoate (dmtpa)	100
2r,3r-dihydroxybutyrate	100
hydroxyproline	100
sphingomyelin (d18:1/19:0, d19:1/18:0)	100
caffeine	100
1-methylhistidine	100
hydroxy-cmpf	100
piperine	100
4-acetamidophenol	100
5-acetylamino-6-formylamino-3-methyluracil	100
ceramide (d18:1/17:0, d17:1/18:0)	100
sphingomyelin (d18:1/14:0, d16:1/16:0)	100
1-(1-enyl-stearoyl)-gpe (p-18:0)	100
myristate (14:0)	100
sulfate of piperine metabolite c16h19no3 (3)	100
1-(1-enyl-stearoyl)-2-arachidonoyl-gpe (p-18:0/20:4)	100
undecenoylcarnitine (c11:1)	100
sulfate of piperine metabolite c16h19no3 (2)	100
tridecenedioate (c13:1-dc)	100
1-margaroylglycerol (17:0)	100
sphingomyelin (d18:1/17:0, d17:1/18:0, d19:1/16:0)	100
10-nonadecenoate (19:1n9)	100
sphingomyelin (d18:1/25:0, d19:0/24:1, d20:1/23:0, d19:1/24:0)	100
sphingomyelin (d17:1/14:0, d16:1/15:0)	100
n-palmitoyl-heptadecasphingosine (d17:1/16:0)	100
sphingomyelin (d17:2/16:0, d18:2/15:0)	100
3-carboxy-4-methyl-5-propyl-2-furanpropanoate (cmpf)	100
1,7-dimethylurate	100
sphingomyelin (d17:1/16:0, d18:1/15:0, d16:1/17:0)	100
theophylline	100
(16 or 17)-methylstearate (a19:0 or i19:0)	100
paraxanthine	100
margarate (17:0)	100
heptenedioate (c7:1-dc)	100
3,5-dichloro-2,6-dihydroxybenzoic acid	100
pentadecanoate (15:0)	100
10-heptadecenoate (17:1n7)	100
(12 or 13)-methylmyristate (a15:0 or i15:0)	100
n,n,n-trimethyl-5-aminovalerate	100
margaroylcarnitine (c17)	100
3-methylhistidine	100
1-methyl-5-imidazoleacetate	100

3-bromo-5-chloro-2,6-dihydroxybenzoic acid	100
(14 or 15)-methylpalmitate (a17:0 or i17:0)	100

Table S19. Metabolite subclasses showing differential abundance (FDR < 0.05) in at least 90% of bootstrap regressions.

Subclass	% of bootstrap samples showing significance
Fatty Acid Metabolism (also BCAA Metabolism)	90
Phospholipid Metabolism	91
Purine Metabolism, Adenine containing	94
Dihydrosphingomyelins	94
Ketone Bodies	95
Vitamin A Metabolism	97
Pyrimidine Metabolism, Uracil containing	97
Creatine Metabolism	97
Glycerolipid Metabolism	97
Purine Metabolism, (Hypo)Xanthine/Inosine containing	97
Alanine and Aspartate Metabolism	98
Purine Metabolism, Guanine containing	99
Acetylated Peptides	99
Sphingomyelins	100
Dihydroceramides	100
Monoacylglycerol	100
Lysine Metabolism	100
Tryptophan Metabolism	100
Food Component/Plant	100
Fatty Acid Metabolism (Acyl Carnitine, Long Chain Saturated)	100
Lysoplasmalogen	100
Plasmalogen	100
Long Chain Monounsaturated Fatty Acid	100
Ceramides	100
Chemical	100
Leucine, Isoleucine and Valine Metabolism	100
Long Chain Saturated Fatty Acid	100
Phenylalanine Metabolism	100
Drug - Analgesics, Anesthetics	100
Xanthine Metabolism	100
Histidine Metabolism	100
Fatty Acid, Branched	100

Table S20. Top components from principal components analysis and most influential metabolites [1,2].

PC	% Variance Explained	Cumulative % Variance	Metabolites (Loadings > 0.5)[2]
PC1	10.96	10.96	margaroylcarnitine (c17), (14 or 15)-methylpalmitate (a17:0 or i17:0), pentadecanoate (15:0), n-palmitoyl-heptadecasphingosine (d17:1/16:0), palmitoylcarnitine (c16), ceramide (d18:1/17:0, d17:1/18:0), (12 or 13)-methylmyristate (a15:0 or i15:0), 1-stearoyl-gpg (18:0), myristate (14:0), 1-methylhistidine, stearoylcarnitine (c18), 10-heptadecenoate (17:1n7), margarate (17:0), heptenedioate (c7:1-dc), n-stearoyl-sphingosine (d18:1/18:0), ceramide (d18:1/20:0, d16:1/22:0, d20:1/18:0), gamma-glutamylvaline, valine, myristoylcarnitine (c14), tridecenedioate (c13:1-dc), n-stearoyl-sphinganine (d18:0/18:0), sphingomyelin (d17:2/16:0, d18:2/15:0), n-stearoyl-sphingadienine (d18:2/18:0), 10-nonadecenoate (19:1n9), n-behenoyl-sphingadienine (d18:2/22:0), palmitoyl ethanolamide, 1-carboxyethylleucine, (16 or 17)-methylstearate (a19:0 or i19:0), 1-margaroylglycerol (17:0), palmitoyl-linoleoyl-glycerol (16:0/18:2) [2], palmitoyl-linoleoyl-glycerol (16:0/18:2) [1], 1-palmitoylglycerol (16:0), n,n,n-trimethyl-5-aminovalerate, sphingomyelin (d18:1/21:0, d17:1/22:0, d16:1/23:0), ceramide (d18:1/14:0, d16:1/16:0), 2r,3r-dihydroxybutyrate, 3-methylhistidine, 1-(1-enyl-stearoyl)-gpe (p-18:0), 2,3-dihydroxy-5-methylthio-4-pentenoate (dmtpa), 1-myristoylglycerol (14:0), picolinoylglycine, 3-bromo-5-chloro-2,6-dihydroxybenzoic acid, 3,5-dichloro-2,6-dihydroxybenzoic acid, 1,7-dimethylurate, 1-carboxyethylvaline, palmitate (16:0), 1-methyl-5-imidazoleacetate, theophylline, sphingomyelin (d18:1/19:0, d19:1/18:0), cortolone glucuronide (1), 1-(1-enyl-stearoyl)-2-arachidonoyl-gpe (p-18:0/20:4), gamma-glutamylleucine, stearate (18:0), sphingomyelin (d18:1/25:0, d19:0/24:1, d20:1/23:0, d19:1/24:0), 8-methoxykynurenate, sphingomyelin (d18:1/14:0, d16:1/16:0), undecenoylcarnitine (c11:1), n-acetylvaline, formiminoglutamate, 1-pentadecanoylglycerol (15:0), 1-(1-enyl-palmitoyl)-gpe (p-16:0), stearoyl ethanolamide, docosapentaenoate (n6 dpa; 22:5n6), sphingomyelin (d17:1/14:0, d16:1/15:0), ceramide (d16:1/24:1, d18:1/22:1), 1-methylurate, 1-carboxyethylisoleucine, 1-methylxanthine, sphinganine, n6-acetyllysine, n6-carbamoylthreonyladenosine, 5-acetylamino-6-amino-3-methyluracil, 1-arachidonylglycerol (20:4), homocitrulline, alpha-hydroxyisocaproate, oleoyl-arachidonoyl-glycerol (18:1/20:4) [2], 1,3,7-trimethylurate, caffeine, s-adenosylhomocysteine (sah), sphingosine, alpha-hydroxyisovalerate, n-stearoyltaurine, gamma-glutamylphenylalanine, 3-carboxy-4-methyl-5-pentyl-2-furanpropionate (3-cmpfp), n1-methylinosine, n2,n2-dimethylguanosine, behenoyl dihydrosphingomyelin (d18:0/22:0), theobromine, paraxanthine, glycerol, 1,3-dimethylurate, c-glycosyltryptophan, 1-carboxyethylphenylalanine, gamma-glutamylisoleucine, xanthurenate, 3-(3-amino-3-carboxypropyl)uridine, kynurenate, isovalerylcarnitine (c5), 4-hydroxyglutamate, n-acetyl-beta-alanine, glutamate, tiglyl carnitine (c5), adrenate (22:4n6), lysine, urate, sphingomyelin (d18:0/20:0, d16:0/22:0), leucine, palmitoleate (16:1n7), 2-stearoyl-gpe (18:0), 5-methylthioadenosine (mta), 1-(1-enyl-palmitoyl)-2-arachidonoyl-gpe (p-16:0/20:4), 1-arachidonoyl-gpi (20:4), uridine, hydroxyproline, xanthosine, sphingomyelin (d17:1/16:0, d18:1/15:0, d16:1/17:0), isoleucine, urea, 1-dihomo-linolenylglycerol (20:3), sphingomyelin (d18:0/18:0, d19:0/17:0), beta-cryptoxanthin, glycohyocholate, s-methylmethionine
PC2	8.12	19.08	arabonate/xylonate, guaiacol sulfate, pentose acid, erythronate, erythritol, ascorbic acid 3-sulfate, 1-ribosyl-imidazoleacetate, 2,3-dihydroxyisovalerate, ribulonate/xylulonate/lyxonate, myo-inositol, arabitol/xylitol, 4-acetamidobutanoate, n2,n5-diacetylornithine, vanillic acid glycine, pantoate, dopamine 3-o-sulfate, 2,3-dihydroxy-5-methylthio-4-pentenoate (dmtpa), 3-acetylphenol sulfate, citrulline, catechol sulfate, pseudouridine, 4-acetylcatechol sulfate (1), n-acetylthreonine, lanthionine, dopamine 4-sulfate, gulonate, hydroxy-n6,n6,n6-trimethyllysine, gamma-glutamylisoleucine, n6-succinyladenosine, 3-indoleglyoxylic acid, vanillactate, 3-(3-amino-3-carboxypropyl)uridine, creatinine, n,n,n-trimethyl-alanylproline betaine (tmap), 4-hydroxyphenylacetylglutamine, citraconate/glutaconate, o-cresol sulfate, 5-methylthioribose, methionine sulfone, alpha-ketoglutaramate, pyrraline, hydroxyasparagine, dihydrocaffeate sulfate (2), n1-methylinosine, indolelactate, trigonelline (n'-methylnicotinate), 4-guanidinobutanoate, histidine betaine (hercynine), 3-(3-hydroxyphenyl)propionate sulfate, 3-hydroxyhippurate, n-delta-acetylornithine, mannitol/sorbitol, 2-methylmalonylcarnitine (c4-dc), 3-hydroxy-3-methylglutarate, n6-carbamoylthreonyladenosine, 2,6-dihydroxybenzoic acid, 5,6-dihydrouridine, 2,3-dihydroxy-2-methylbutyrate, cytosine, ribonate, 3-amino-2-piperidone, 3-hydroxyhippurate sulfate, homovanillate (hva), ascorbic acid 2-sulfate, 4-hydroxyhippurate, 1,2-dilinoleoyl-gpe (18:2/18:2), ornithine, 2-aminophenol sulfate, vanillylmandelate (vma), 3-hydroxy-2-methylpyridine sulfate, n-formylmethionine, gamma-glutamylphenylalanine, 3-methoxycatechol sulfate (2), sphingomyelin (d18:0/18:0, d19:0/17:0)
PC3	7.82	26.9	tetradecadienoate (14:2), linoleate (18:2n6), docosadienoate (22:2n6), dihomolinoleate (20:2n6), trans-2-hexenoylglycine, eicosenoate (20:1n9 or 1n11), linolenate (18:3n3 or 3n6), dodecadienoate (12:2), 3-hydroxydodecanedioate, 3-hydroxydecanoate, dodecenedioate (c12:1-dc), oleate/vaccenate (18:1), n-oleoyltaurine, tetradecadienedioate (c14:2-dc), (2 or 3)-decenoate (10:1n7 or n8), 16-hydroxypalmitate, glutamine conjugate of c6h10o2 (2), 3-hydroxylaurate, hexadecanedioate (c16), 3-hydroxysebacate, arachidate (20:0), n-oleoylserine, hexanoylglutamine, erucate (22:1n9), cis-4-decenoate (10:1n6), octadecenedioate (c18:1-dc), oleoyl ethanolamide, 3-hydroxyoctanoate, hexadecadienoate (16:2n6), octadecanedioate (c18), dodecanedioate (c12), malate, citrate, 13-hode + 9-hode, 3-hydroxyhexanoate, linoleoyl ethanolamide, tetradecanedioate (c14), docosatrienoate (22:3n3), 3-hydroxybutyrate (bhba), n-linoleoyltaurine, 3beta-hydroxy-5-cholestenoate, nonadecanoate (19:0), 5-dodecenoate (12:1n7), branched-chain, straight-chain, or cyclopropyl 12:1 fatty acid, 2-hydroxypalmitate, 1-linoleoyl-gpi (18:2), 3-hydroxybutyroylglycine, docosapentaenoate (dpa; 22:5n3), 2-butenoylglycine, n-linoleoylglycine, dihomolinolenate (20:3n3 or 3n6), palmitate (16:0), n-palmitoylglycine, 2-hydroxynervonate, stearate (18:0), 3-hydroxymyristate, glycerol, succinate, aconitate [cis or trans], hexadecenedioate (c16:1-dc), glutamine conjugate of c6h10o2 (1), 4-chlorobenzoic acid, 12,13-dihome, palmitoleoylcarnitine (c16:1), 1-(1-enyl-stearoyl)-2-arachidonoyl-gpe (p-18:0/20:4), 1-methylurate, propionylcarnitine (c3), 5-acetylamino-6-amino-3-methyluracil,

			mannose, sulfate of piperine metabolite c18h21no3 (1), glutarylcarnitine (c5-dc), sulfate of piperine metabolite c18h21no3 (3), 7-methylxanthine, sulfate of piperine metabolite c16h19no3 (2), sulfate of piperine metabolite c16h19no3 (3), 1-(1-enyl-palmitoyl)-2-arachidonoyl-gpe (p-16:0/20:4), piperine
PC4	5.33	32.23	2-methylcitrate/homocitrate, 2-palmitoyl-gpc (16:0), 1-myristoyl-2-arachidonoyl-gpc (14:0/20:4), 1-stearoyl-2-oleoyl-gpi (18:0/18:1), 4-cholesten-3-one, 1-palmitoleoyl-2-linolenoyl-gpc (16:1/18:3), oleoyl-arachidonoyl-glycerol (18:1/20:4) [2], palmitoleoyl-linoleoyl-glycerol (16:1/18:2) [1], 1-oleoyl-2-linoleoyl-gpe (18:1/18:2), 1-palmitoyl-2-arachidonoyl-gpe (16:0/20:4), 1-linolenoylglycerol (18:3), 1-stearoyl-2-oleoyl-gpc (18:0/18:1), 1-palmitoyl-2-oleoyl-gpe (16:0/18:1), palmitoleoyl-arachidonoyl-glycerol (16:1/20:4) [2], 1-stearoyl-2-arachidonoyl-gpe (18:0/20:4), 1-palmitoyl-2-oleoyl-gpc (16:0/18:1), oleoyl-oleoyl-glycerol (18:1/18:1) [2], 1-stearoyl-2-linoleoyl-gpi (18:0/18:2), 1-palmitoyl-2-oleoyl-gpi (16:0/18:1), 1-palmitoleoyl-gpc (16:1), 2,4-di-tert-butylphenol, 1-stearoyl-gpc (18:0), linoleoyl-linolenoyl-glycerol (18:2/18:3) [2], 1-palmitoyl-2-linoleoyl-gpc (16:0/18:2), 1-palmitoyl-2-linoleoyl-gpe (16:0/18:2), 1-palmitoyl-2-dihomo-linolenoyl-gpc (16:0/20:3n3 or 6), 1-palmitoyl-2-linoleoyl-gpi (16:0/18:2), oleoyl-linoleoyl-glycerol (18:1/18:2) [1], 2-stearoyl-gpe (18:0), 1-stearoyl-2-arachidonoyl-gpi (18:0/20:4), 1-stearoyl-2-oleoyl-gpe (18:0/18:1), 1-palmitoyl-2-arachidonoyl-gpi (16:0/20:4), oleoyl-linoleoyl-glycerol (18:1/18:2) [2], 1-palmitoyl-gpe (16:0), alpha-tocopherol, 1-linolenoyl-gpc (18:3), 1-palmitoyl-gpc (16:0), 1-stearoyl-2-linoleoyl-gpe (18:0/18:2), 1-stearoyl-gpe (18:0)
PC5	3.77	36	1-(1-enyl-oleoyl)-gpe (p-18:1), sphingomyelin (d18:1/20:1, d18:2/20:0), sphingomyelin (d18:1/22:1, d18:2/22:0, d16:1/24:1), sphingomyelin (d18:2/23:0, d18:1/23:1, d17:1/24:1), lactosyl-n-behenoyl-sphingosine (d18:1/22:0), palmitoyl sphingomyelin (d18:1/16:0), sphingomyelin (d18:2/16:0, d18:1/16:1), sphingomyelin (d18:1/22:2, d18:2/22:1, d16:1/24:2), 1-stearoyl-2-linoleoyl-gpc (18:0/18:2), sphingomyelin (d18:2/21:0, d16:2/23:0), 1-(1-enyl-stearoyl)-gpe (p-18:0), 1-oleoyl-gps (18:1), 1-stearoyl-2-oleoyl-gps (18:0/18:1), tricosanoyl sphingomyelin (d18:1/23:0), taurine, 1-palmitoleoylglycerol (16:1)

[1]Based on principal components analysis of 930 log transformed metabolites. [2]Metabolites appear in order of loadings, with metabolites with highest loadings listed first.

Table S21. Adjusted linear regression predicting red meat, processed, and total meat consumption from top principal components from principal components analysis, with adjustment for additional potential dietary confounders.

	β	SE	T value	*p* value	Correlation coefficient	P value (Correlation)
**Red meat, model 1**[1]						
PC1	0.51	1.44	0.35	0.72	0.12	0.28
PC3	−2.01	1.48	−1.36	0.18		
PC4	0.99	1.13	0.88	0.38		
**Red meat, model 2**[2]						
PC1	0.45	0.84	0.53	0.6	0.17	0.12
PC3	−0.96	0.82	−1.18	0.24		
PC4	1.71	0.64	2.67	0.009		
**Total meat, model** [3]						
PC1	1.02	1.12	0.91	0.37	0.21	0.055
PC3	−2.19	1.14	−1.92	0.06		
PC4	0.69	0.88	0.79	0.43		
**Total meat, model 2**[2]						
PC1	0.38	0.71	0.54	0.59	0.32	0.003
PC2	−1.6	0.68	−2.34	0.022		
PC3	−1.73	2.51	−0.69	0.49		
PC4	1.47	0.54	2.71	0.0082		
**Processed meat, model 1**[3]						
PC1	0.9	0.43	2.11	0.038	0.24	0.026
PC3	0.07	0.45	0.15	0.88		
PC4	0.34	0.34	0.99	0.33		
**Processed meat, model 2**[2]						
PC1	0.08	0.46	0.17	0.87	0.2	0.07
PC3	0.47	0.45	1.03	0.31		
PC4	0.51	0.37	1.4	0.17		

[1]Adjusted for fish, poultry, dairy, and fruit consumption besides race, sex, age, BMI. [2]Adjusted for daily intake of fiber and saturated fat, in addition to race, sex, age, and BMI. [3]Adjusted for fish, dairy, and fruit consumption, race, sex, age, BMI.

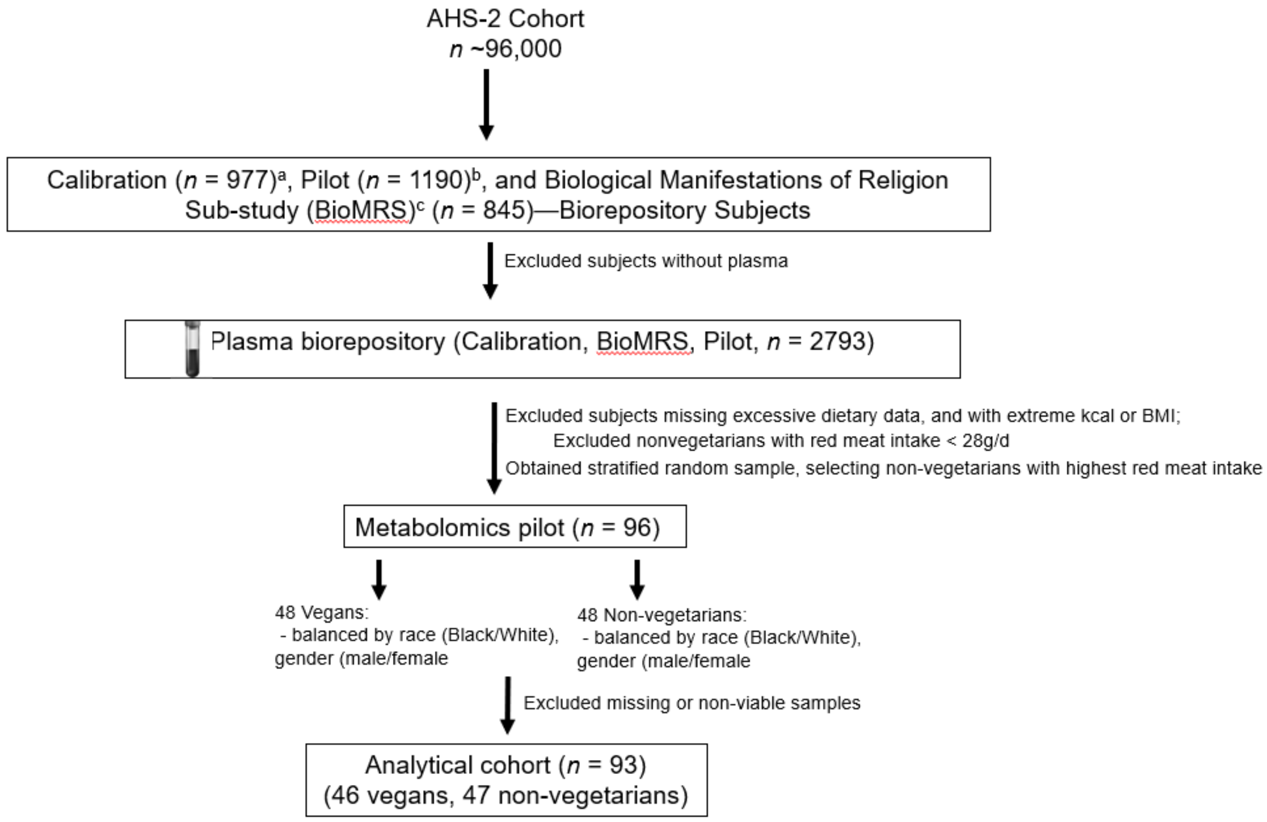

Figure S1. Study design for individuals in the Metabolomics Pilot Study. Footnotes: [a]The Calibration sub-study was a random sample of the cohort, except for an overweighting of Black subjects so they formed 40% of the total. [b]The two pilot sub-studies were convenience samples of study subjects living in Texas (Black subjects) or Washington State designed to test our bio-sample acquisition strategies. [c]The BioMRS sub-study was a local sample of AHS-2 subjects who had responded to a request to complete a questionnaire containing psychosocial and religiosity questions. They lived within 50 miles of Loma Linda, Riverside, or downtown Los Angeles and were at least 50 years of age.

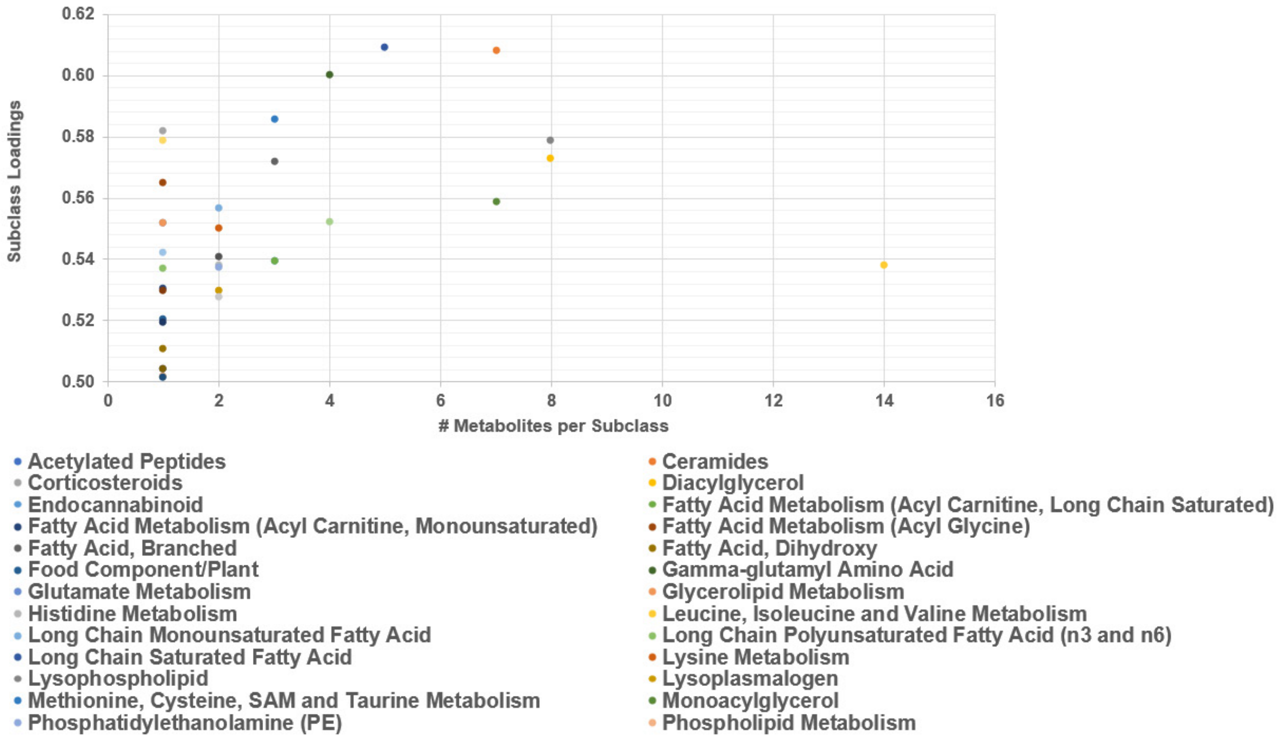

Figure S2. Subclass loadings from first principal component (PC1). Principal component analysis was used to identify components explaining variation in metabolites comparing vegans and nonvegetarians. Metabolite loadings > 0.5 were extracted and averaged across each represented subclass.
